# Light‐Controlled Destruction and Assembly: Switching between Two Differently Composed Cage‐Type Complexes

**DOI:** 10.1002/anie.202212571

**Published:** 2022-11-29

**Authors:** Daniel Hugenbusch, Marc Lehr, Jan‐Simon von Glasenapp, Anna J. McConnell, Rainer Herges

**Affiliations:** ^1^ Otto-Diels-Institute of Organic Chemistry Christian-Albrechts-Universität zu Kiel Otto-Hahn-Platz 4 24118 Kiel Germany

**Keywords:** Diazocine, Isomerization, Photochemistry, Self-Assembly, Supramolecular Chemistry

## Abstract

We report on two regioisomeric, diazocine ligands **1** and **2** that can both be photoswitched between the *E‐* and *Z*‐configurations with violet and green light. The self‐assembly of the four species (**1**‐*Z*, **1**‐*E*, **2**‐*Z*, **2**‐*E*) with Co^II^ ions was investigated upon changing the coordination vectors as a function of the ligand configuration (*E* vs *Z*) and regioisomer (**1** vs **2**). With **1**‐*Z*, Co_2_(**1**‐*Z*)_3_ was self‐assembled, while a mixture of ill‐defined species (oligomers) was observed with **2**‐*Z*. Upon photoswitching with 385 nm to the *E* configurations, the opposite was observed with **1**‐*E* forming oligomers and **2**‐*E* forming Co_2_(**2**‐*E*)_3_. Light‐controlled dis/assembly was demonstrated in a ligand competition experiment with sub‐stoichiometric amounts of Co^II^ ions; alternating irradiation with violet and green light resulted in the reversible transformation between Co_2_(**1**‐*Z*)_3_ and Co_2_(**2**‐*E*)_3_ over multiple cycles without significant fatigue by photoswitching.

## Graphical abstract

Upon irradiation, metal complexes rise like a phoenix from the ashes. In a mixture of two different photoswitchable ligands (red and blue) and Co^2+^ ions, a Co_2_L_3_ structure forms from only the blue ligands. Upon irradiation with violet light, both ligands isomerize and the blue Co_2_L_3_ disassembles. Now the red ligand forms the red Co_2_L_3_ structure. Irradiation with green light destroys this complex and the first re‐assembles. The process is fully reversible.

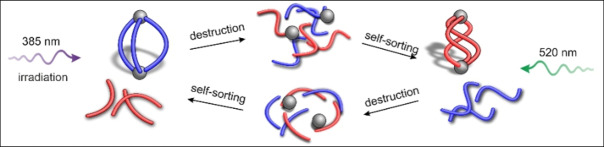

Triggered and controlled assembly and disassembly are among the most fundamental processes in living systems. For instance, light‐triggered *cis* to *trans* isomerisation of retinal inside rhodopsin ultimately leads to the macroscopic perception of light following several consecutive processes, including the assembly of two rhodopsins with transducin and disassembly of the heterotrimeric transducin into two of its three components.^[1]^ Stimulus‐controlled assembly and disassembly are ubiquitous steps in biological signal transduction cascades. In natural and synthetic systems, light is an almost ideal stimulus and control tool since it can be applied with high spatiotemporal resolution with different wavelengths, and it is typically waste‐ and by‐product‐free.^[2]^ Light has been exploited to trigger the self‐assembly of nanoparticles,^[3]^ polymers^[4]^ and gels[^4a^, ^5^] as well as to drive the folding of polymers^[6]^ and the operation of molecular machines^[7]^ and shuttles.^[8]^

Despite the numerous examples of stimuli‐responsive metal‐organic cages and helicates,[^2^, ^9^] light‐responsive examples are relatively rare and include the use of photoswitchable guests and counterions^[10]^ as well as photo‐induced metal‐ligand bond breakage,^[11]^ photolabile groups^[12]^ or photoacids^[13]^ to trigger the self‐assembly/disassembly of cages. Photoswitchable cages^[2]^ based on azobenzene,^[14]^ dithienylethene,^[15]^ overcrowded alkenes,^[16]^ and more recently, diazocine^[17]^ have been reported but are challenging to design; for example, incomplete photoswitching (from overlapping n‐π* and π‐π* bands or overlap with the metal's MLCT band) can lead to isomeric mixtures and large ligand geometry changes upon photoswitching can produce ill‐defined self‐assemblies.[^2^, ^9a^, ^14b^] Beves et al. recently reported on a photoswitchable, azobenzene‐based Pd complex that switches between two different complex geometries and stoichiometries upon irradiation with two different wavelengths of visible light.^[14c]^

Recently, diazocines (bridged azobenzenes) were developed as a new type of photoswitch with promising properties for applications, ranging from photopharmacology^[18]^ to switchable surfaces^[19]^ and smart materials.^[20]^ The good separation of n‐π* and π‐π* bands, efficient photoswitching with visible light and the thermodynamic stability of the *Z* configuration (as opposed to the *E* configuration for azobenzenes) make diazocines appealing for the design of photoswitchable cages. However, the development of diazocine‐based, photoswitchable cages is in its infancy. Clever et al. recently reported on a photoresponsive, diazocine‐based Pd_2_L_4_ cage.^[17]^ Upon irradiation with two different wavelengths, the complex reversibly changes its cage volume and binds or releases a guest.

We report that violet and green light direct the outcome of self‐assembly in a competition experiment with Co^II^ ions and a mixture of the two photoswitchable regioisomeric diazocine ligands, **1** and **2** (Figure 1). In their thermodynamically stable *Z* form, Co_2_(**1**‐*Z*)_3_ is self‐assembled since **2**‐*Z* does not form discrete self‐assemblies with Co^II^ ions. Upon irradiation with violet light (385 nm) ligands **1** and **2** simultaneously switch to their metastable *E* form and metastable Co_2_(**2**‐*E*)_3_ is self‐assembled since ligand **1**‐*E* forms ill‐defined complexes with Co^II^. Irradiation with green light (520 nm) switches both ligands simultaneously back to their original *Z* configuration, restoring the initial Co_2_(**1**‐*Z*)_3_. Thus, we report the reversible photoswitching between Co_2_(**1**‐*Z*)_3_ and Co_2_(**2**‐*E*)_3_ without fatigue over a number of cycles. To the best of our knowledge, it is the first example of a reversible light‐controlled transformation between two distinct metallosupramolecular structures including different ligands via dis/assembly.

**Figure 1:**
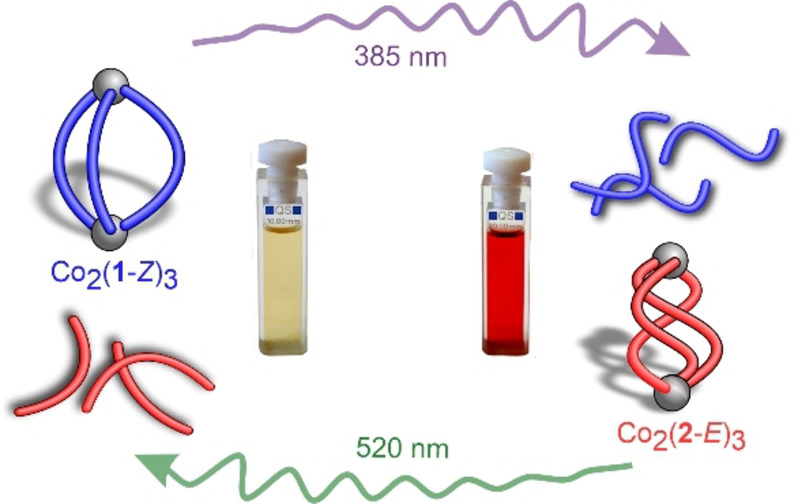
Reversible light‐controlled assembly and disassembly of Co_2_(**1**‐*Z*)_3_ and Co_2_(**2**‐*E*)_3_. Self‐assembly is directed with violet and green light. Ligands **1** and **2** are represented as blue and red “sausages”. Co^2+^ ions are grey spheres. *Z* isomers (*cis* azo group) have a C shape and *E* isomers (*trans*) an S shape. For an assignment to the corresponding chemical structures and DFT optimized geometries, see Figure 2. For the 2D chemical structures of **1** and **2** see Figure 3.

Ligands **1** and **2** each consist of a diazocine‐backbone and two 2‐pyridyl‐triazole units attached to both benzene rings (Figure 2). They differ in the point of attachment of the two pyridyl‐triazole groups (*para* with respect to the azo group in **1** and *meta* in **2**), which gives rise to different coordination vectors (bite angles ≈61 and 63°). An even stronger change in their bite angles is achieved upon light‐induced *Z*→*E* isomerization (≈137 and 110°).

**Figure 2:**
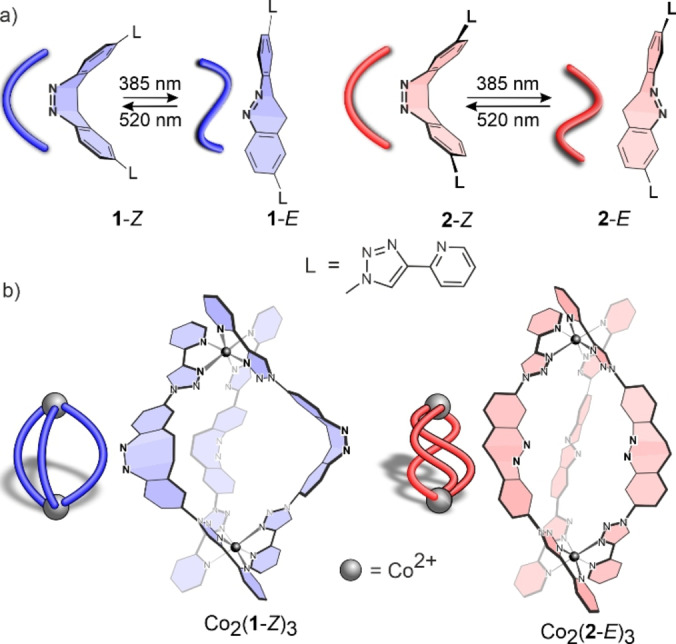
a) Photoswitchable diazocine‐based, regioisomeric ligands **1** and **2** in their *Z* and *E* configurations. b) Co_2_(**1**‐*Z*)_3_ and Co_2_(**2**‐*E*)_3_ are the only defined self‐assemblies formed from the four possible ligands (**1**‐*Z*, **1**‐*E*, **2**‐*Z*, **2**‐*E*) and Co^II^ ions. Ligands **1**‐*E* and **2**‐*Z* formed ill‐defined complexes with Co^II^ ions. Note in (b) double bonds are omitted for clarity and the ligand “sausage” representations highlight the ligand configuration rather than the stereochemistry of the metal complex.

First, we investigated the photophysical properties of the diazocine ligands and their coordination behaviour independently (Figure 2). Since the formation of multiple species could be expected in the case of incomplete photoswitching,^[14b]^ Co^II^ was chosen as the metal ion to reduce signal overlap by exploiting the large paramagnetic shifts in the ^1^H NMR spectra.^[21]^ The two ligands were prepared by analogous synthetic strategies (Scheme S1) from either literature‐known *p*‐diiododiazocine^[22]^ in two steps (ligand **1**) or 2‐nitro‐4‐iodotoluene in 5 steps (ligand **2**).

The photoswitching properties of ligands **1** and **2** were investigated by NMR (Figures S41, S42, S45, and S46) and UV/Vis spectroscopy (Figures S43, S44, S47 and S48). The ligands were converted from their thermodynamically stable *Z* configuration into the corresponding *E* form by irradiation with light of 385 nm. The photostationary state (PSS) was determined to be 76 % (*E* isomer) for ligand **1** and 72 % for ligand **2** (Figures S42, S46). The thermal half‐life (*t*_1/2_) is 2.0 h for ligand **1** and 5.5 h for ligand **2** at 25 °C (Figures S44, S48).

The self‐assembly of the ligands (**1**‐*Z*, **1**‐*E*, **2**‐*Z*, **2**‐*E*) with Co(BF_4_)_2_ (2 : 3 metal/ligand ratio) was carried out at room temperature, in particular to prevent relaxation of any metastable self‐assemblies composed of **1**‐*E* and **2**‐*E* (Supporting Information Section 3). Complex formation occurs within minutes at room temperature and upon heating the solutions including ligands in their thermodynamically stable *Z* configurations (**1**‐*Z* and **2**‐*Z*) to 50 °C, no change of the NMR spectra was observed (Figures S30, S40). Discrete self‐assemblies were isolated by precipitation with diethyl ether.

The self‐assembly of ligand **1**‐*Z* with Co(BF_4_)_2_ exhibits 10 NMR signals within a chemical shift range of approximately 85 ppm (Figure 3a). All signals could be assigned using a combination of paramagnetic NMR methods (Figure 3a).^[21]^ The fact that the complex exhibits the same number of signals as the free ligand hints at the formation of a highly symmetrical species. The ion peaks and isotope pattern in the ESI mass spectrum (Figure S29) are consistent with the 2 : 3 Co^II^/ligand complex Co_2_(**1**‐*Z*)_3_. According to NMR experiments, the complex is stable upon dilution to the concentrations required for ESI and UV/Vis measurements (Figure S51).

**Figure 3:**
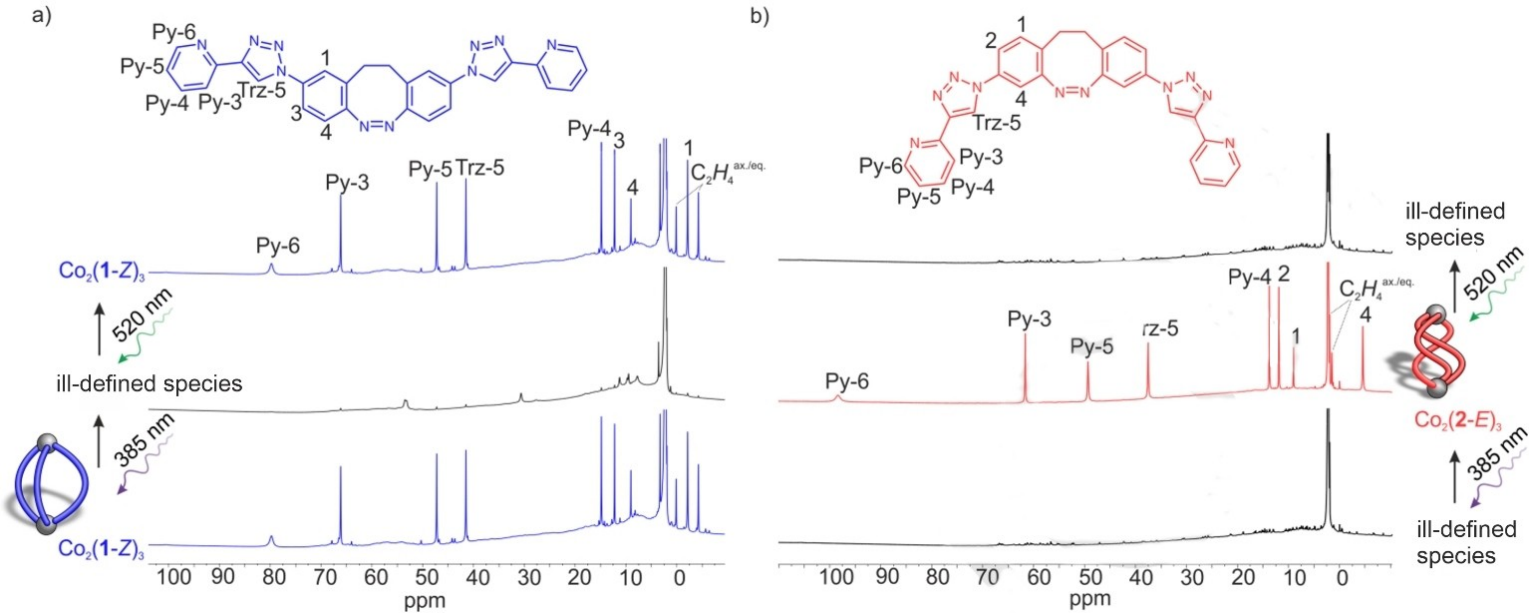
^1^H NMR spectra (CD_3_CN, 298 K) of the reversible transformation between: a) Co_2_(**1**‐*Z*)_3_ (bottom and top) and the ill‐defined species (oligomers) formed with Co^II^ and **1**‐*E* (middle) following irradiation with light at 385 nm and 520 nm, respectively; b) the ill‐defined mixture formed with Co^II^ and **2**‐*Z* (top and bottom) and Co_2_(**2**‐*E*)_3_ (middle) following irradiation with light at 385 nm and 520 nm, respectively. Paramagnetic shifts and line broadening are due to the paramagnetic Co^II^ ions.

Photoswitching of Co_2_(**1**‐*Z*)_3_ was investigated by irradiation with 385 nm (Figure S52). During irradiation, the ^1^H NMR signals assigned to Co_2_(**1**‐*Z*)_3_ decrease in intensity and new broad signals are observed (Figure 3a). Only very small signals of the original Co_2_(**1**‐*Z*)_3_ remain in the photostationary equilibrium (PSS), which we attribute to the incomplete photoswitching of ligand **1**‐*Z* to **1**‐*E*. The broad signals hint at a mixture of ill‐defined species, suggesting the formation of polymers and oligomers. After standing of this solution at room temperature for several hours, the intensities of the ^1^H NMR signals for Co_2_(**1**‐*Z*)_3_ increase again because **1**‐*E* thermally relaxes back to **1**‐*Z* (Figure S50). The thermal half‐life (*t*_1/2_) of ligand **1**‐*E* in the presence of Co^II^ ions was measured to be 1.6 h (Figures S52, S53). This half‐life is 24 % shorter compared to the free ligand (2.0 h, Figures S43, S44), suggesting the thermal relaxation process is influenced by the metal ions.

Transformation of the ill‐defined mixture including ligand **1**‐*E* to the discrete helicate Co_2_(**1**‐*Z*)_3_ was also achieved by irradiation with green light (520 nm). The conversion is complete within the detection limits of NMR spectroscopy. Photoswitching between the ill‐defined mixture and Co_2_(**1**‐*Z*)_3_ with 385 and 520 nm is reversible without significant fatigue for 20 cycles by UV/Vis (Figure S54) and ^1^H NMR (Figure S49) spectroscopy.

Ligand **2** behaves exactly the opposite of ligand **1** in the presence of Co^II^ ions. While ligand **1**‐*Z* formed the helicate Co_2_(**1**‐*Z*)_3_, **2**‐*Z* yields a ill‐defined mixture (oligomers) (Figure 2b); in contrast to oligomer formation with **1**‐*E*, the regioisomer **2**‐*E* forms the helicate Co_2_(**2**‐*E*)_3_ (Figure 3 and Figure S38). Co_2_(**2**‐*E*)_3_ is stable to isolation by precipitation (Supporting Information Section 3.2) and dilution (Figure S57). Co_2_(**2**‐*E*)_3_ is metastable in solution. The ill‐defined mixture is restored due to thermal relaxation of the ligand **2**‐*E* back to **2**‐*Z* (Figure S56). The half‐life was determined to be 9.2 h (Figures S58, S59). This is 67 % longer than the half‐life for the free ligand (5.5 h, Figures S47, S48). The formation of ill‐defined species shortens the half‐life of **1**‐*E* and the formation of a defined triple helicate increases the half‐life of ligand **2**‐*E*.

As in case of the ligand **1**/Co^II^ system, photoswitching between an ill‐defined mixture and a discrete helicate was observed, however, in the reverse direction. Irradiation of ligand **2** in the presence of Co^II^ ions (3 : 2 ratio) with 385 nm yields the discrete helicate Co_2_(**2**‐*E*)_3_ and with 520 nm light an ill‐defined mixture is formed (Figure 3b). No fatigue was observed over 20 cycles by UV/Vis (Figure S60) and ^1^H NMR (Figure S55) spectroscopies.

Hence, ligands **1**‐*Z* and **2**‐*E* form Co_2_L_3_ self‐assemblies, while ligands **1**‐*E* and **2**‐*Z* form ill‐defined mixtures. Despite numerous attempts, single crystals of Co_2_(**1**‐*Z*)_3_ or Co_2_(**2**‐*E*)_3_ could not be obtained. To gain more insight into the influence of the structures of the regioisomers and their configurations on the outcome of the self‐assembly, theoretical calculations were performed. In principle, complexes Co_2_(**1**‐*Z*)_3_ and Co_2_(**2**‐*E*)_3_ can exist as helicates or meso‐helicates (mesocates). The energies of the four conceivable structures were calculated at the ωB97X‐D3/def2‐TZVP level of theory using ORCA 5.0.1 (Supporting Information Section 7).^[23]^ In both cases, the helicate was lower in energy (1.2 and 1.8 kcal mol^−1^) than the mesocate, and thus, we conclude that Co_2_(**1**‐*Z*)_3_ and Co_2_(**2**‐*E*)_3_ are helicates (Table S4). Helicate structures were also calculated for ligands **1**‐*E* and **2**‐*Z* and the ligand strain energies in all four helicates were compared (Table S6). Of the ligands in the *Z* configuration (**1**‐*Z* and **2**‐*Z*), Co_2_(**1**‐*Z*)_3_ has a lower ligand strain energy than Co_2_(**2**‐*Z*)_3_, and with the ligands in the *E* configuration, the ligand strain energy for Co_2_(**2**‐*E*)_3_ is lower than Co_2_(**1**‐*E*)_3_. Ligand **1** is more susceptible to form a helicate in its *Z* configuration and ligand **2** prefers to form the helicate in its *E* configuration. This is in agreement with the experimental findings because Co_2_(**2**‐*Z*)_3_ and Co_2_(**1**‐*E*)_3_ were experimentally not observed (Figure 4).

**Figure 4:**
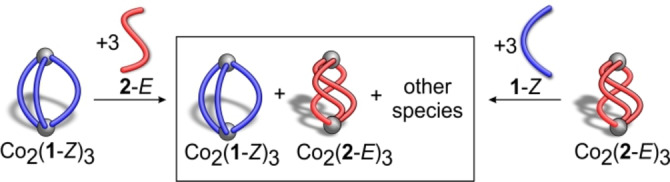
Competition experiments investigating the relative stabilities of Co_2_(**1**‐*Z*)_3_ and Co_2_(**2**‐*E*)_3_ upon addition of the regioisomeric ligand with the opposite configuration.

Our calculations also predict that the two experimentally observed helicates Co_2_(**1**‐*Z*)_3_ and Co_2_(**2**‐*E*)_3_ are close in energy. This was corroborated by two complementary experiments by adding: i) ligand **2**‐*E* to Co_2_(**1**‐*Z*)_3_ (Figures S61, S62); ii) ligand **1**‐*Z* to Co_2_(**2**‐*E*)_3_ (Figure S63, S64). In both experiments, ligand addition resulted in the formation of a mixture of both Co_2_(**1**‐*Z*)_3_ and Co_2_(**2**‐*E*)_3_ (Figure 4).

Having demonstrated that discrete self‐assemblies are obtained with regioisomeric ligands of the opposite configuration, we envisaged a system where light could be exploited to switch between Co_2_(**1**‐*Z*)_3_ and Co_2_(**2**‐*E*)_3_, i.e. between helicates of different compositions (Figure 1). In the initial mixture with **1**‐*Z* and **2**‐*Z*, Co_2_(**1**‐*Z*)_3_ forms as the major self‐assembled species, as observed by ^1^H NMR spectroscopy (Figures 5a and S65). Upon irradiation with 385 nm the signals of Co_2_(**1**‐*Z*)_3_ disappear and the signals of Co_2_(**2**‐*E*)_3_ grow in. Irradiation with 520 nm induces disassembly of Co_2_(**2**‐*E*)_3_ and restores the assembly of Co_2_(**1**‐*Z*)_3_ (Figures 5a and S65). Alternating irradiation with 385 nm and 520 nm demonstrated that the transformation between the two helicates is reversible over at least 20 cycles without significant fatigue (Figure 5b).

**Figure 5:**
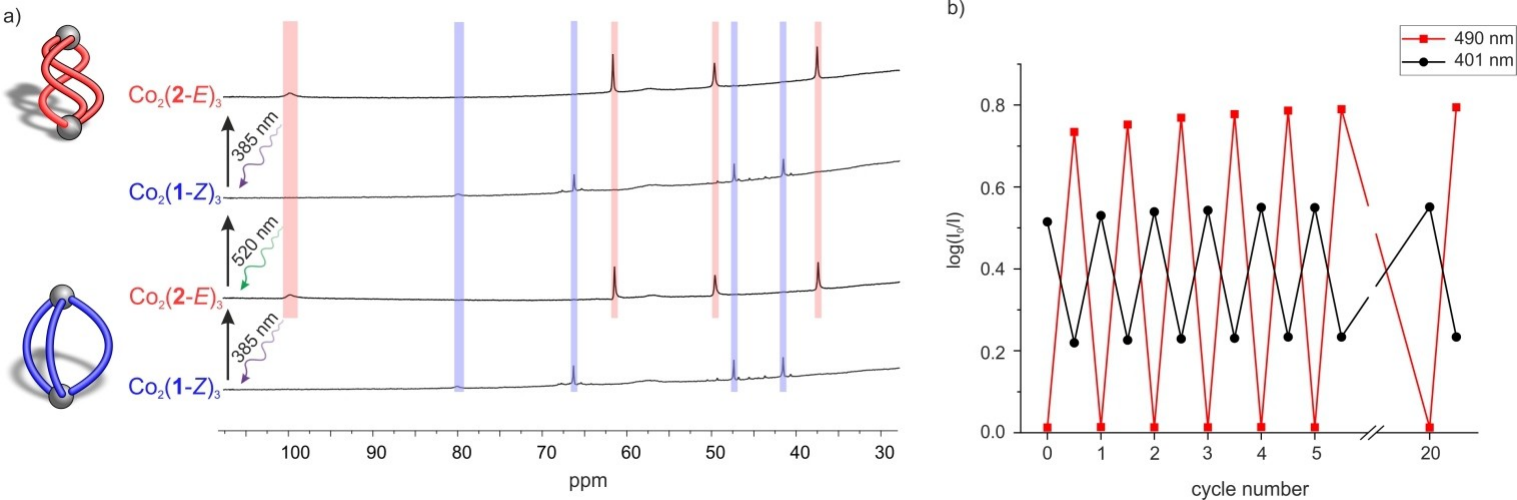
Reversible light‐controlled assembly and disassembly of Co_2_(**1**‐*Z*)_3_ and Co_2_(**2**‐*E*)_3_. A solution of Co_2_(**1**‐*Z*)_3_ (a: 6 mM, b: 0.33 mM) was mixed with 3 equivalents of **2**‐*Z* and the solution was irradiated with light at 385 and 520 nm in an alternating sequence. a) Bottom spectrum: mainly signals of Co_2_(**1**‐*Z*)_3_ are observed (blue shaded). Second spectrum from the bottom: upon irradiation at 385 nm, the signals of Co_2_(**1**‐*Z*)_3_ disappear and the signals of Co_2_(**2**‐*E*)_3_ appear (red shaded). Third spectrum from the bottom: irradiation at 520 nm restores the signals of Co_2_(**1**‐*Z*)_3_ (blue shaded). Note: chemical shift regions below 30 ppm are omitted for clarity. Figure S65 shows the full spectra. Line broadening and the paramagnetic shifts of the ^1^H NMR signals are due to ligand coordination to paramagnetic Co^II^ ions. b) Switching stability experiment. The absorption at 401 nm (black) and 490 nm (red) is plotted after alternating irradiation at 385 and 520 nm. Switching is reversible without significant fatigue over 20 cycles.

In conclusion, we report on a self‐assembly system including two photoswitchable, diazocine‐based ligands (**1** and **2**) and Co^II^ ions. Both ligands switch to the *E* configuration upon irradiation with violet light (385 nm) and back to the *Z* isomer with green light (520 nm). Among the four possible species **1**‐*Z*, **1**‐*E*, **2**‐*Z* and **2**‐*E*, only **1**‐*Z* and **2**‐*E* form discrete cage‐type complexes (helicates) with Co^II^ ions: Co_2_(**1**‐*Z*)_3_ and Co_2_(**2**‐*E*)_3_. Upon irradiation with 385 nm, Co_2_(**1**‐*Z*)_3_ disintegrates and Co_2_(**2**‐*E*)_3_ assembles. Irradiation with 520 nm disassembles Co_2_(**2**‐*E*)_3_ and Co_2_(**1**‐*Z*)_3_ is simultaneously rebuilt. Switching between the two helicates of different composition exhibits no fatigue or side‐products over at least 20 switching cycles (Figure 1 and Figure 5b). Reorganization of multicomponent protein complexes triggered by external stimuli is an ubiquitous phenomenon in signal transduction pathways in nature. Our system might serve as a simple non‐biological example and pave the way to the development of more sophisticated systems for artificial signal transduction cascades.

## Conflict of interest

The authors declare no conflict of interest.

## Supporting information

As a service to our authors and readers, this journal provides supporting information supplied by the authors. Such materials are peer reviewed and may be re‐organized for online delivery, but are not copy‐edited or typeset. Technical support issues arising from supporting information (other than missing files) should be addressed to the authors.

Supporting Information

## Data Availability

The data that support the findings of this study are available in the Supporting Information of this article.

## References

[1] B. Jastrzebska , P. Ringler , K. Palczewski , A. Engel , J. Struct. Biol. 2013, 182, 164–172. 23458690 10.1016/j.jsb.2013.02.014 PMC3633645

[2] S. J. Wezenberg , Chem. Lett. 2020, 49, 609–615.

[3a] R. Klajn , K. J. M. Bishop , B. A. Grzybowski , Proc. Natl. Acad. Sci. USA 2007, 104, 10305–10309; 17563381 10.1073/pnas.0611371104 PMC1965508

[3b] D. Manna , T. Udayabhaskararao , H. Zhao , R. Klajn , Angew. Chem. Int. Ed. 2015, 54, 12394–12397; 10.1002/anie.201502419 25959725

[4a] F. Xu , L. Pfeifer , S. Crespi , F. K.-C. Leung , M. C. A. Stuart , S. J. Wezenberg , B. L. Feringa , J. Am. Chem. Soc. 2021, 143, 5990–5997; 33830767 10.1021/jacs.1c01802 PMC8154511

[4b] M. Chen , M. Zhong , J. A. Johnson , Chem. Rev. 2016, 116, 10167–10211; 26978484 10.1021/acs.chemrev.5b00671

[4c] F. D. Jochum , P. Theato , Chem. Soc. Rev. 2013, 42, 7468–7483. 22868906 10.1039/c2cs35191a

[5] F. A. Larik , L. L. Fillbrook , S. S. Nurttila , A. D. Martin , R. P. Kuchel , K. Al Taief , M. Bhadbhade , J. E. Beves , P. Thordarson , Angew. Chem. Int. Ed. 2021, 60, 6764–6770; 10.1002/anie.202015703 33295683

[6a] A. Ryabchun , Q. Li , F. Lancia , I. Aprahamian , N. Katsonis , J. Am. Chem. Soc. 2019, 141, 1196–1200; 30624915 10.1021/jacs.8b11558 PMC6346373

[6b] T. Fukushima , K. Tamaki , A. Isobe , T. Hirose , N. Shimizu , H. Takagi , R. Haruki , S.-i. Adachi , M. J. Hollamby , S. Yagai , J. Am. Chem. Soc. 2021, 143, 5845–5854; 33755463 10.1021/jacs.1c00592

[6c] A. Lendlein , H. Jiang , O. Jünger , R. Langer , Nature 2005, 434, 879–882. 15829960 10.1038/nature03496

[7a] P. Štacko , J. C. M. Kistemaker , T. van Leeuwen , M.-C. Chang , E. Otten , B. L. Feringa , Science 2017, 356, 964–968; 28572394 10.1126/science.aam8808

[7b] N. Koumura , R. W. J. Zijlstra , R. A. van Delden , N. Harada , B. L. Feringa , Nature 1999, 401, 152–155. 10490022 10.1038/43646

[8] D. A. Leigh , V. Marcos , T. Nalbantoglu , I. J. Vitorica-Yrezabal , F. T. Yasar , X. Zhu , J. Am. Chem. Soc. 2017, 139, 7104–7109. 28471662 10.1021/jacs.7b03307

[9a] A. J. McConnell , C. S. Wood , P. P. Neelakandan , J. R. Nitschke , Chem. Rev. 2015, 115, 7729–7793; 25880789 10.1021/cr500632f

[9b] E. Benchimol , B.-N. T. Nguyen , T. K. Ronson , J. R. Nitschke , Chem. Soc. Rev. 2022, 51, 5101–5135; 35661155 10.1039/d0cs00801j PMC9207707

[9c] M. J. Burke , G. S. Nichol , P. J. Lusby , J. Am. Chem. Soc. 2016, 138, 9308–9315; 27351912 10.1021/jacs.6b05364

[9d] T. Y. Kim , R. A. S. Vasdev , D. Preston , J. D. Crowley , Chem. Eur. J. 2018, 24, 14878–14890. 29939443 10.1002/chem.201802081

[10a] G. H. Clever , S. Tashiro , M. Shionoya , J. Am. Chem. Soc. 2010, 132, 9973–9975; 20604553 10.1021/ja103620z

[10b] L. Pesce , C. Perego , A. B. Grommet , R. Klajn , G. M. Pavan , J. Am. Chem. Soc. 2020, 142, 9792–9802; 32353237 10.1021/jacs.0c03444 PMC7644116

[10c] H. Sunohara , K. Koyamada , H. Takezawa , M. Fujita , Chem. Commun. 2021, 57, 9300–9302. 10.1039/d1cc03620c 34519311

[11] N. Kishi , M. Akita , M. Kamiya , S. Hayashi , H.-F. Hsu , M. Yoshizawa , J. Am. Chem. Soc. 2013, 135, 12976–12979. 23957216 10.1021/ja406893y

[12] A. J. McConnell , C. J. E. Haynes , A. B. Grommet , C. M. Aitchison , J. Guilleme , S. Mikutis , J. R. Nitschke , J. Am. Chem. Soc. 2018, 140, 16952–16956. 30465601 10.1021/jacs.8b11324

[13] S. M. Jansze , G. Cecot , K. Severin , Chem. Sci. 2018, 9, 4253–4257. 29780555 10.1039/c8sc01108g PMC5944229

[14a] E. Britton , R. J. Ansell , M. J. Howard , M. J. Hardie , Inorg. Chem. 2021, 60, 12912–12923; 34370947 10.1021/acs.inorgchem.1c01297

[14b] A. D. W. Kennedy , R. G. DiNardi , L. L. Fillbrook , W. A. Donald , J. E. Beves , Chem. Eur. J. 2022, 28, e202104461; 35102616 10.1002/chem.202104461 PMC9302685

[14c] R. DiNardi , A. O. Douglas , R. Tian , J. Price , M. Tajik , W. A. Donald , J. Beves , Angew. Chem. Int. Ed. 2022, 61, e202205701; 10.1002/anie.202205701 PMC9541570 35972841

[15a] M. Han , R. Michel , B. He , Y.-S. Chen , D. Stalke , M. John , G. H. Clever , Angew. Chem. Int. Ed. 2013, 52, 1319–1323; 10.1002/anie.201207373 23208865

[15b] M. Han , Y. Luo , B. Damaschke , L. Gómez , X. Ribas , A. Jose , P. Peretzki , M. Seibt , G. H. Clever , Angew. Chem. Int. Ed. 2016, 55, 445–449; 10.1002/anie.201508307 26609916

[15c] R.-J. Li , J. J. Holstein , W. G. Hiller , J. Andréasson , G. H. Clever , J. Am. Chem. Soc. 2019, 141, 2097–2103. 30620873 10.1021/jacs.8b11872

[16] C. Stuckhardt , D. Roke , W. Danowski , E. Otten , S. J. Wezenberg , B. L. Feringa , Beilstein J. Org. Chem. 2019, 15, 2767–2773. 31807210 10.3762/bjoc.15.268 PMC6880828

[17] H. Lee , J. Tessarolo , D. Langbehn , A. Baksi , R. Herges , G. H. Clever , J. Am. Chem. Soc. 2022, 144, 3099–3105. 35081312 10.1021/jacs.1c12011 PMC8874908

[18a] J. B. Trads , K. Hüll , B. S. Matsuura , L. Laprell , T. Fehrentz , N. Görldt , K. A. Kozek , C. D. Weaver , N. Klöcker , D. M. Barber , D. Trauner , Angew. Chem. Int. Ed. 2019, 58, 15421–15428; 10.1002/anie.201905790 31441199

[18b] M. Schehr , C. Ianes , J. Weisner , L. Heintze , M. P. Müller , C. Pichlo , J. Charl , E. Brunstein , J. Ewert , M. Lehr , U. Baumann , D. Rauh , U. Knippschild , C. Peifer , R. Herges , Photochem. Photobiol. Sci. 2019, 18, 1398–1407. 30924488 10.1039/c9pp00010k

[19] T. Tellkamp , J. Shen , Y. Okamoto , R. Herges , Eur. J. Org. Chem. 2014, 5456–5461.

[20] M. H. Burk , D. Langbehn , G. Hernández Rodríguez , W. Reichstein , J. Drewes , S. Schröder , S. Rehders , T. Strunskus , R. Herges , F. Faupel , ACS Appl. Polym. Mater. 2021, 3, 1445–1456.

[21] M. Lehr , T. Paschelke , E. Trumpf , A.-M. Vogt , C. Näther , F. D. Sönnichsen , A. J. McConnell , Angew. Chem. Int. Ed. 2020, 59, 19344–19351; 10.1002/anie.202008439 PMC7590057 33448544

[22] M. S. Maier , K. Hüll , M. Reynders , B. S. Matsuura , P. Leippe , T. Ko , L. Schäffer , D. Trauner , J. Am. Chem. Soc. 2019, 141, 17295–17304. 31584272 10.1021/jacs.9b08794

[23a] F. Weigend , R. Ahlrichs , Phys. Chem. Chem. Phys. 2005, 7, 3297–3305; 16240044 10.1039/b508541a

[23b] F. Weigend , Phys. Chem. Chem. Phys. 2006, 8, 1057–1065; 16633586 10.1039/b515623h

[23c] F. Neese , F. Wennmohs , A. Hansen , U. Becker , Chem. Phys. 2009, 356, 98–109;

[23d] S. Grimme , J. Antony , S. Ehrlich , H. Krieg , J. Chem. Phys. 2010, 132, 154104; 20423165 10.1063/1.3382344

[23e] Y.-S. Lin , G.-D. Li , S.-P. Mao , J.-D. Chai , J. Chem. Theory Comput. 2013, 9, 263–272; 26589028 10.1021/ct300715s

[23f] M. Miklitz , K. E. Jelfs , J. Chem. Inf. Model. 2018, 58, 2387–2391; 30199639 10.1021/acs.jcim.8b00490 PMC6307083

[23g] C. Bannwarth , S. Ehlert , S. Grimme , J. Chem. Theory Comput. 2019, 15, 1652–1671; 30741547 10.1021/acs.jctc.8b01176

[23h] F. Neese , F. Wennmohs , U. Becker , C. Riplinger , J. Chem. Phys. 2020, 152, 224108; 32534543 10.1063/5.0004608

[23i] C. Bannwarth , E. Caldeweyher , S. Ehlert , A. Hansen , P. Pracht , J. Seibert , S. Spicher , S. Grimme , WIREs Comput. Mol. Sci. 2021, 11, e1493.

