# Supplementary material for: Light‐Controlled Destruction and Assembly: Switching between Two Differently Composed Cage‐Type Complexes
Source: Angew Chem Int Ed Engl. 2022 Nov 29;62(1):e202212571. doi: 10.1002/anie.202212571 (PMC10099457; doi:10.1002/anie.202212571)
Supplement: Supplementary file 1 — Supporting Information [file ANIE-62-0-s001.pdf]

Angewandte Chemie
Eine Zeitschrift der Gesellschaft Deutscher Chemiker

Supporting Information

Light-Controlled Destruction and Assembly: Switching between Two Differently Composed Cage-Type Complexes

D. Hugenbusch, M. Lehr, J.-S. von Glasenapp, A. J. McConnell, R. Herges**

Table of Contents

1. General Methods 4
2. Synthesis 6
2.1 Ligand 2 7
2.1.1 1,2-Bis(4-iodo-2-nitrophenyl)ethane (3) 7
2.1.2 1,2-Bis(4-iodo-2-aminophenyl)ethane (4) 9
2.1.3 3,8-Diiodo-11,12-dihydrodibenzo[*c,g*][1,2]diazocine (5) 11
2.1.4 3,8-Diazido-11,12-dihydrodibenzo[*c,g*][1,2]diazocine (6) 13
2.1.5 3,8-Bis(4-pyridin-2-yl)-1*H*-1,2,3-triazol-1-yl)-11,12-dihydrodibenzo[*c,g*][1,2] diazocine (2) 15
2.2 Ligand 1 21
2.2.1 2,9-Diiodo-11,12-dihydrodibenzo[*c,g*][1,2]diazocine (7) 21
2.2.2 2,9-Diazido-11,12-dihydrodibenzo[*c,g*][1,2]diazocine (8) 23
2.2.3 2,9-Bis(4-pyridin-2-yl)-1*H*-1,2,3-triazol-1-yl)-11,12-dihydrodibenzo[*c,g*][1,2] diazocine (1) 25
3. Self-Assemblies 31
3.1 $Co_2(1\text{-}Z)_3$ 31
3.2 Mixture of Ill-Defined Species with 1-*E* and $Co(BF_4)_2$ 35
3.3 $Co_2(2\text{-}E)_3$ 36
3.4 Mixture of Ill-Defined Species with 2-*Z* and $Co(BF_4)_2$ 41
4. Photochemical characterization of the ligands 43
4.1 Ligand 1 43
4.1.1 ^{1}H NMR measurements 43
4.1.2 UV/vis measurements 45
4.2 Ligand 2 46
4.2.1 ^{1}H NMR measurements 46
4.2.2 UV/vis measurements 47
5. Photochemical characterization of the self-assemblies 48
5.1 Self-assemblies with ligand 1 48
5.1.1 NMR measurements 48
5.1.2 UV/vis measurements 51
5.2 Self-assemblies with ligand 2 53
5.2.1 NMR measurements 53
5.2.2 UV/vis measurements 55
6. Ligand Competition Experiments 57
6.1 Helicate Stability Experiments 57

6.2 Light-Controlled Assembly/Disassembly of Co_2(1-*Z*)$_3$ and Co_2(2-*E*)$_3$ 60
7. Quantum mechanical calculations 62
7.1 Xyz files for the ligands 65
7.1.1 1-*E* (in Co_2(1-*E*)$_3$) 65
7.1.2 1-*Z* (in Co_2(1-*Z*)$_3$) 66
7.1.3 2-*Z* (in Co_2(2-*Z*)$_3$) 67
7.1.4 2-*E* (in Co_2(2-*E*)$_3$) 68
7.1.5 1-*E* (free optimization) 69
7.1.6 1-*Z* (free optimization) 70
7.1.7 2-*E* (free optimization) 71
7.1.8 2-*Z* (free optimization) 72
7.2 Xyz files for the self-assemblies 73
7.2.1 ΔΛ-Co_2(1-*Z*)$_3$ 73
7.2.2 ΔΛ-Co_2(2-*E*)$_3$ 76
7.2.3 ΛΛ-Co_2(1-*Z*)$_3$ 78
7.2.4 ΛΛ-Co_2(2-*E*)$_3$ 81
8. References 84

1. General Methods

NMR spectroscopy

NMR spectra were measured in deuterated solvents (Deutero). As a reference for the NMR spectra, the following solvent signals were used:

Solvent	Degree of deuteration	^{1}H signal (ppm)	^{13}C signal (ppm)
**acetone-d$_6$**	99.8 %	2.05 (quintet)	29.84 (septet)
**acetonitrile-d$_3$**	99.8 %	1.94 ppm (singlet)	1.32 (quartet)<br>118.26 (singlet)
**chloroform-d$_1$**	99.8 %	7.26 (singlet)	77.16 (triplet)
**DMSO-d$_6$**	99.8 %	2.50 (quintet)	39.52 (septet)

The NMR measurements were performed on a Bruker DRX 500 (^{1}H NMR: 500 MHz, ^{13}C NMR: 125 MHz) and a Bruker AV 600 (^{1}H NMR: 600 MHz, ^{13}C NMR: 150 MHz) spectrometer. Paramagnetic NMR spectra were measured using the methods described by Lehr *et al*.[1]

Abbreviations for nuclei assignments are: H^{Trz}/C^{Trz} for the triazole subunit and H^{Py}/C^{Py} for the pyridine subunit. Symmetric NMR signals where the multiplicity and coupling constants cannot be resolved are labeled m_c.

Melting Point

A Melting Point B-560 (Büchi) was used to measure the melting points.

Mass spectrometry

High resolution EI mass spectra were measured on an AccuTOF GCv 4G (Jeol) with an ionization energy of 70 eV. High resolution (ESI) mass spectra were measured on a Thermo Fischer Q Exactive Plus MS, Hybrid Quadrupole-Orbitrap and ASSY-APCI Probe-USI (spray voltage 3-4 eV, temperature 40-50 °C, Harvard syringe pump at a rate of 5-10 µL per minute) by Dynamic Integrated Solutions.

UV/vis spectroscopy

UV/vis spectra were measured on a Lambda 14 UV/vis spectrometer (Perkin-Elmer). Quartz cuvettes with an optical path length of 10 mm were used.

Stationary phases for chromatography

Flash column chromatography purification was performed on an Isolera one (Biotage®) with Puriflash F0040 HP cartridges (Interchim®) with silica gel with a particle diameter of 30 µm. Determination of R_f values was performed with the help of thin layer chromatography on ALUGRAM® Xtra SIL G/UV254 (Macherey Nagel, 0.2 mm particle size).

Light sources

The photophysical properties of the diazocines were determined after irradiation of the samples with custom-built LEDs (SAHLMANN PHOTOCHEMICAL SOLUTIONS) with a wavelength of 385 nm (FWHM = 9 nm, P(opt) = 12 x 340 mW) and 520 nm (FWHM = 33 nm, P(opt) = 16 x 200 mW).

Centrifuge

Centrifugation of the precipitated self-assemblies was performed on a Grant-Bio LMC-3000 low speed benchtop centrifuge.

2. Synthesis

Both diazocine-based ligands were prepared by similar synthetic strategies (Scheme S1) and the synthesis of ligand **2** will be discussed first. Commercially available 4-iodo-1-methyl-2-nitrotoluene was dimerized at low temperatures using *t*-BuOK and bromine following the standard procedure developed by our group.[2] Subsequent reduction of the nitro groups of **3** with $SnCl_2$ hexahydrate yielded the corresponding diamino compound **4**, which was then used in the oxidative azo cyclisation with *m*CPBA (following the procedure from Trauner *et al.*[3]) to obtain diiododiazocine **5**. Diazidodiazocine **6** was prepared using a copper(I)-catalysed Ullmann-type reaction with sodium azide as the nucleophile and then used in a copper-catalyzed azide-alkyne cycloaddition (CuAAC) “click reaction” with 2-ethynylpyridine to yield the final ligand **2**. The synthesis of ligand **1** proceeded via analogous Ullmann and “click” reactions starting from the literature-known *p*-diiododiazocine **7**.[3]

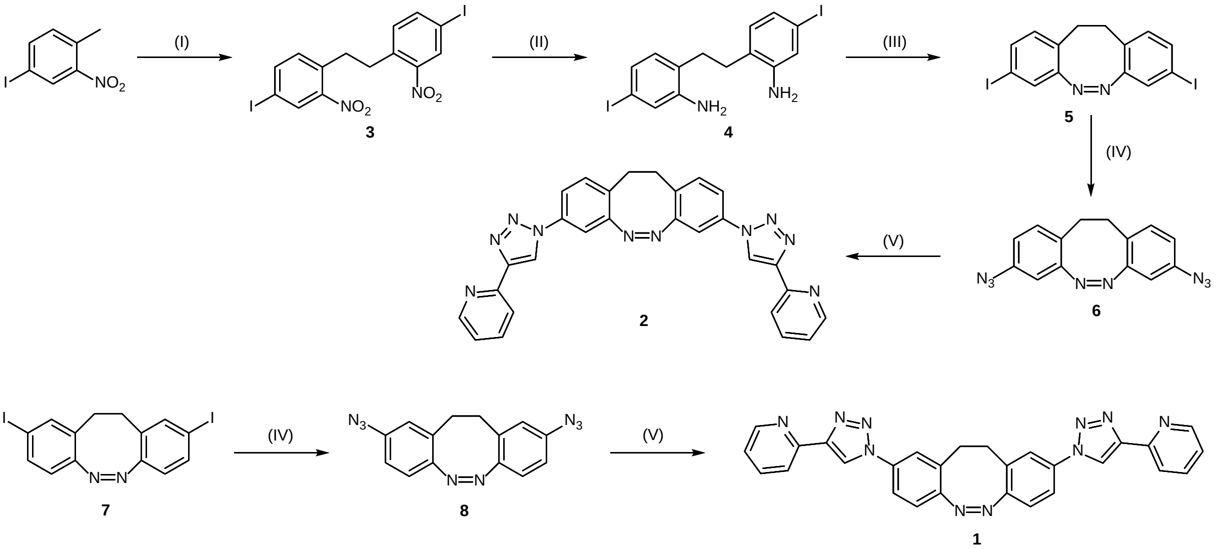

Scheme S1: Synthesis of the photoswitchable diazocine-based ligands **1** and **2**. Reaction conditions: I) *t*-BuOK, Br_2, THF, -15 °C, 10 min, yield not determined; II) $SnCl_2 \cdot 6\ H_2O$, ethyl acetate, 100 °C, 7 h, 48%; III) *m*CPBA, acetic acid, r.t., 20 h, 35%; IV) NaN_3, CuI, *N*,*N*′-dimethylethane-1,2-diamine, EtOH, H_2O, DMSO, 100 °C, 18 h, N_2 atm., compound **6**: 67%, compound **8**: 49%; V) 2-ethynylpyridine, CuI, *N*,*N*-diisopropylethylamine, sodium ascorbate, DCM, 25 °C, 24 h, N_2 atm., compound **1**: 32%, compound **2**: 55%.

2.1 Ligand 2

2.1.1 1,2-Bis(4-iodo-2-nitrophenyl)ethane (3)

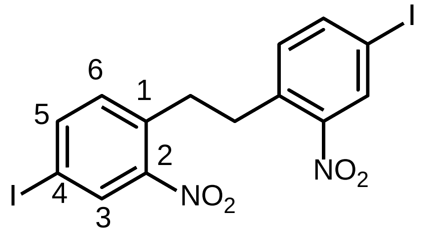

4-Iodo-1-methyl-2-nitrobenzene (4.46 g, 17.0 mmol) was dissolved in 100 mL dry tetrahydrofuran. The solution was stirred and cooled to -15 °C and potassium *tert*-butoxide (2.47 g, 22.0 mmol) was added. After 30 s, bromine (0.87 mL, 17.0 mmol) was added dropwise and the solution was stirred for 15 min while the temperature was kept at −15 °C. The reaction mixture was added to 500 mL of ice water and the precipitate was filtered off after 1 h, washed with 50 mL of cooled acetone and dried *in vacuo* to obtain a yellow solid (**3**, 3.8 g). While the product also contained minor impurities, it was used without further purification in the next step.

R_f: 0.82 (cyclohexane/ethyl acetate, 3:1).

^{1}H NMR (500 MHz, DMSO-d_6, 298 K): δ = 8.26 (d, ^4J = 1.8 Hz, 2H, *H*-3), 8.01 (dd, ^3J = 8.2 Hz, ^4J = 1.8 Hz, 2H, *H*-5), 7.24 (d, ^3J = 8.2 Hz, 2H, *H*-6), 3.08 (s, 4H, C_2H_4) ppm.

^{13}C NMR (125 MHz, DMSO-d_6, 298 K): δ = 149.5 (*C*-2), 141.7 (*C*-5), 134.0 (*C*-1), 133.7 (*C*-6), 132.2 (*C*-3), 92.1 (*C*-4), 32.4 (C_2H_4) ppm.

FT-IR (ATR) $\tilde{\nu}$ = 3082 (w), 1518 (s), 1335 (s), 1130 (w), 1069 (w), 870 (m), 840 (s), 796 (m), 762 (m), 533 (m) cm^{-1}.

MS (EI, 70 eV): *m/z* (%) = 521.86 (3) $[M]^+$, 489.86 (5), 361.95 (10), 261.93 (100), 245.94 (45), 203.94 (35), 176.06 (25), 91.04 (30).

HRMS (EI, 70 eV): *m/z* ($C_{14}H_8I_2N_2O_4$) = calc.: 521.85734, found: 521.85664 ± 1.35 ppm.

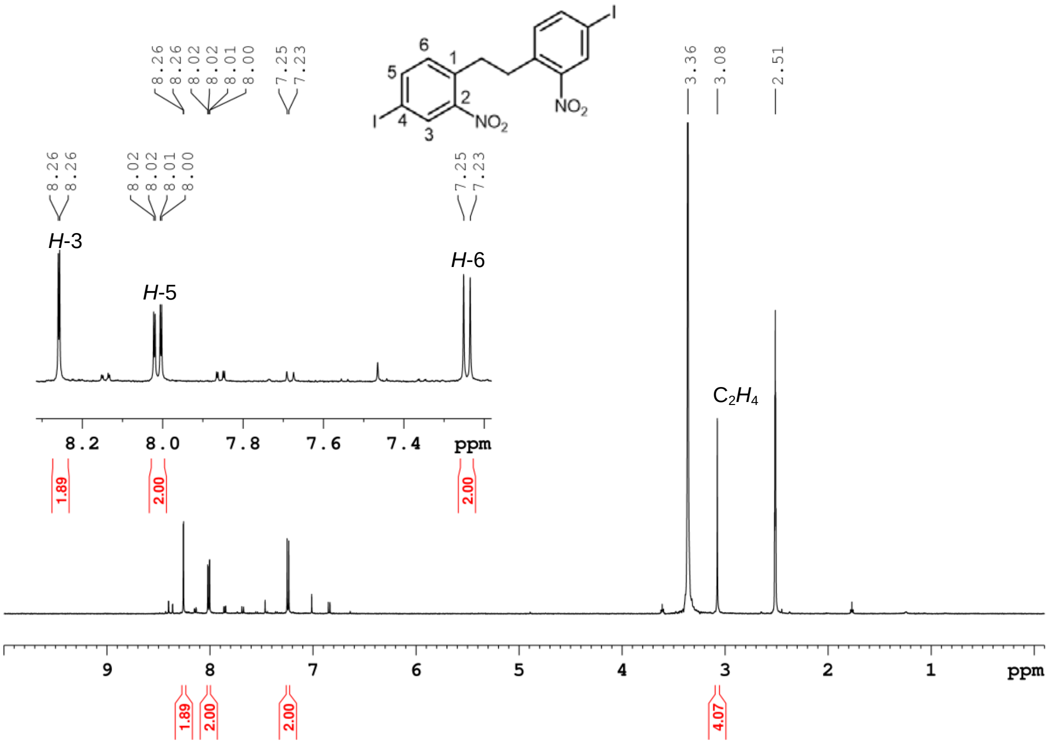

Figure S1: ^{1}H NMR spectrum (500 MHz, DMSO-d6, 298 K) of compound **3**.

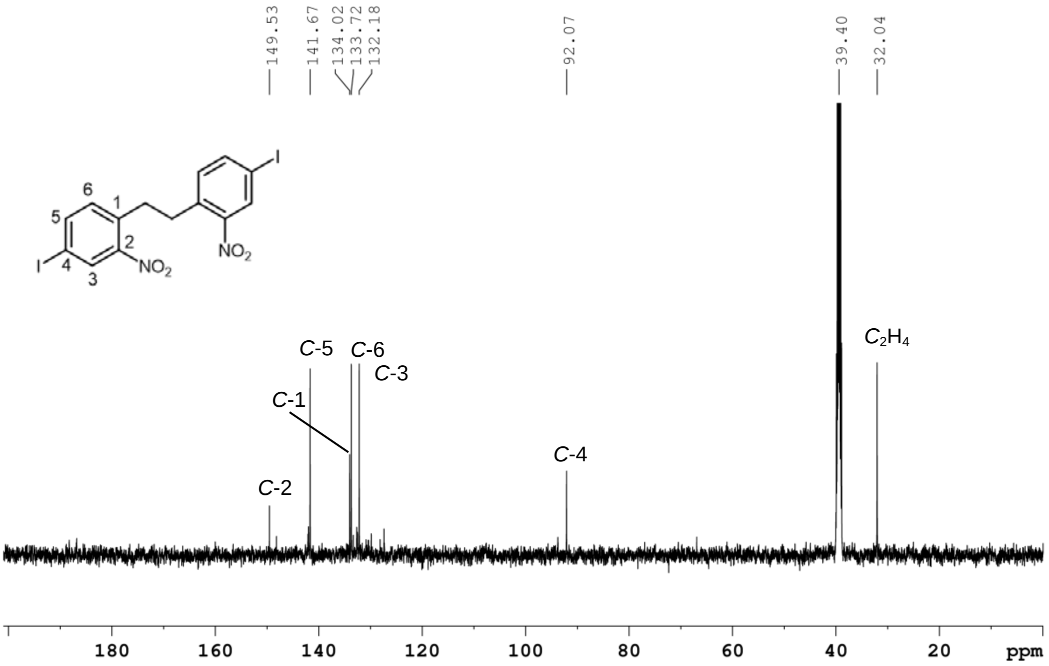

Figure S2: ^{13}C NMR spectrum (125 MHz, DMSO-d6, 298 K) of compound **3**.

2.1.2 1,2-Bis(4-iodo-2-aminophenyl)ethane (4)

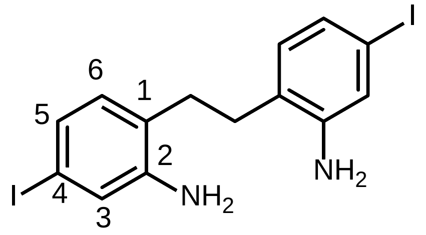

1,2-Bis(4-iodo-2-nitrophenyl)ethane (7.28 g, 13.9 mmol) was dissolved in ethyl acetate (750 mL) and cooled to 0 °C. Tin(II) chloride hexahydrate (25.08 g, 111 mmol) was added portion wise and the solution was heated to 80 °C for 7 h. After cooling to room temperature, the mixture was neutralized with aq. sodium hydroxide solution (40%) and kept at −15 °C for 12 h. The precipitate was filtered off and the filtrate was extracted with ethyl acetate (3 x 250 mL). The combined organic phases were washed with 500 mL sat. sodium chloride solution, dried over magnesium sulfate and the solvent was evaporated *in vacuo*. The crude product was purified by flash column chromatography (cyclohexane/ethyl acetate; 15:1 to 5:1) to obtain a yellow solid (3.1 g, 6.68 mmol, 48%).

Melting point: 156 °C.

R_f: 0.25 (cyclohexane/ethyl acetate, 3:1).

^{1}H NMR (500 MHz, DMSO-d_6, 298 K): δ = 6.98 (d, ^4J = 1.8 Hz, 2H, *H*-3), 6.78 (dd, ^3J = 7.9 Hz, ^4J = 1.8 Hz, 2H, *H*-5), 6.72 (d, ^3J = 7.9 Hz, 2H, *H*-6), 5.11 (s, 4H, N*H*$_2$), 2.57 (s, 4H, C_2H_4) ppm.

^{13}C NMR (125 MHz, DMSO-d_6, 298 K): δ = 148.1 (*C*-2), 130.9 (*C*-6), 124.6 (*C*-1), 124.2 (*C*-5), 122.1 (*C*-3), 91.8 (*C*-4), 28.7 (C_2H_4) ppm.

FT-IR (ATR) $\tilde{\nu}$ = 3340 (w), 1615 (m), 1586 (m), 1564 (m), 1487 (s), 1439 (m), 1402 (m), 1251 (w), 1182 (w), 871 (m), 856 (m), 814 (s), 776 (m), 645 (m_{br}), 565 (s) cm^{-1}.

MS (EI, 70 eV): *m/z* (%) = 464.93 (5), 463.93 $[M]^+$ (20), 232.97 (10), 231.96 (100), 104.05 (20).

HRMS (EI, 70 eV): *m/z* ($C_{14}H_{14}I_2N_2$) = calc.: 463.92463, found: 463.92450 ± 0.29 ppm.

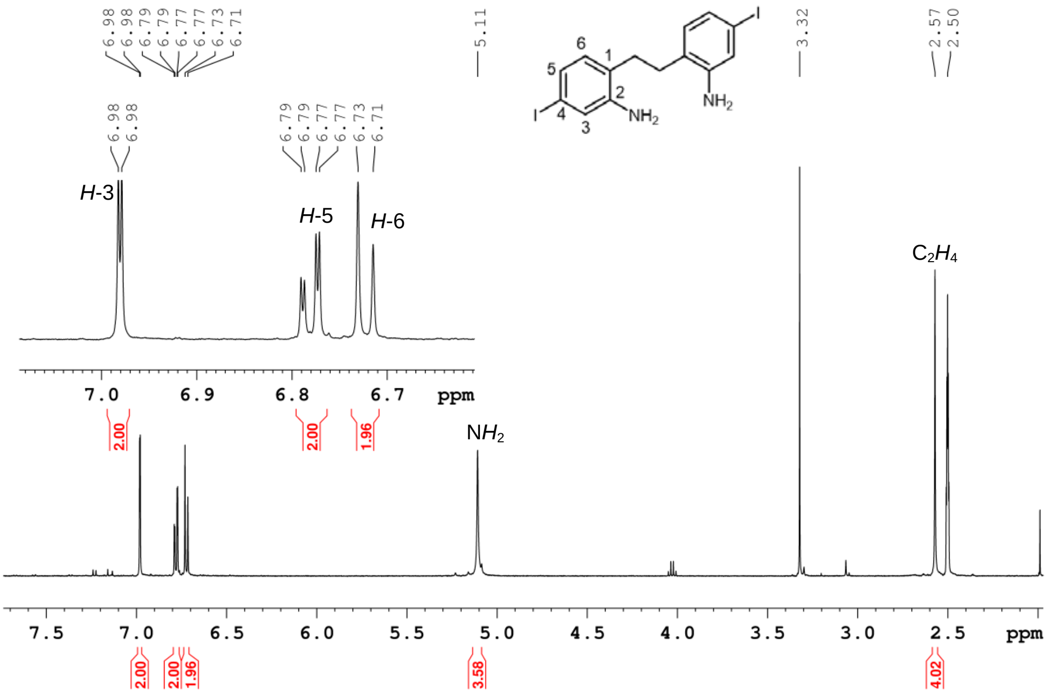

Figure S3: ^{1}H NMR spectrum (500 MHz, DMSO-d$_6$, 298 K) of compound **4**.

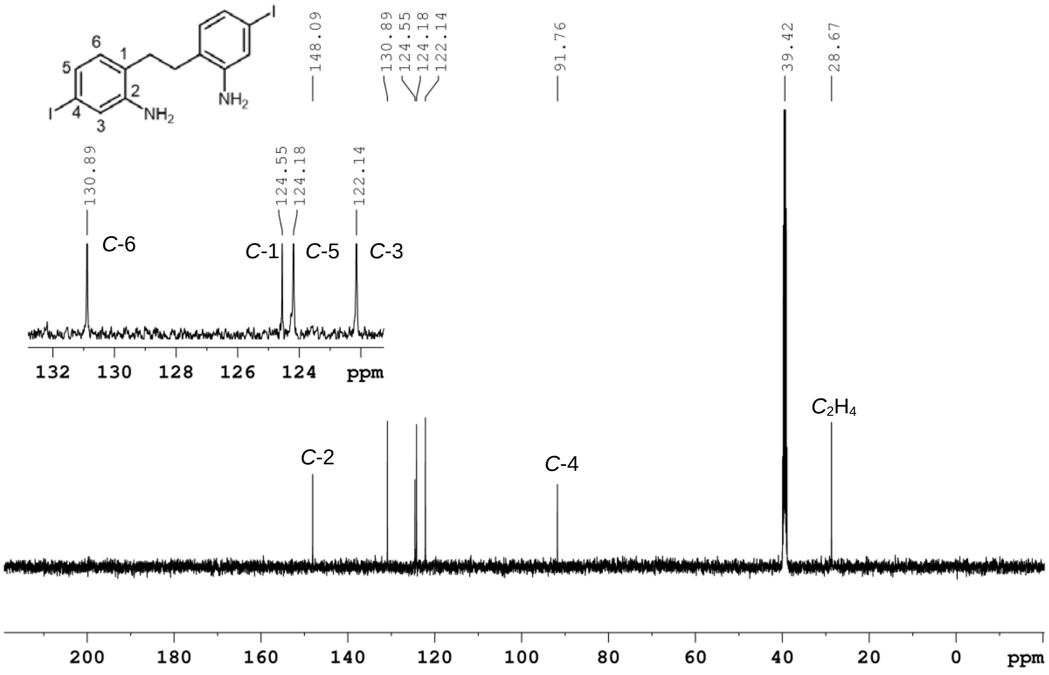

Figure S4: ^{13}C NMR spectrum (125 MHz, DMSO-d$_6$, 298 K) of compound **4**.

2.1.3 3,8-Diiodo-11,12-dihydrodibenzo[*c,g*][1,2]diazocine (5)

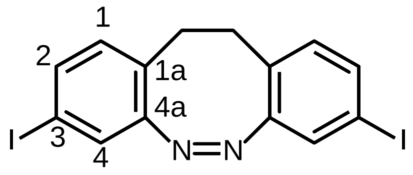

1,2-Bis(4-iodo-2-aminophenyl)ethane (2.00 g, 4.31 mmol) was dissolved in acetic acid (2.00 L). *meta*-Chloroperoxybenzoic acid (1.52 g, 8.83 mmol) was added portionwise over the course of 5 h and the mixture was stirred at room temperature for another 12 h. After removal of the solvent under reduced pressure, the crude product was taken up with dichloromethane (100 mL), washed with saturated aq. sodium carbonate solution (50 mL) and dried over magnesium sulfate. After flash column chromatography (cyclohexane/ethyl acetate; 10:1 to 3:1), the product was obtained as a yellow solid (685 mg, 1.48 mmol, 35%).

Melting point: 141 °C.

R_f: 0.80 (cyclohexane/ethyl acetate, 3:1).

^{1}H NMR (500 MHz, acetone-d_6, 298 K): δ = 7.43 (dd, ^3J = 8.1 Hz, ^4J = 1.8 Hz, 2H, *H*-2), 7.25 (d, ^4J = 1.8 Hz, 2H, *H*-4), 6.92 (d, ^3J = 8.1 Hz, 2H, *H*-1), 2.85 (s, 4H, C_2H_4) ppm.

^{13}C NMR (125 MHz, acetone-d_6, 298 K): δ = 157.4 (*C*-4a), 137.1 (*C*-2), 132.9 (*C*-1), 129.0 (*C*-1a), 127.9 (*C*-4), 91.5 (*C*-3), 31.5 (C_2H_4) ppm.

FT-IR (ATR) $\tilde{\nu}$ = 1685 (w_{br}), 1578 (w), 1469 (w), 1432 (w), 1378 (w), 1305 (w), 1263 (w), 1094 (w), 878 (w), 824 (s), 802 (s), 750 (m), 721 (w), 605 (m), 545 (m) cm^{-1}.

MS (EI, 70 eV): *m/z* (%) = 460.91 (2), 459.91 (20) $[M]^+$, 304.99 (10), 303.98 (20), 179.09 (25), 178.08 (100), 176.07 (30), 152.07 (25), 126.91 (10).

HRMS (EI, 70 eV): *m/z* ($C_{14}H_{10}I_2N_2$) = calc.: 459.89333, found: 459.89311 ± 0.50 ppm.

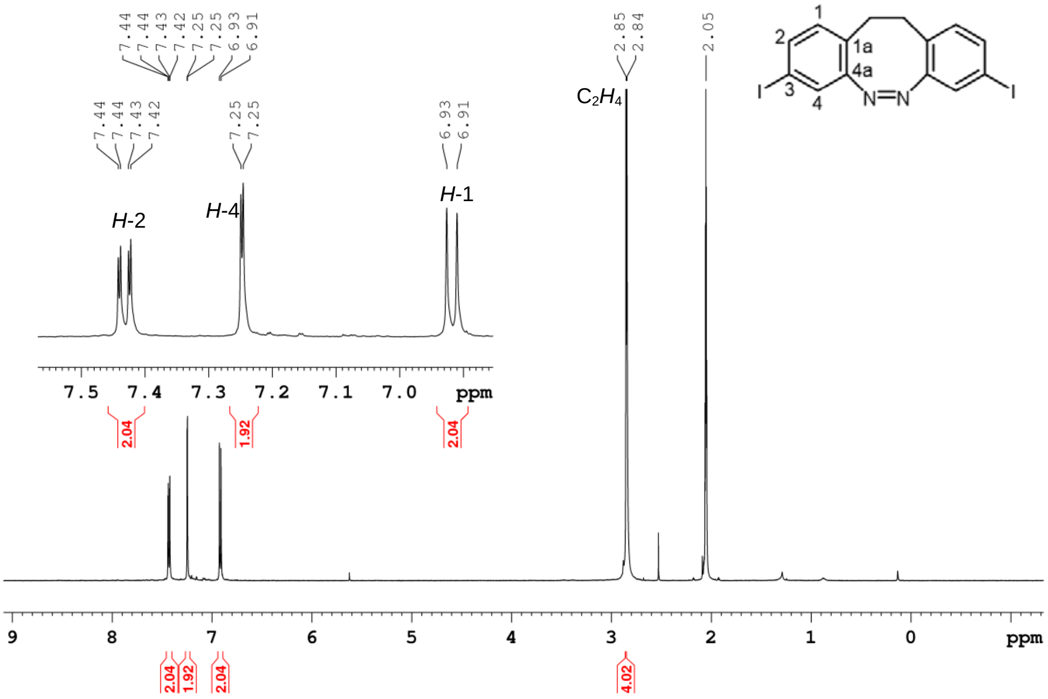

Figure S5: ^{1}H NMR spectrum (500 MHz, acetone-d_6, 298 K) of compound **5**.

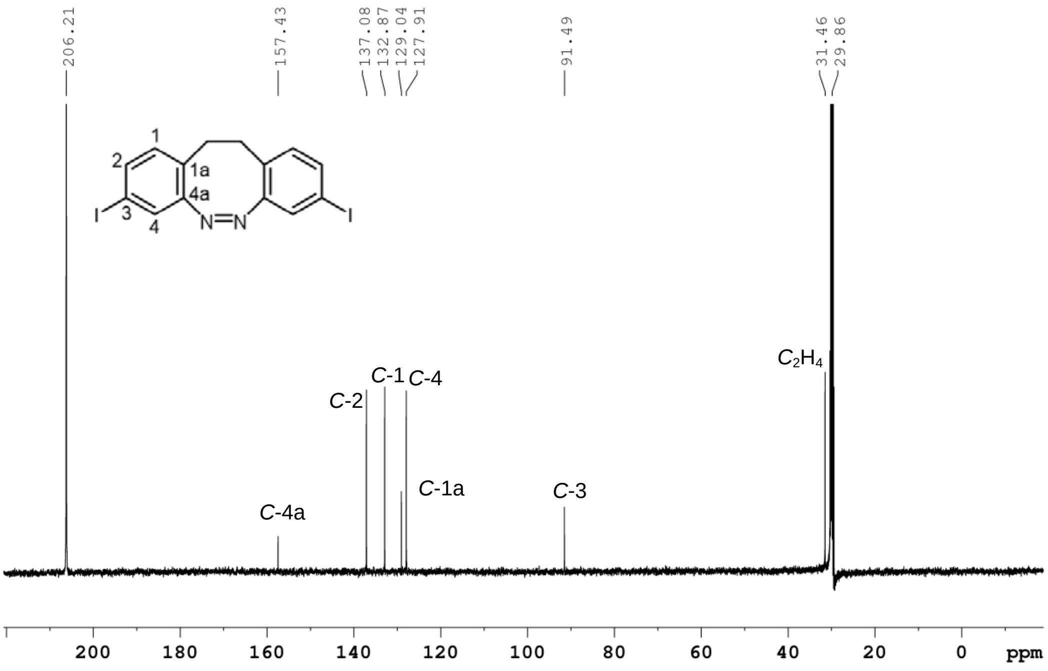

Figure S6: ^{13}C NMR spectrum (125 MHz, acetone-d_6, 298 K) of compound **5**.

2.1.4 3,8-Diazido-11,12-dihydrodibenzo[*c,g*][1,2]diazocine (6)

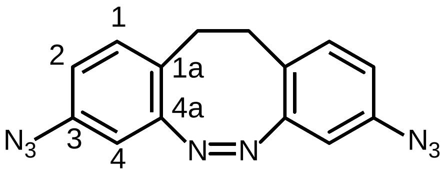

Under a nitrogen atmosphere, 3,8-diiodo-11,12-dihydrodibenzo[*c,g*][1,2]diazocine (660 mg, 1.44 mmol), sodium azide (373 mg, 5.74 mmol), copper(I) iodide (55.0 mg, 287 µmol) and 1,2-dimethylethylenediamine (38.0 mg, 431 µmol) were dissolved in a ethanol/water mixture (3:7, 50 mL) and dimethyl sulfoxide (5 mL) was added. The resulting mixture was heated to 100 °C for 15 h and additional quantities of sodium azide (373 mg, 5.74 mmol), copper(I) iodide (55.0 mg, 287 µmol) and 1,2-dimethylethylenediamine (38.0 mg, 431 µmol) were added. After heating of the mixture to 100 °C for an additional 6 h, the solution was cooled to room temperature, extracted with dichloromethane (3 x 50 mL) and the combined organic layers were washed with sat. sodium chloride solution (100 mL), dried over magnesium sulfate and the solvent was evaporated *in vacuo*. The crude product was purified by flash column chromatography (cyclohexane/ethyl acetate; 10:1 to 3:1) to obtain a yellow solid (**6**, 280 mg, 965 µmol, 67%).

Melting point: 168 °C.

R_f: 0.53 (cyclohexane/ethyl acetate, 3:1).

^{1}H NMR (500 MHz, chloroform-d_1, 298 K): δ = 6.96 (d, ^3J = 8.2 Hz, 2H, *H*-1), 6.71 (dd, ^3J = 8.2 Hz, 2H, ^4J = 2.4 Hz, *H*-2), 6.50 (d, ^4J = 2.4 Hz, 2H, *H*-4), 2.96-2.71 (m, 4H, C_2H_4) ppm.

^{13}C NMR (125 MHz, chloroform-d_1, 298 K): δ = 156.1 (*C*-4a), 138.9 (*C*-3), 131.1 (*C*-1), 124.7 (*C*-1a), 117.8 (*C*-2), 109.4 (*C*-4), 31.1 (C_2H_4) ppm.

FT-IR (ATR) $\tilde{\nu}$ = 2104 (s), 1600 (m), 1571 (m), 1489 (s), 1290 (s), 898 (w), 857 (m), 829 (s), 810 (s), 625 (m), 533 (m) cm^{-1}.

MS (EI, 70 eV): *m/z* (%) = 459.89 (5), 375.00 (10), 291.10 (15), 290.10 (100) $[M]^+$, 206.08 (20), 205.07 (90), 178.07 (90), 151.05 (40).

HRMS (EI, 70 eV): *m/z* ($C_{14}H_{10}N_8$) = calc.: 290.10284, found: 290.10284 ± 0.00 ppm.

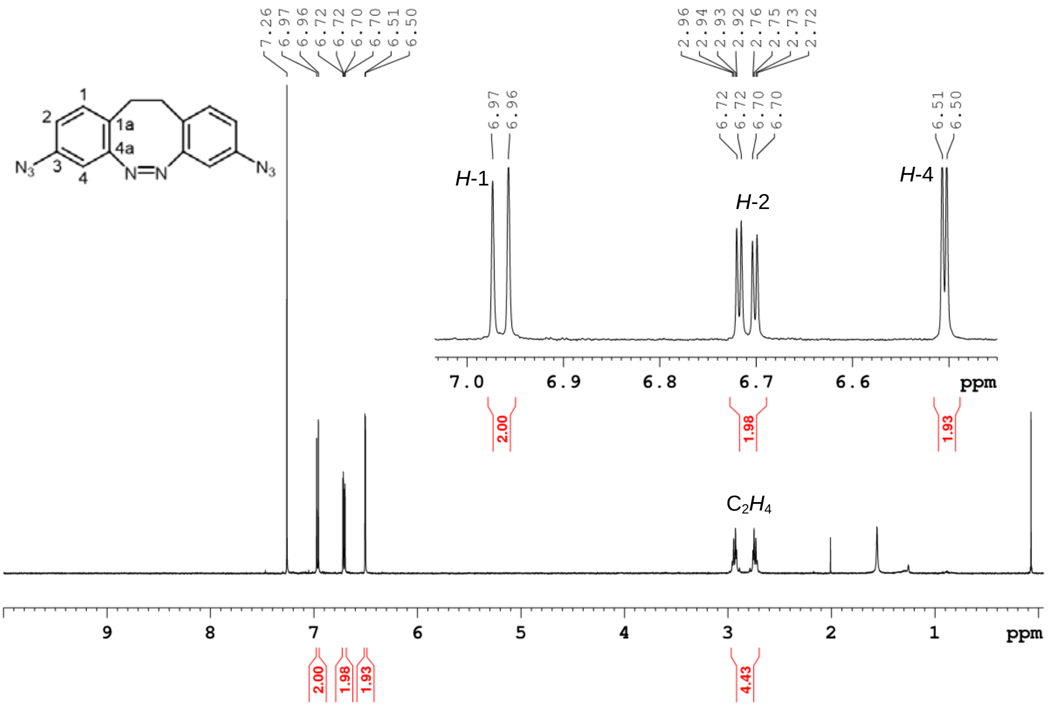

Figure S7: ^{1}H NMR spectrum (500 MHz, chloroform-d$_1$, 298 K) of compound **6**.

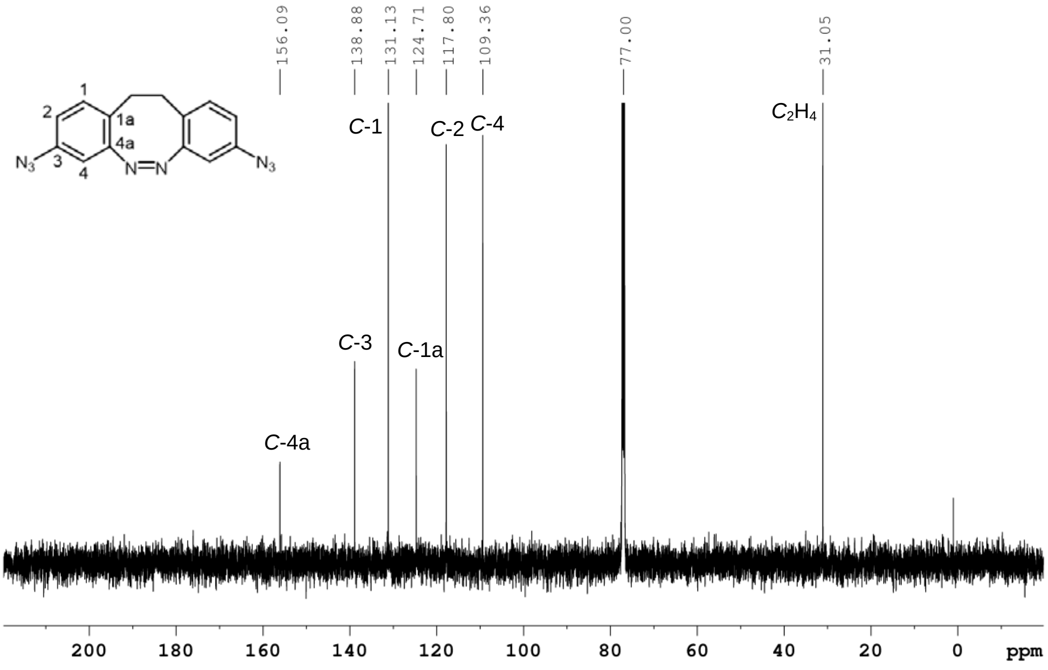

Figure S8: ^{13}C NMR spectrum (125 MHz, chloroform-d$_1$, 298 K) of compound **6**.

2.1.5 3,8-Bis(4-pyridin-2-yl)-1*H*-1,2,3-triazol-1-yl)-11,12-dihydrodibenzo[*c,g*][1,2] diazocine (2)

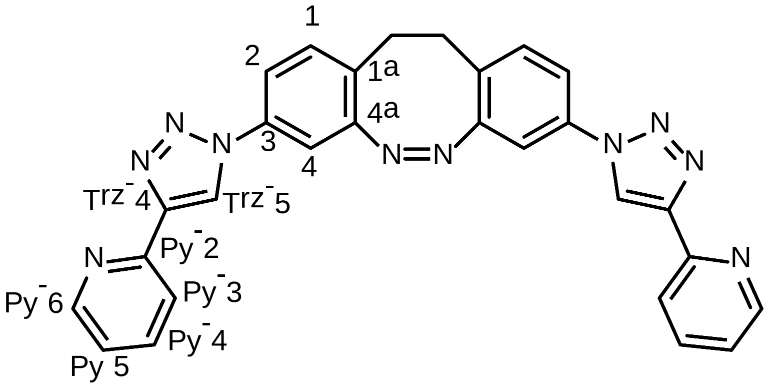

3,8-Diazido-11,12-dihydrodibenzo[*c*,*g*][1,2]diazocine (**6**, 213 mg, 734 µmol), copper(I) iodide (560 mg, 2.94 mmol), 2-ethynylpyridine (454 mg, 4.40 mmol), *N*,*N*-diisopropylethylamine (50 µL, 287 µmol) and sodium ascorbate (200 mg, 1.01 mmol) were dissolved under a nitrogen atmosphere in dry and degassed dichloromethane (100 mL). The reaction mixture was stirred at room temperature for 22 h, saturated aq. ethylenediaminetetraacetic acid (pH 8, 200 mL) was added and the mixture stirred vigorously for 3 h at room temperature. The reaction mixture was extracted with dichloromethane (3 x 100 mL), and the combined organic phases washed with saturated aq. ethylenediaminetetraacetic acid (pH 8, 100 mL), dried over magnesium sulfate and the solvent was evaporated *in vacuo*. The crude product was purified by flash column chromatography (cyclohexane/ethyl acetate; 3:1 to 1:1) to obtain a yellow solid (**2**, 200 mg, 403 µmol, 55%).

Melting point: 285 °C.

R*f*: 0.41 (cyclohexane/ethyl acetate, 3:1).

^{1}H NMR (500 MHz, chloroform-d$_1$, 298 K): δ = 8.70 (s, 2H, H^{Trz}-5), 8.59 (m$_c$, 2H, H^{Py}-6), 8.25-8.23 (m, 2H, H^{Py}-3), 7.84 (td, ^3J = 7.8 Hz, ^4J = 1.7 Hz, 2H, H^{Py}-4), 7.55 (dd, ^3J = 8.3 Hz, ^4J = 2.3 Hz, 2H, *H*-2), 7.40 (d, ^4J = 2.3 Hz, 2H, *H*-4), 7.29 (m$_c$, 2H, H^{Py}-5), 7.22 (d, ^3J = 8.3 Hz, 2H, *H*-1), 3.14-2.90 (m, 4H, C_2H_4) ppm.

^{13}C NMR (125 MHz, chloroform-d$_1$, 298 K): δ = 155.9 (*C*-4a), 149.2 (C^{Py}-2), 148.7 (C^{Py}-6), 148.2 (C^{Trz}-4), 137.8 (C^{Py}-4), 135.7 (*C*-3), 131.4 (*C*-1), 128.6 (*C*-1a), 123.3 (C^{Py}-5), 120.7 (C^{Py}-3), 120.4 (C^{Trz}-5), 119.1 (*C*-2), 110.9 (*C*-4), 31.2 (C_2H_4) ppm.

FT-IR (ATR) $\tilde{\nu}$ = 1600 (m), 1503 (m), 1471 (m), 1393 (w), 1240 (w), 1028 (s), 877 (w), 828 (m), 778 (s), 741 (m), 715 (w) cm^{-1}.

MS (ESI): *m/z* (%) = 587.09 (45), 497.19 (95) [M]$^+$, 386.03 (35), 297.98 (100).

HRMS (ESI): *m/z* ($C_{28}H_{21}N_{10}$) = calc.: 497.19452, found: 497.19416 ± 0.72 ppm.

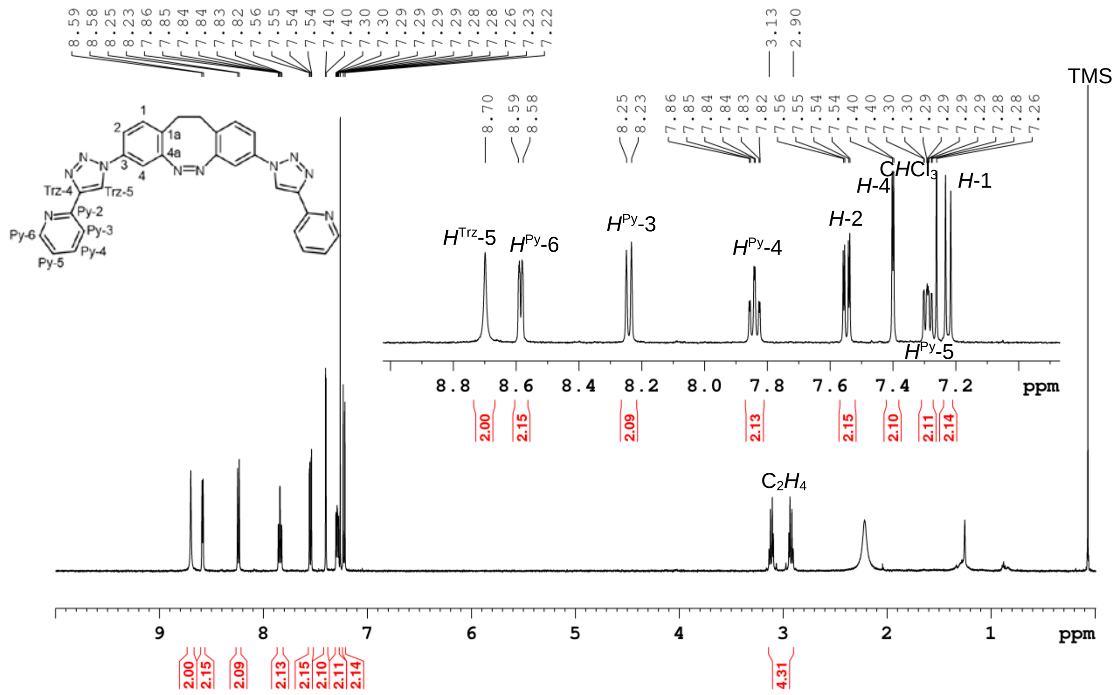

Figure S9: ^{1}H NMR spectrum (500 MHz, chloroform-d$_1$, 298 K) of ligand **2**.

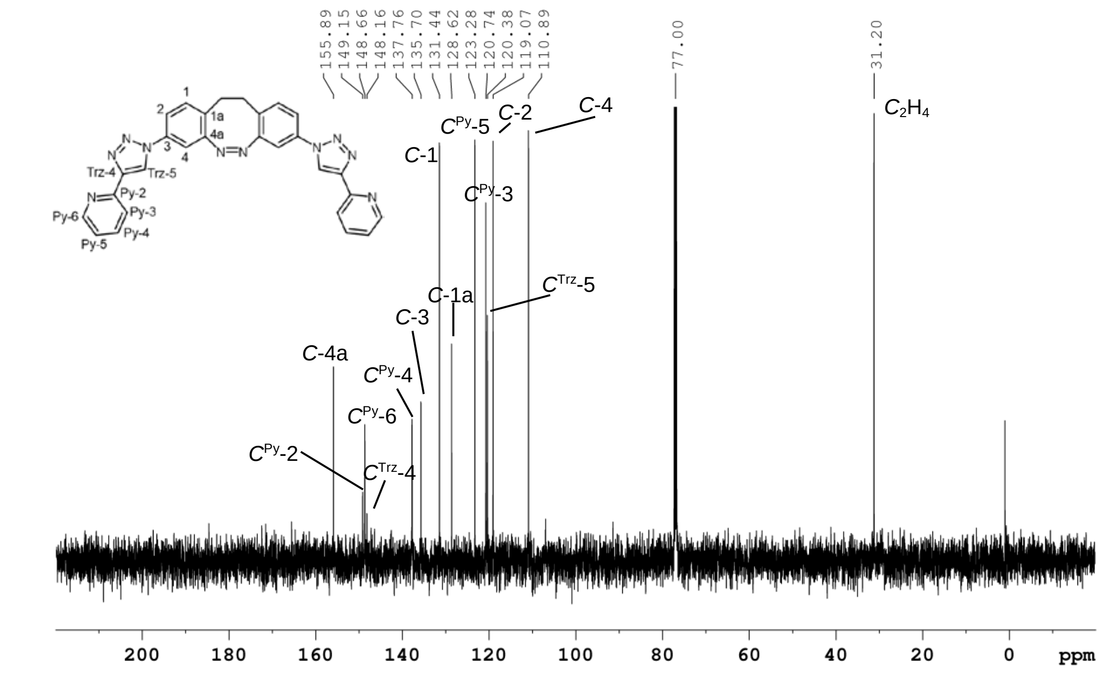

Figure S10: ^{13}C NMR spectrum (125 MHz, chloroform-d$_1$, 298 K) of ligand **2**.

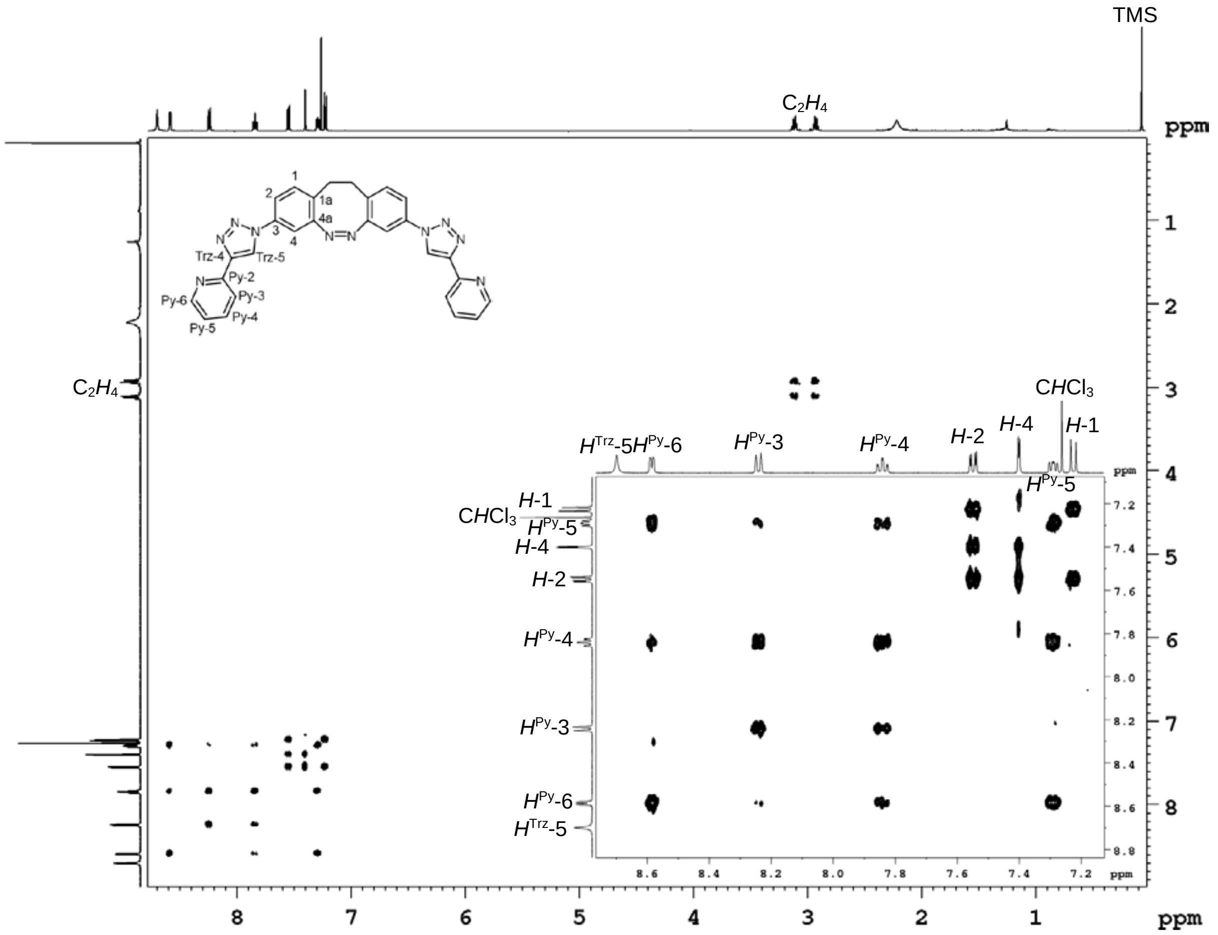

Figure S11: ^{1}H-^{1}H COSY NMR spectrum (500 MHz, chloroform-d_1, 298 K) of ligand **2**.

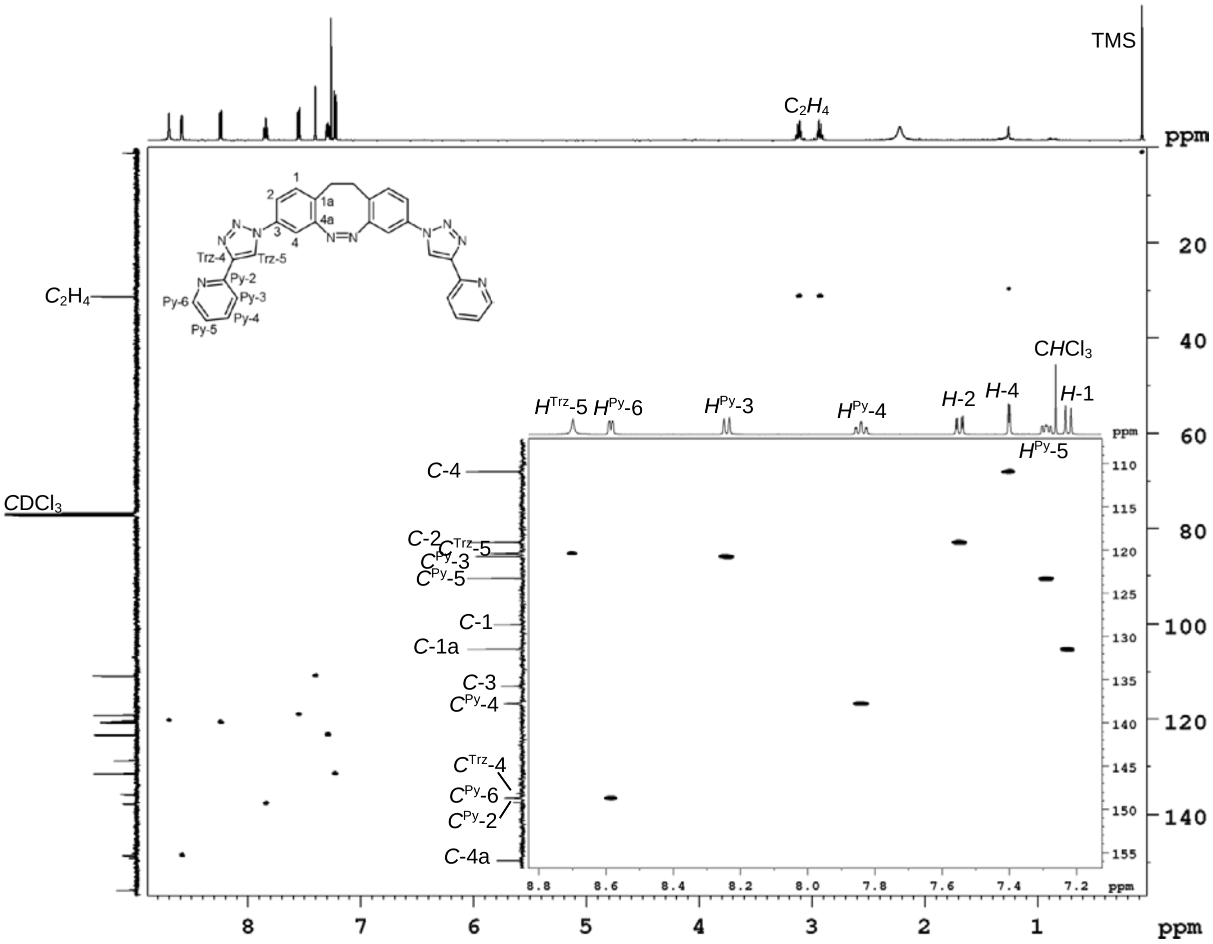

Figure S12: ^{1}H-^{13}C HSQC NMR spectrum (500 MHz/125 MHz, chloroform-d$_1$, 298 K) of ligand **2**.

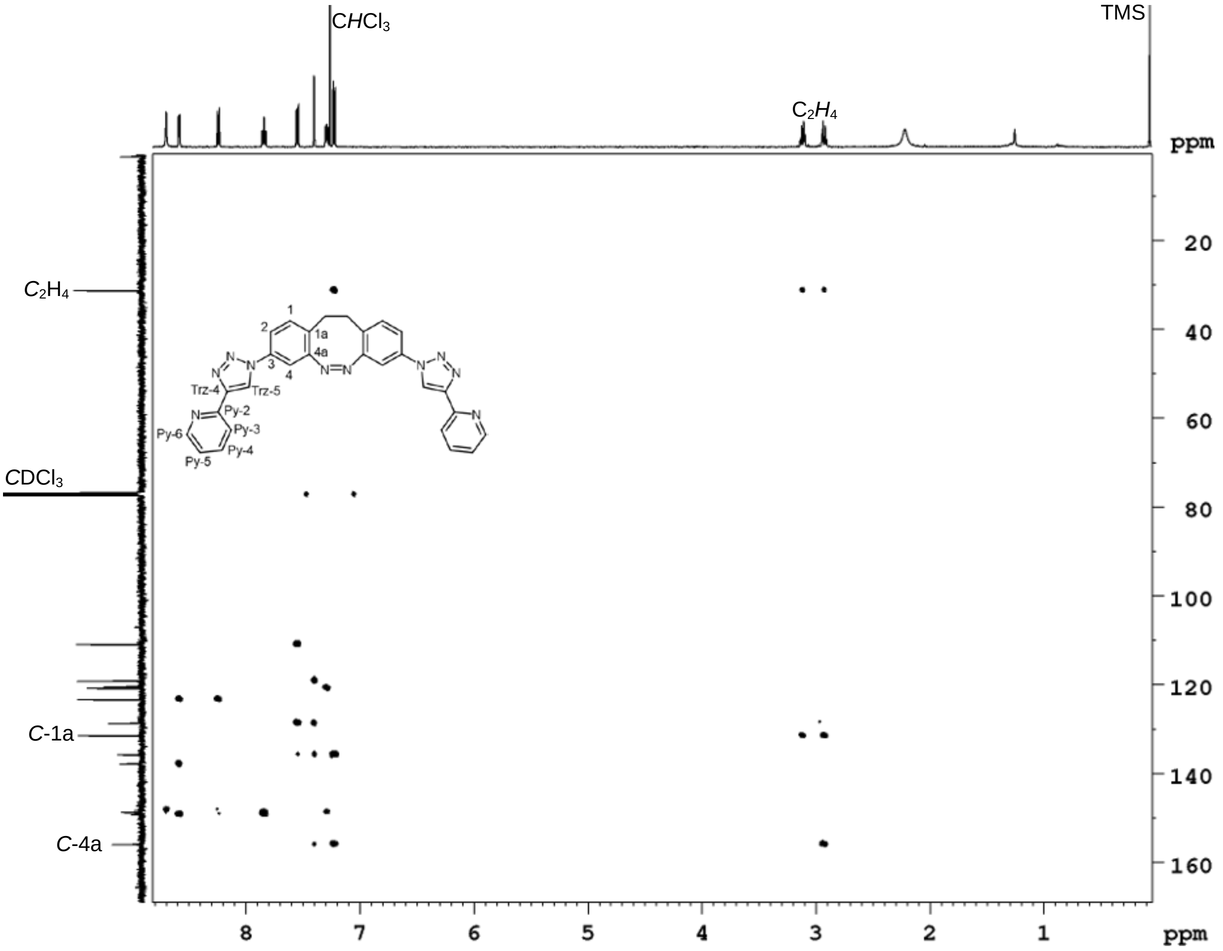

Figure S13: ^{1}H-^{13}C HMBC NMR spectrum (500 MHz/125 MHz, chloroform-d_1, 298 K) of ligand **2**.

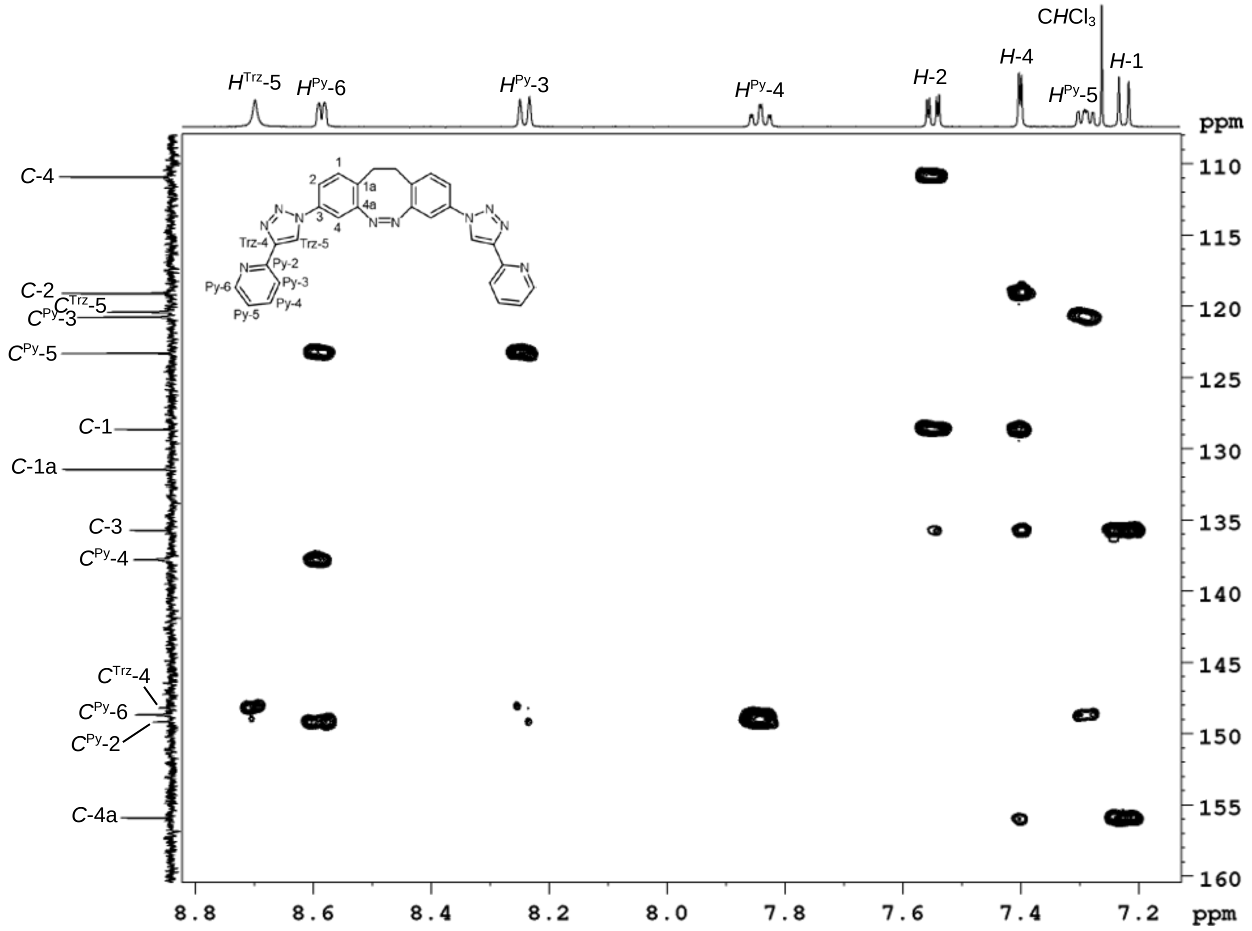

Figure S13.1: Zoomed region of the ^{1}H-^{13}C HMBC NMR spectrum (500 MHz/125 MHz, chloroform-d_1, 298 K) of ligand **2**.

2.2 Ligand 1

2.2.1 2,9-Diiodo-11,12-dihydrodibenzo[*c,g*][1,2]diazocine (7)

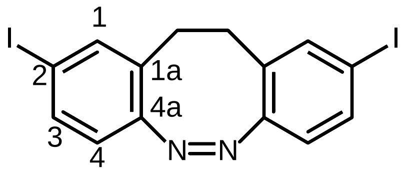

1,2-Bis(5-iodo-2-aminophenyl)ethane (7.34 g, 15.8 mmol) was dissolved in acetic acid (2.00 L). *meta*-Chloroperoxybenzoic acid (5.46 g, 31.6 mmol) was added portionwise over the course of 4 h and the mixture was stirred at room temperature for another 15 h. After removal of the solvent *in vacuo*, the crude product was taken up with dichloromethane (250 mL), washed with saturated aq. sodium carbonate solution (200 mL) and dried over magnesium sulfate. After flash column chromatography (cyclohexane/ethyl acetate; 10:1 to 3:1), the product was obtained as a yellow solid (**7**, 3.60 g, 7.83 mmol, 49%).

Melting point: 192 °C.

R_f: 0.78 (cyclohexane/ethyl acetate, 3:1).

^{1}H NMR (500 MHz, DMSO-d_6, 298 K): δ = 7.55 (dd, ^3J = 8.2 Hz, ^4J = 1.8 Hz, 2H, *H*-3), 7.53 (d, ^4J = 1.8 Hz, 2H, *H*-1), 6.70 (d, ^3J = 8.2 Hz, 2H, *H*-4), 2.83-2.73 (m, 4H, C_2H_4) ppm.

^{13}C NMR (125 MHz, DMSO-d_6, 298 K): δ = 154.4 (*C*-4a), 138.0 (*C*-1), 135.5 (*C*-3), 130.3 (*C*-1a), 120.6 (*C*-4), 92.8 (*C*-2), 30.0 (C_2H_4) ppm.

FT-IR (ATR) $\tilde{\nu}$ = 1509 (w), 1460 (m), 1383 (w), 1155 (w), 1099 (w), 892 (m), 878 (m), 816 (m), 802 (s), 676 (m), 513 (m) cm^{-1}.

MS (ESI): *m/z* (%) = 460.90 (10) $[M]^+$, 416.92 (27), 267.01 (70), 181.08 (100).

HRMS (ESI): *m/z* ($C_{14}H_{11}N_2I_2$) = calc.: 460.90061, found: 460.90031 ± 0.65 ppm.

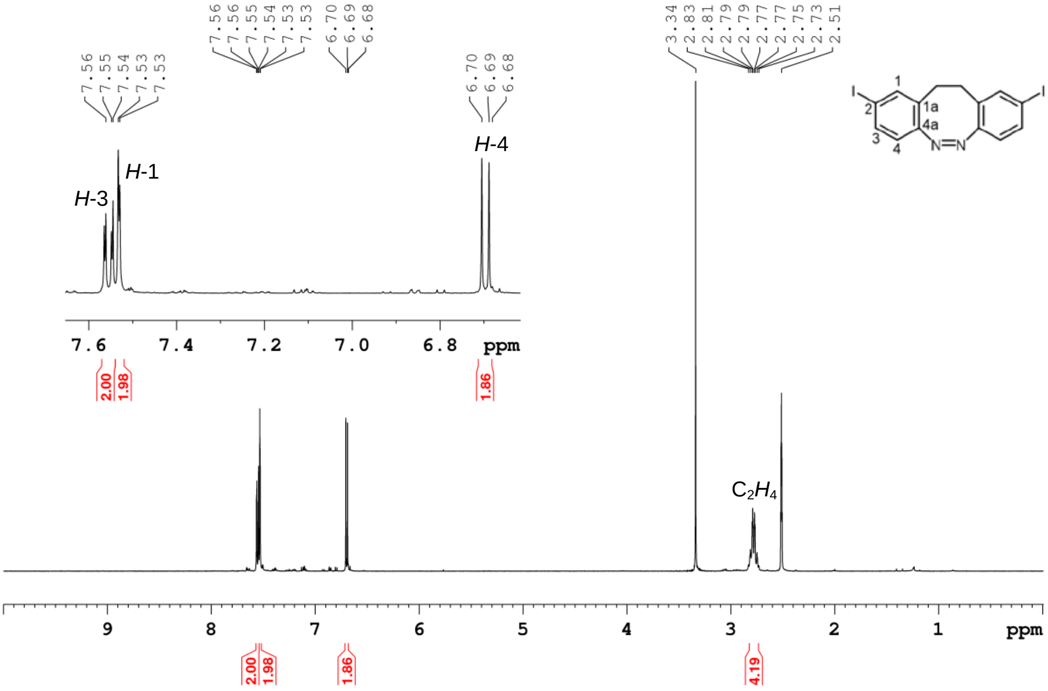

Figure S14: ^{1}H NMR spectrum (500 MHz, DMSO-d_6, 298 K) of compound **7**.

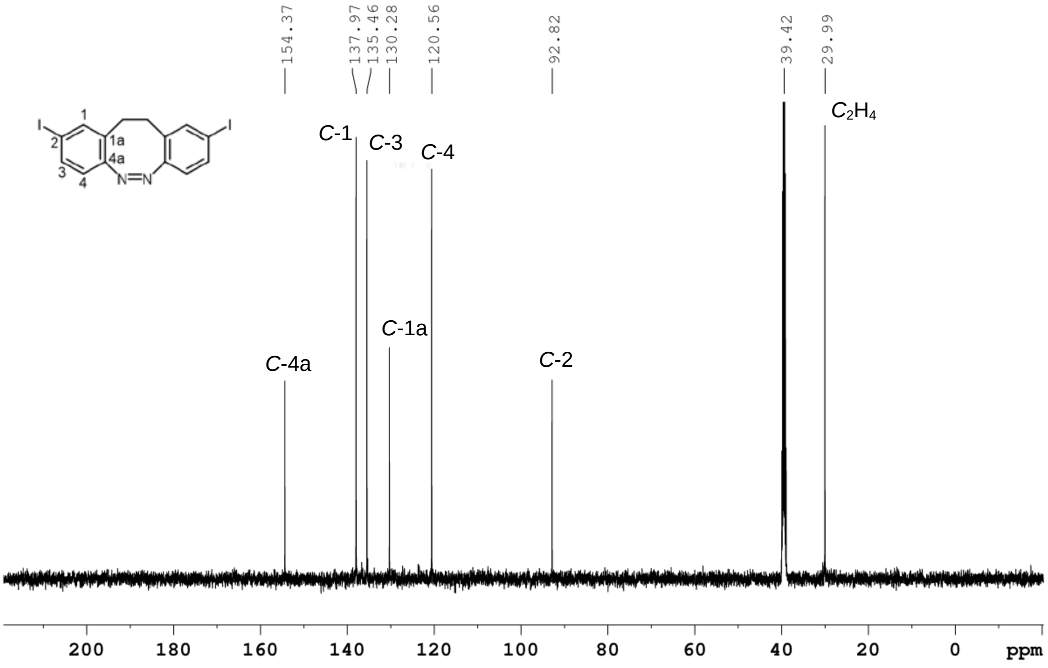

Figure S15: ^{13}C NMR spectrum (125 MHz, DMSO-d_6, 298 K) of compound **7**.

2.2.2 2,9-Diazido-11,12-dihydrodibenzo[*c,g*][1,2]diazocine (8)

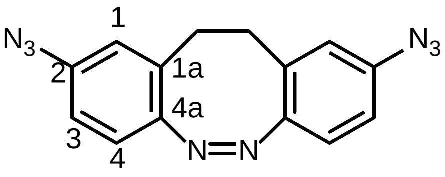

2,9-Diiodo-11,12-dihydrodibenzo[*c,g*][1,2]diazocine (**7**, 1.17 g, 2.54 mmol), sodium azide (659 mg, 10.1 mmol), copper(I) iodide (97.0 mg, 507 µmol) and 1,2-dimethylethylenediamine (67.0 mg, 761 µmol) were dissolved under a nitrogen atmosphere in a ethanol/water mixture (3:7, 50 mL) and dimethyl sulfoxide (10 mL) was added. The resulting mixture was heated to 100 °C for 15 h and additional quantities of sodium azide (373 mg, 5.74 mmol), copper(I) iodide (55.0 mg, 287 µmol) and 1,2-dimethylethylenediamine (38.0 mg, 431 µmol) were added. After heating the mixture to 100 °C for an additional 6 h, the solution was cooled to room temperature, extracted with dichloromethane (3 x 100 mL) and the combined organic layers were washed with sat. sodium chloride solution (200 mL), dried over magnesium sulfate and the solvent was removed *in vacuo*. The crude product was purified by flash column chromatography (cyclohexane/ethyl acetate; 10:1 to 3:1) to obtain a yellow solid (**8**, 500 mg, 1.72 mmol, 68%).

Melting point: 132 °C.

R_f: 0.52 (cyclohexane/ethyl acetate, 3:1).

^{1}H NMR (500 MHz, DMSO-d_6, 298 K): δ = 6.95 (dd, ^3J = 8.4 Hz, ^4J = 2.2 Hz, 2H, *H*-3), 6.92 (d, ^3J = 8.4 Hz, 2H, *H*-4), 6.90 (d, ^4J = 2.2 Hz, 2H, *H*-1), 2.87-2.80 (m, 4H, C_2H_4) ppm.

^{13}C NMR (125 MHz, DMSO-d_6, 298 K): δ = 152.0 (*C*-4), 138.0 (*C*-1a), 130.0 (*C*-2), 120.5 (*C*-4), 119.9 (*C*-1), 117.5 (*C*-3), 30.4 (C_2H_4) ppm.

FT-IR (ATR) $\tilde{\nu}$ = 2103 (s_{br}), 1597 (w), 1570 (w), 1479 (m), 1427 (w), 1298 (s), 1280 (s), 1266 (s), 1177 (w), 891 (m), 824 (m), 804 (m), 681 (m), 533 (m) cm^{-1}.

MS (EI, 70 eV): *m/z* (%) = 375.00 (70), 290.10 (7) $[M]^+$, 220.09 (100), 190.07 (70), 165.07 (80).

HRMS (EI 70 eV): *m/z* ($C_{14}H_{10}N_8$) = calc.: 290.10271, found: 290.10284 ± 0.13 ppm.

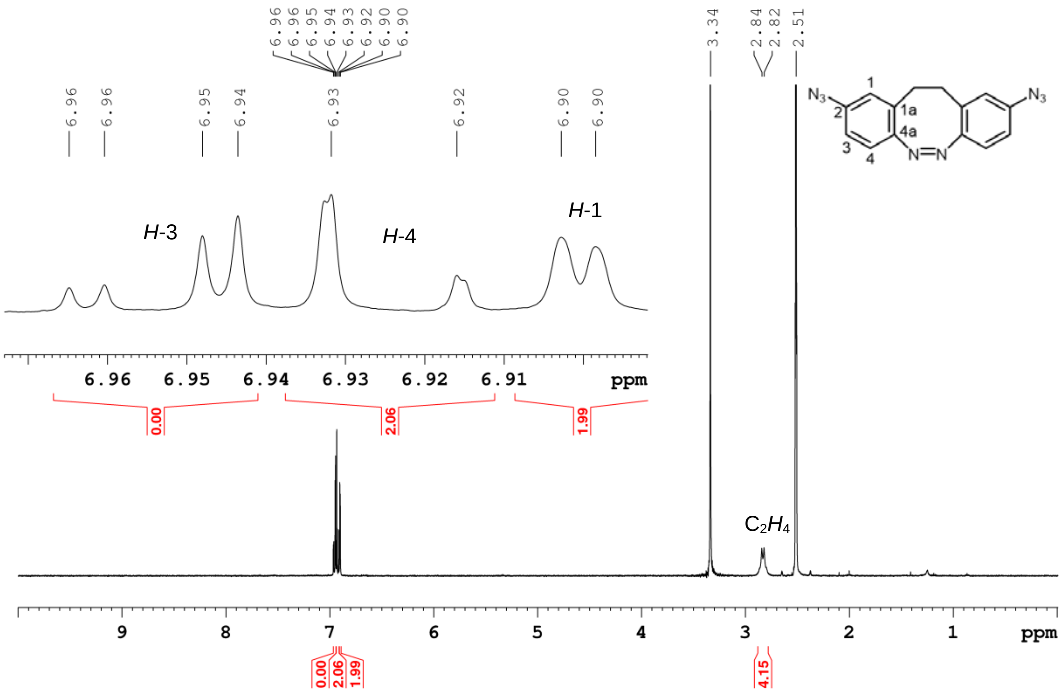

Figure S16: ^{1}H NMR spectrum (500 MHz, DMSO-d$_6$, 298 K) of compound **8**.

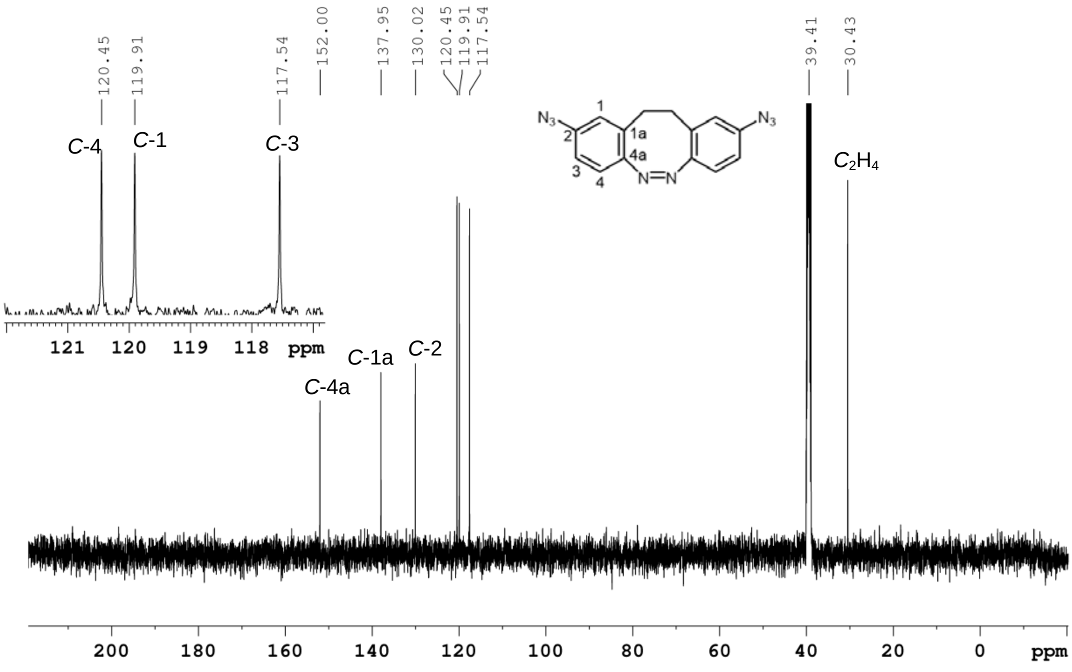

Figure S17: ^{13}C NMR spectrum (125 MHz, DMSO-d$_6$, 298 K) of compound **8**.

2.2.3 2,9-Bis(4-pyridin-2-yl)-1*H*-1,2,3-triazol-1-yl)-11,12-dihydrodibenzo[*c,g*][1,2] diazocine (1)

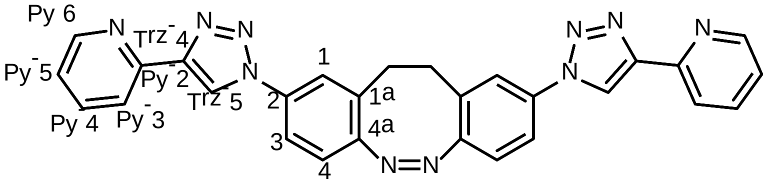

2,9-Diazido-11,12-dihydrodibenzo[*c,g*][1,2]diazocine (**8**, 500 mg, 1.72 mmol), copper(I) iodide (1.31 g, 6.89 mmol), 2-ethynylpyridine (711 mg, 6.89 mmol), *N*,*N*-diisopropylethylamine (250 µL, 2.55 mmol) and sodium ascorbate (360 mg, 1.82 mmol) were dissolved under a nitrogen atmosphere in dry and degassed dichloromethane (200 mL). The reaction mixture was stirred at room temperature for 6 h, followed by addition of additional copper(I) iodide (1.31 g, 6.89 mmol), 2-ethynylpyridine (711 mg, 6.89 mmol), *N*,*N*-diisopropylethylamine (250 µL, 2.55 mmol) and sodium ascorbate (360 mg, 1.82 mmol) and further stirring for 12 h. Saturated aq. ethylenediaminetetraacetic acid (pH 8, 300 mL) was added and the mixture stirred vigorously for 3 h at room temperature. The reaction mixture was extracted with dichloromethane (3 x 100 mL), and the combined organic phases washed with saturated aq. ethylenediaminetetraacetic acid (pH 8, 300 mL), dried over magnesium sulfate and the solvent evaporated *in vacuo*. The crude product was purified by flash column chromatography (cyclohexane/ethyl acetate; 3:1 to 1:1) to obtain a yellow solid (**1**, 277 mg, 558 µmol, 32%).

Melting point: 236 °C.

R*f*: 0.44 (cyclohexane/ethyl acetate, 3:1).

^{1}H NMR (600 MHz, chloroform-d_1, 298 K): δ = 8.62-8.56 (m, 4H, H^{Trz}-5, H^{py}-6), 8.21 (d, ^{3}J = 7.7 Hz, 2H, H^{Py}-3), 7.81 (t, ^{3}J = 7.5 Hz, 2H, H^{Py}-4), 7.62 (dd, ^{3}J = 8.5 Hz, ^{4}J = 1.8 Hz, 2H, *H*-3), 7.59 (d, ^{4}J = 1.8 Hz, 2H, *H*-1), 7.26 (m_c, 2H, H^{Py}-5), 7.08 (d, ^{3}J = 8.5 Hz, 2H, *H*-4), 3.20-2.96 (m, 4H, C_2H_4) ppm.

^{13}C NMR (151 MHz, chloroform-d_1, 298 K): δ = 155.2 (*C*-4a), 149.5 (C^{Py}-2), 149.1 (C^{Py}-6), 148.7 (C^{Trz}-4), 137.3 (C^{Py}-4), 135.8 (*C*-2), 129.9 (*C*-1a), 123.2 (C^{Py}-5), 121.7 (*C*-1), 120.6 (C^{Py}-3), 120.6 (C-4), 120.1 (C^{Trz}-5), 118.9 (*C*-3), 31.6 (C_2H_4) ppm.

FT-IR (ATR) $\tilde{\nu}$ = 1736 (w_{br}), 1603 (m), 1494 (m), 1427 (m), 1400 (w), 1363 (w), 1231 (m), 1025 (s), 886 (m), 773 (s), 746 (m), 714 (m) cm^{-1}.

MS (EI, 70 eV): *m/z* (%) = 496.18 (30) $[M]^+$, 468.18 (29), 439.16 (35), 412.16 (45), 411.16 (100), 333.12 (35), 295.12 (50), 293.10 (95), 292.10 (40), 267.10 (45), 178.07 (48), 177.07 (50), 176.06 (90), 152.06 (46).

HRMS (EI, 70 eV): *m/z* ($C_{28}H_{20}N_{10}$) = calc.: 496.18724, found: 496.18640 ± 1.69 ppm.

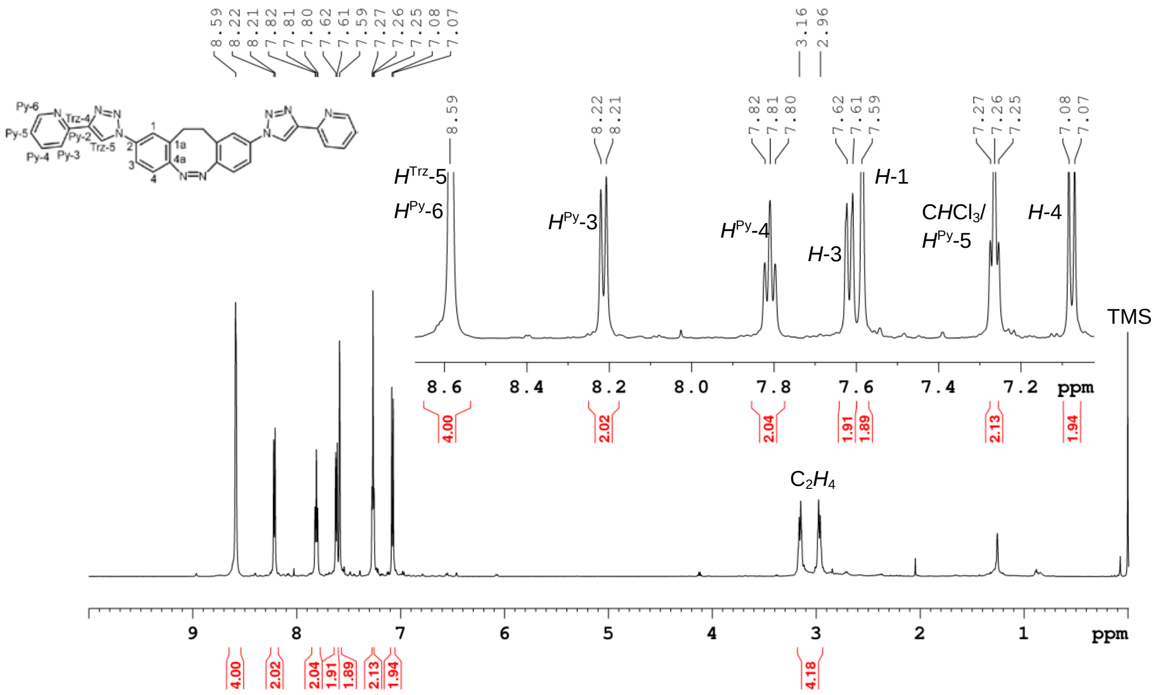

Figure S18: ^{1}H NMR spectrum (600 MHz, chloroform-d$_1$, 298 K) of ligand **1**.

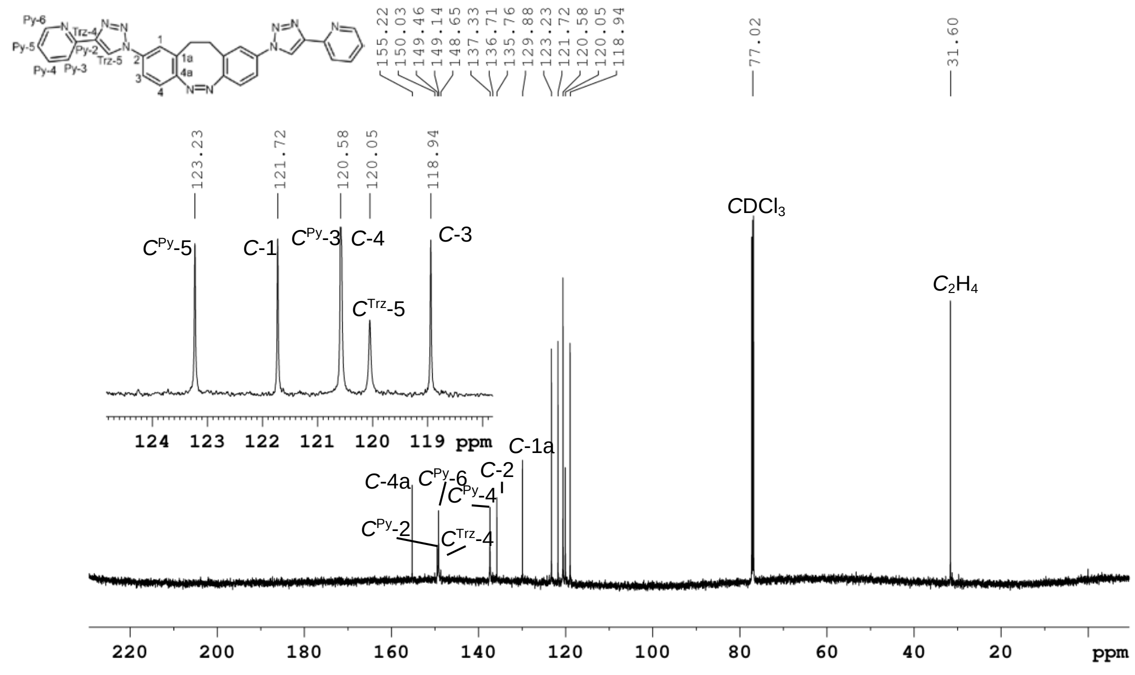

Figure S19: ^{13}C NMR spectrum (151 MHz, chloroform-d$_1$, 298 K) of ligand **1**.

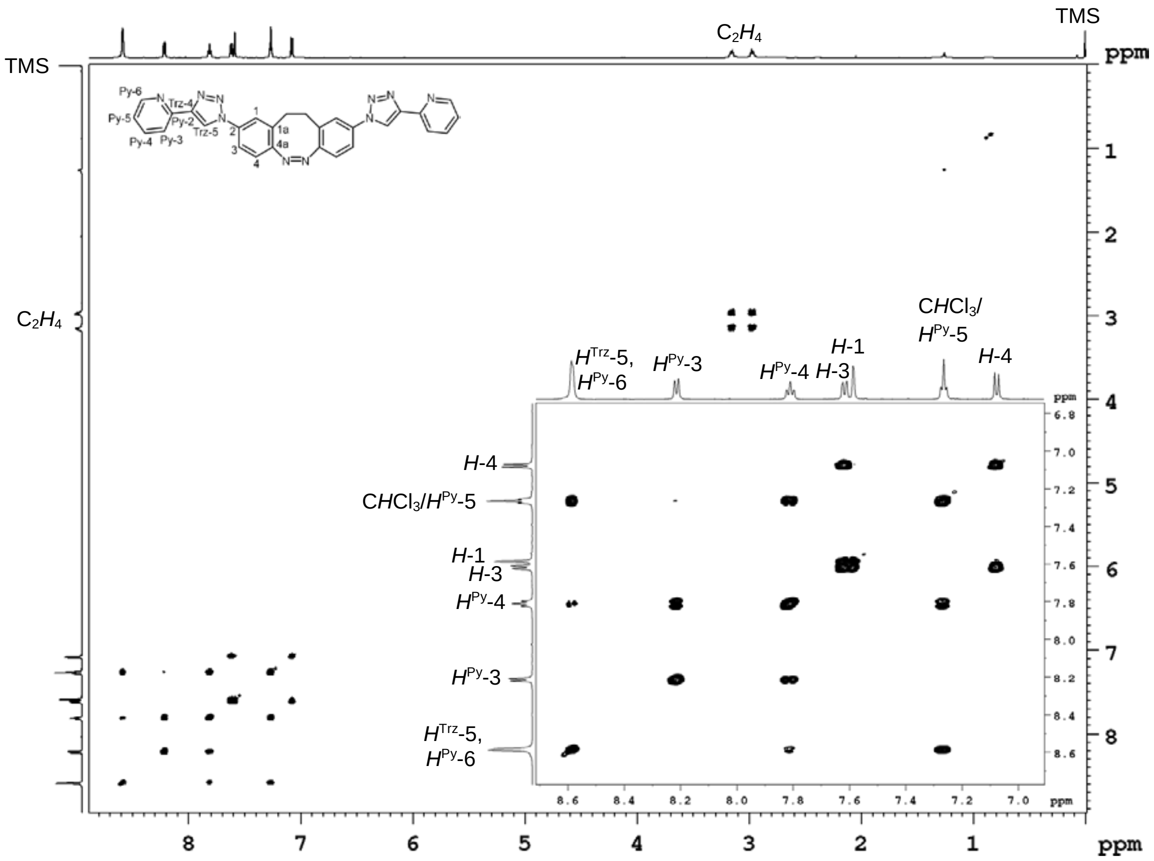

Figure S20: ^{1}H-^{1}H COSY NMR spectrum (600 MHz, chloroform-d_1, 298 K) of ligand **1**.

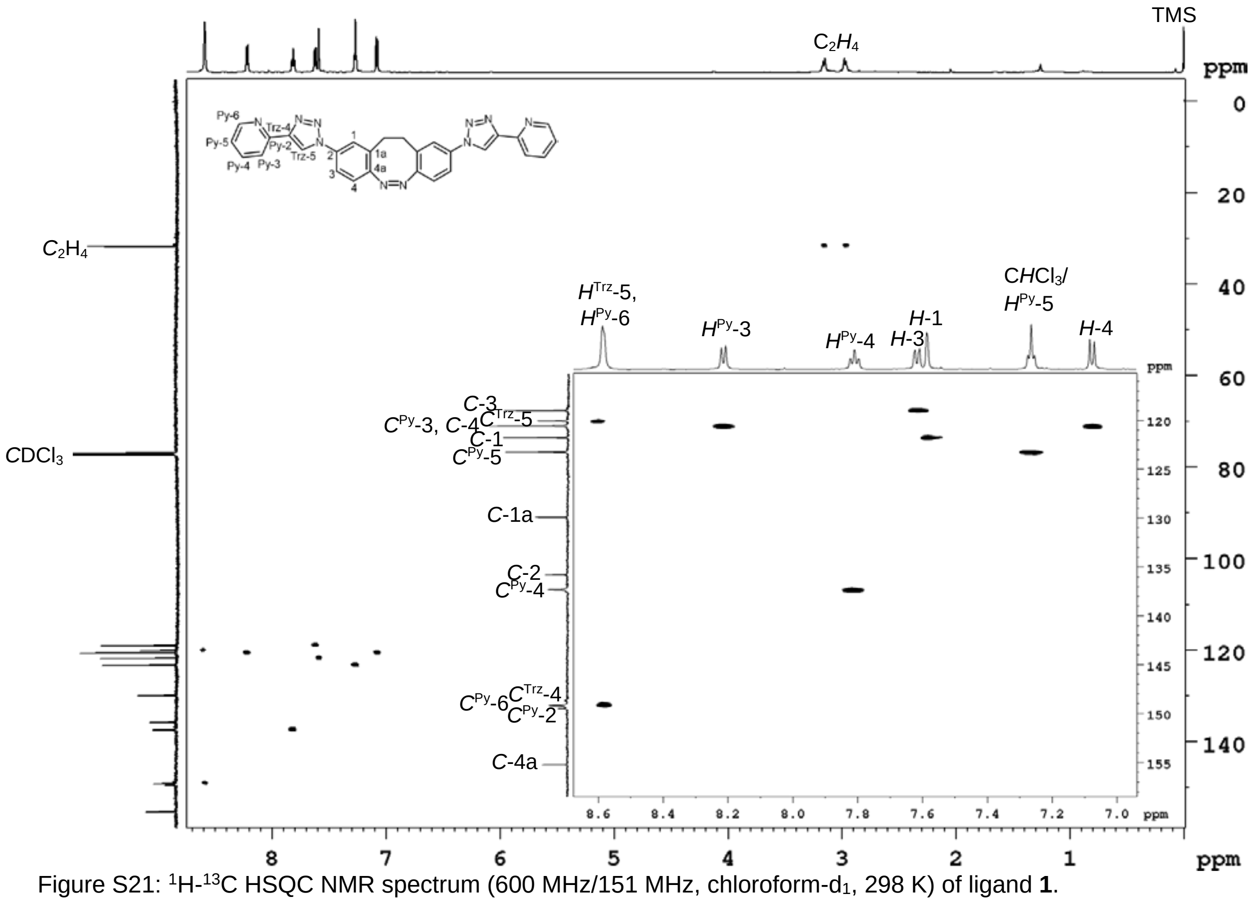

Figure S21: ^{1}H-^{13}C HSQC NMR spectrum (600 MHz/151 MHz, chloroform-d_1, 298 K) of ligand **1**.

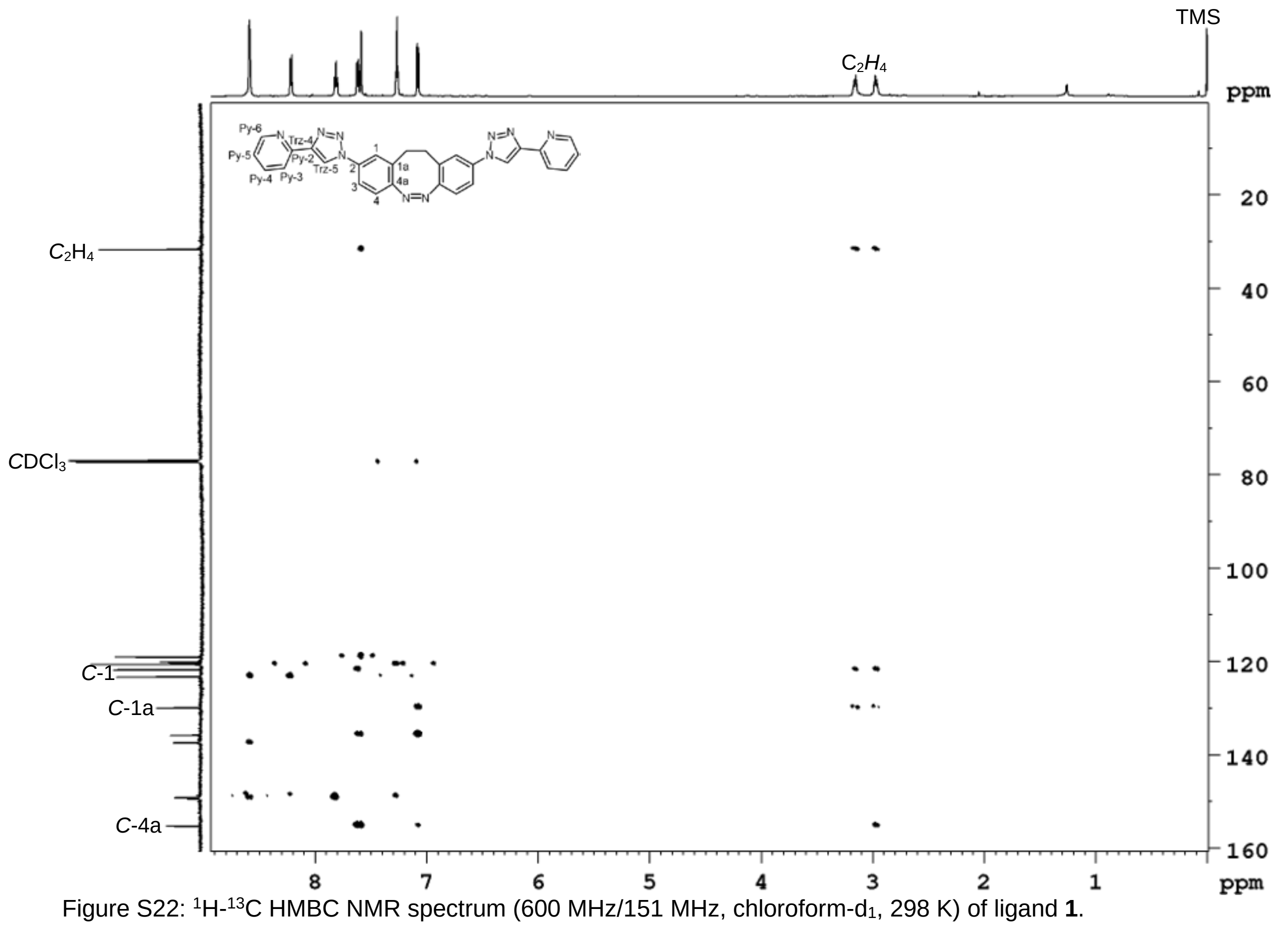

Figure S22: ^{1}H-^{13}C HMBC NMR spectrum (600 MHz/151 MHz, chloroform-d_1, 298 K) of ligand **1**.

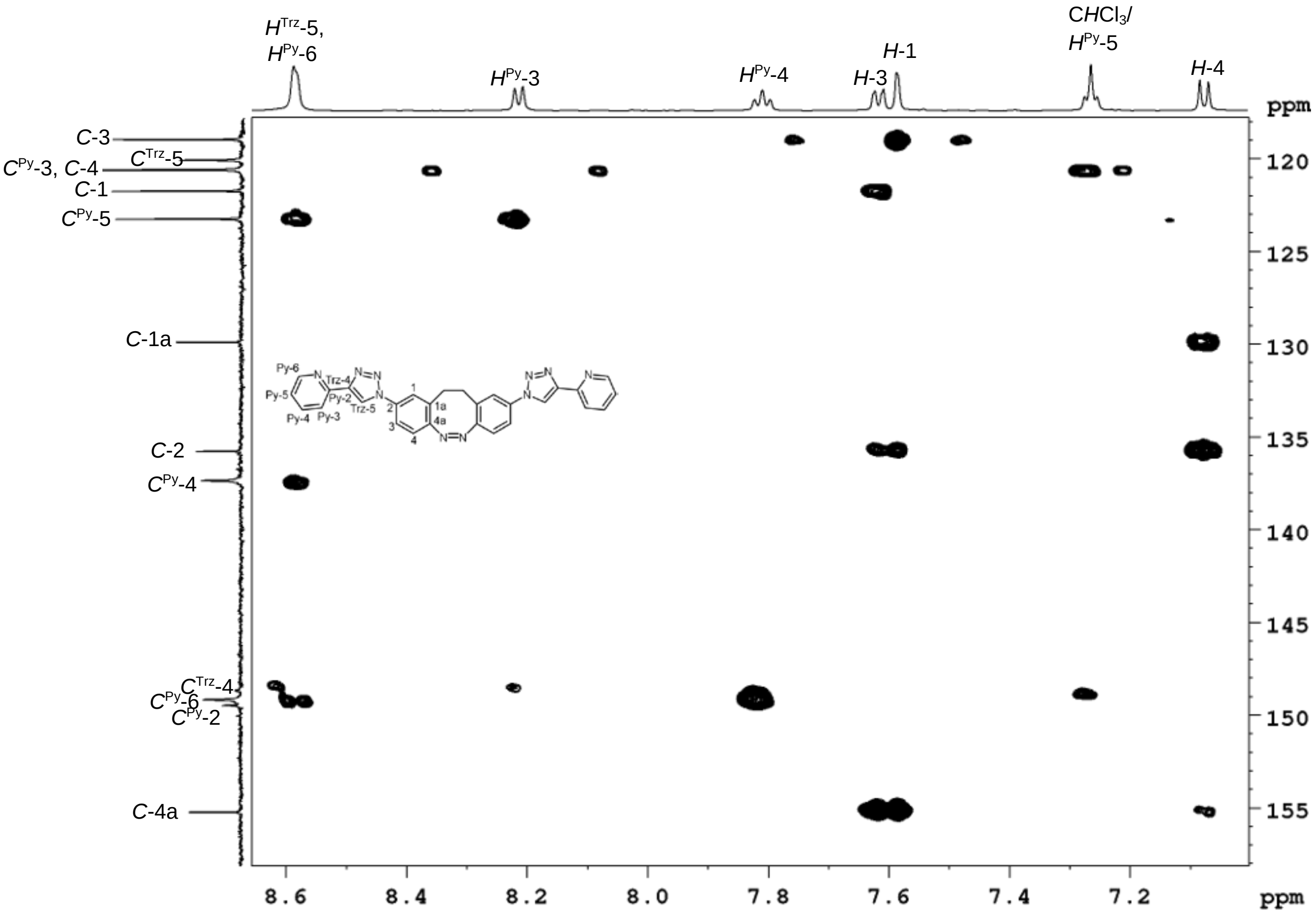

Figure S22.1: Zoomed region of the ^{1}H-^{13}C HMBC NMR spectrum (600 MHz/151 MHz, chloroform-d$_1$, 298 K) of ligand **1**.

3. Self-Assemblies

3.1 Co_2(1-*Z*)$_3$

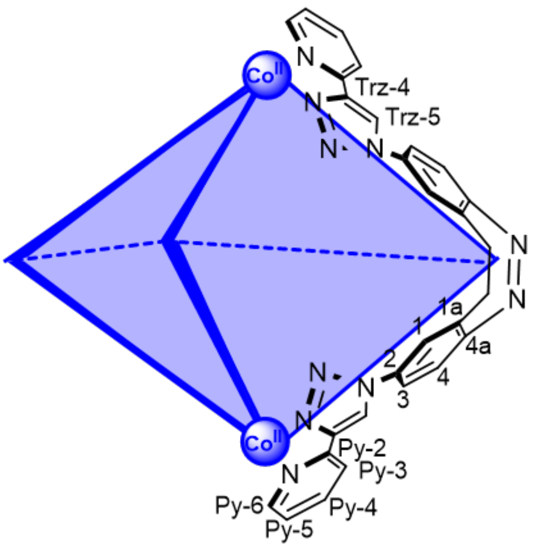

2,9-Bis(4-pyridin-2-yl)-1*H*-1,2,3-triazol-1-yl)-11,12-dihydrodibenzo[*c,g*][1,2]diazocine (**1**, 9.98 mg, 20.0 µmol) and cobalt(II) tetrafluoroborate hexahydrate (4.58 mg, 13.4 µmol) were dissolved in acetonitrile (3 mL). The solution was gently shaken for 2 min, added to diethyl ether (10 mL) and centrifuged (10 min, 3000 rpm) followed by removal of the solvent, addition of diethyl ether (10 mL) and centrifuged (10 min, 3000 rpm). The resulting solid was dried for 3 h under reduced pressure and exclusion of light to obtain a yellow solid (Co_2(**1**-*Z*)$_3$, 13 mg).

^{1}H NMR (600 MHz, acetonitrile-d$_3$, 298 K): δ = 79.9 (s_{br}, 2H, H^{Py}-6), 66.3 (s, 2H, H^{Py}-3), 47.4 (s, 2H, H^{Py}-5), 41.6 (s, 2H, H^{Trz}-5), 14.9 (s, 2H, H^{Py}-4), 12.3 (s, 2H, *H*-3), 9.1 (s, 2H, *H*-4), 0.1 (s, 2H, $C_2H_4^{ax./eq.}$), -2.2 (s, 2H, *H*-1), -4.3 (s, 2H, $C_2H_4^{ax./eq.}$) ppm.

^{13}C NMR (125 MHz, acetonitrile-d$_3$, 298 K): δ = 644.3 (s), 605.8 (d, $^1J_{CH}$ = 181 Hz, C^{Py}-5), 513.7 (d, $^1J_{CH}$ = 158 Hz, C^{Py}-3), 255.9 (s), 221.2 (d, $^1J_{CH}$ = 191 Hz, C^{Trz}-5), 180.0 (d, $^1J_{CH}$ = 167 Hz, *C*-3), 154.8 (s), 145.7 (d, $^1J_{CH}$ = 157 Hz, *C*-4), 136.8 (d, $^1J_{CH}$ = 161 Hz, C^{Py}-4), 125.7 (s), 108.2 (d, $^1J_{CH}$ = 157 Hz, *C*-1), 90.0 (unresolved d), 26.0 (t, $^1J_{CH}$ = 126 Hz, $C_2H_4^{ax./eq.}$), -4.8 (s), -181.9 (s) ppm.

MS (ESI): *m/z* = 1867.4415 [Co_2(**1**-*Z*)$_3$+3BF_4]$^+$, 890.2171 [Co_2(**1**-*Z*)$_3$+2BF_4]$^{2+}$, 564.4772 [Co_2(**1**-*Z*)$_3$+BF_4]$^{3+}$, 525.6538 [Co_1(**1**-*Z*)$_2$]$^{2+}$, 401.8572 [Co_2(**1**-*Z*)$_3$]$^{4+}$.

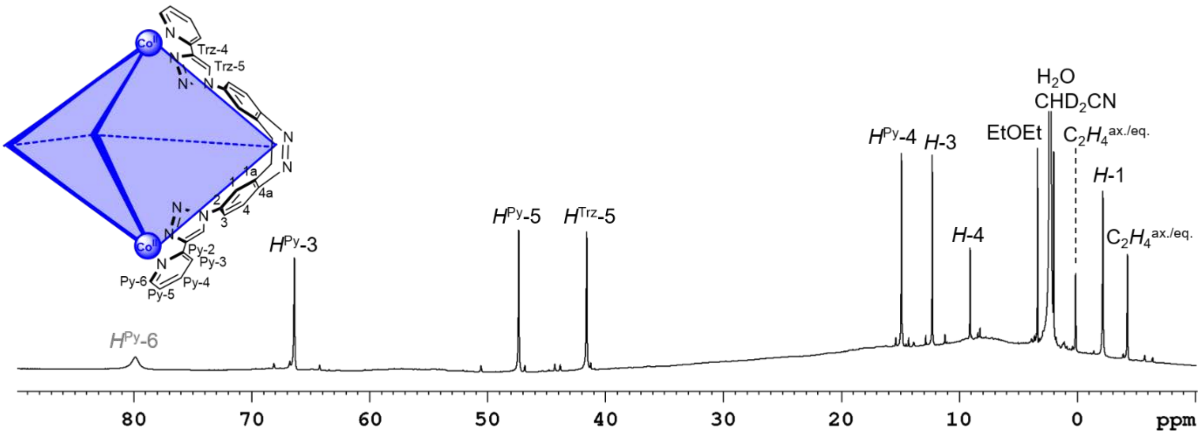

Figure S23: ^{1}H NMR spectrum (600 MHz, acetonitrile-d3, 298 K) of Co_2(**1**-*Z*)$_3$.

The ^{13}C spectra (Figures S24-S25) were assigned based on the HMQC spectra (Figures S27-S28). The tertiary carbon C^{Py}-6 could not be assigned unambiguously due to the absence of a cross-peak in the HMQC, most likely due to the broad linewidth (> 70 Hz) in the ^{1}H NMR spectrum.[1] The assignment of this carbon and the quaternary carbon signals (singlets) were tentatively assigned in grey based on related complexes.[4]

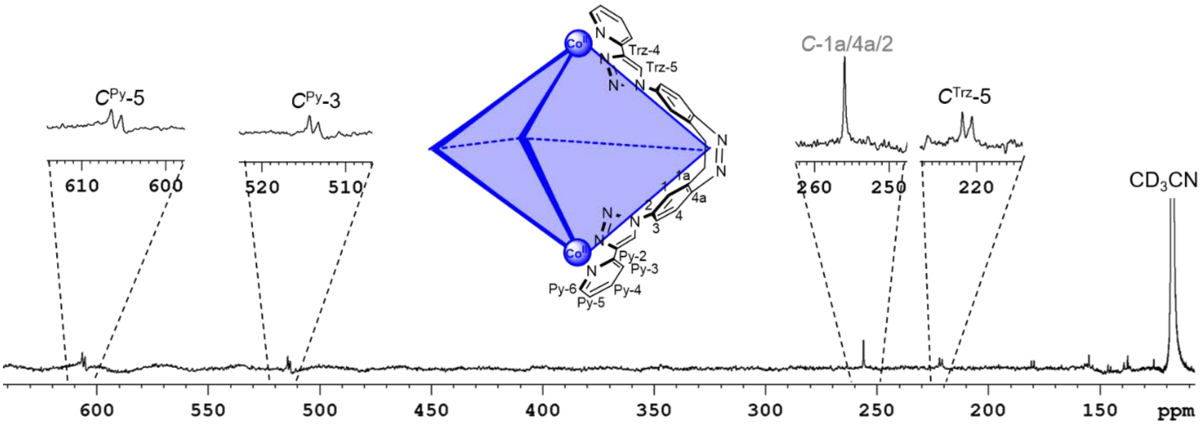

Figure S24: ^{13}C NMR spectrum (125 MHz, acetonitrile-d3, 298 K) of Co_2(**1**-*Z*)$_3$.

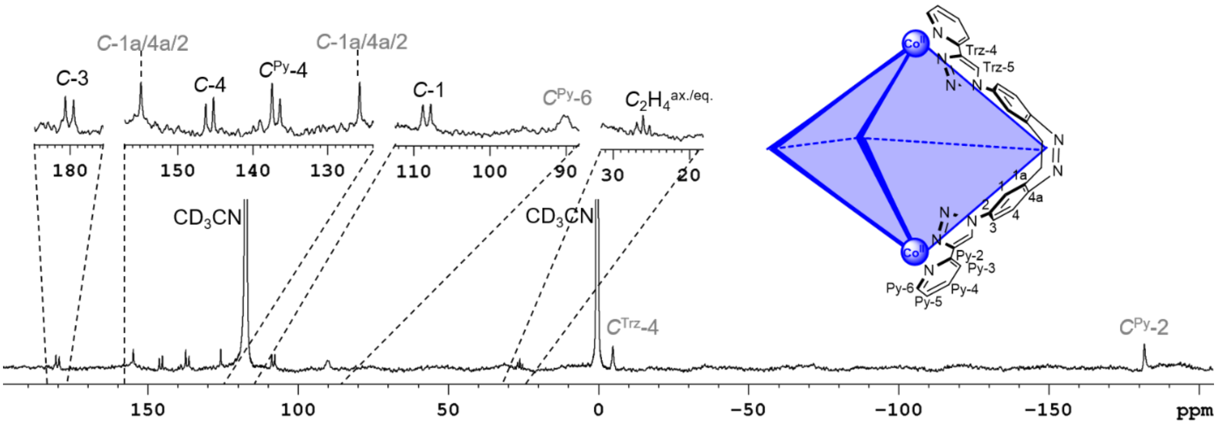

Figure S25: ^{13}C NMR spectrum (125 MHz, acetonitrile-d3, 298 K) of Co_2(**1**-*Z*)$_3$.

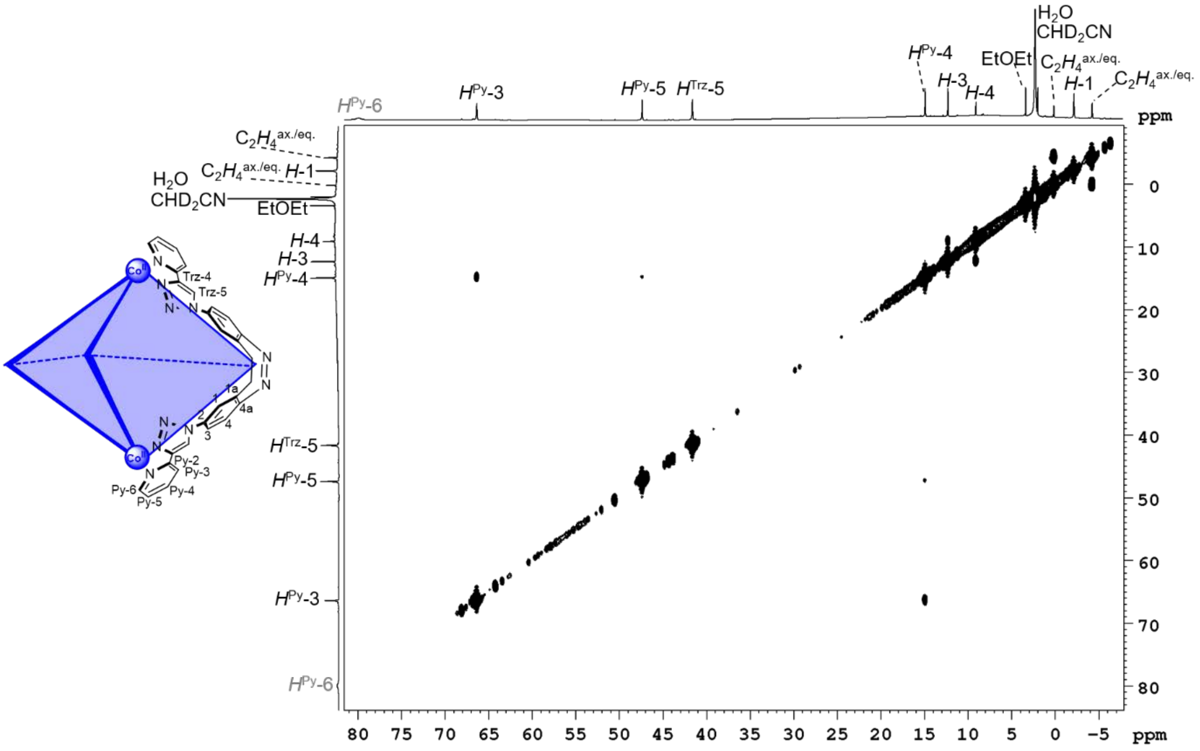

Figure S26: ^{1}H-^{1}H COSY spectrum (600 MHz, acetonitrile-d$_3$, 298 K) of Co_2(**1**-*Z*)$_3$.

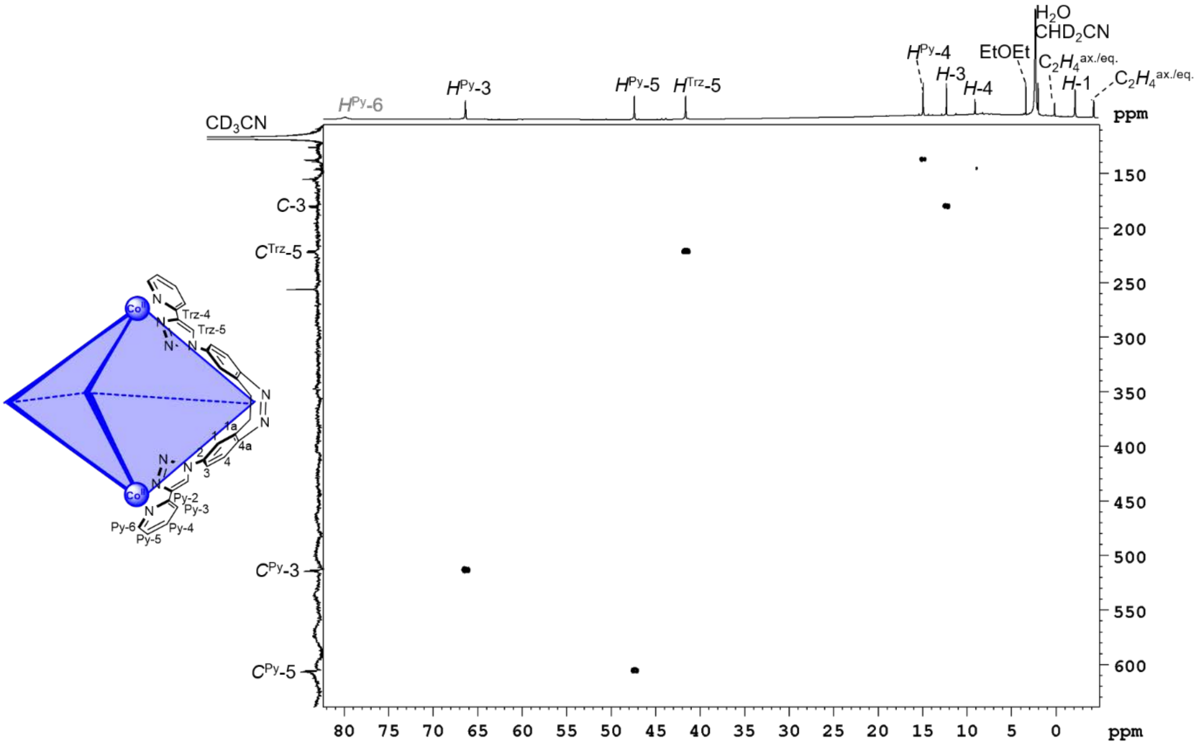

Figure S27: HMQC spectrum (600 MHz, acetonitrile-d$_3$, 298 K) of Co_2(**1**-*Z*)$_3$.

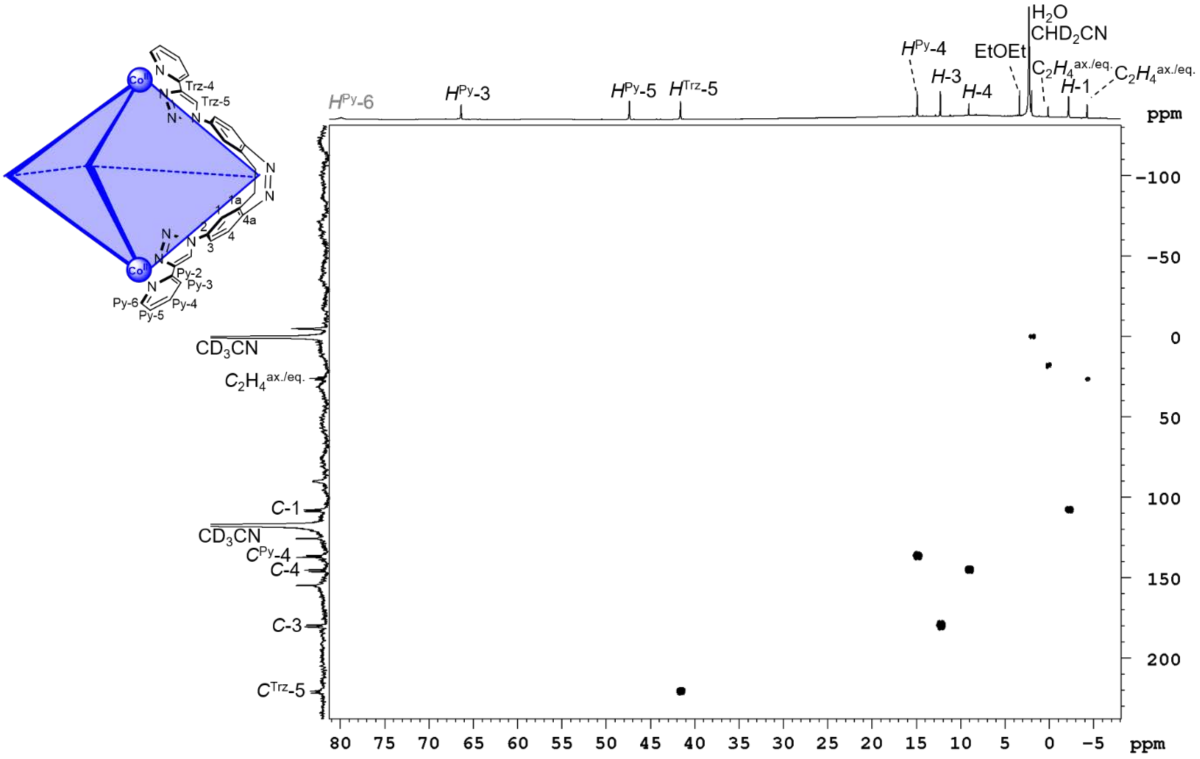

Figure S28: HMQC spectrum (600 MHz, acetonitrile-d3, 298 K) of Co_2(**1**-*Z*)$_3$.

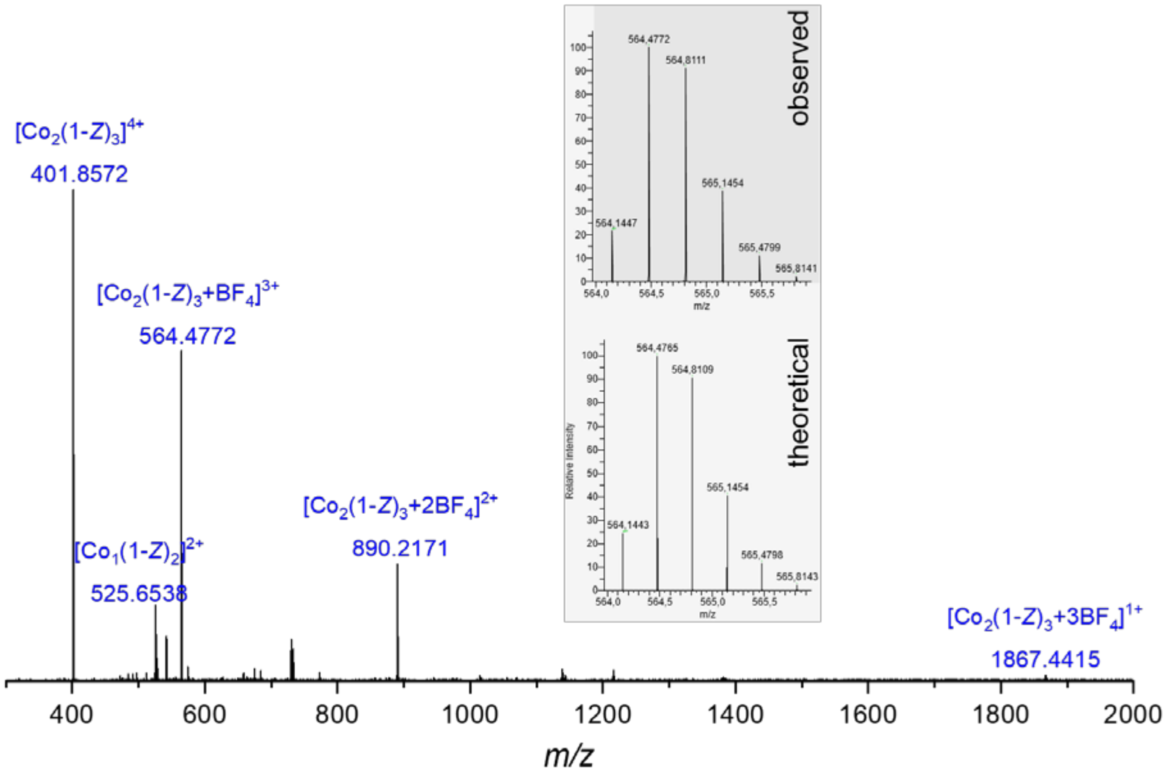

Figure S29: HR ESI mass spectrum of Co_2(**1**-*Z*)$_3$ in the insets the observed and theoretical isotope patterns.

Heating an NMR sample of isolated Co_2(**1**-*Z*)$_3$ for 20 h at 50 °C showed no change in signals in the ^{1}H NMR spectrum as shown in Figure S30.

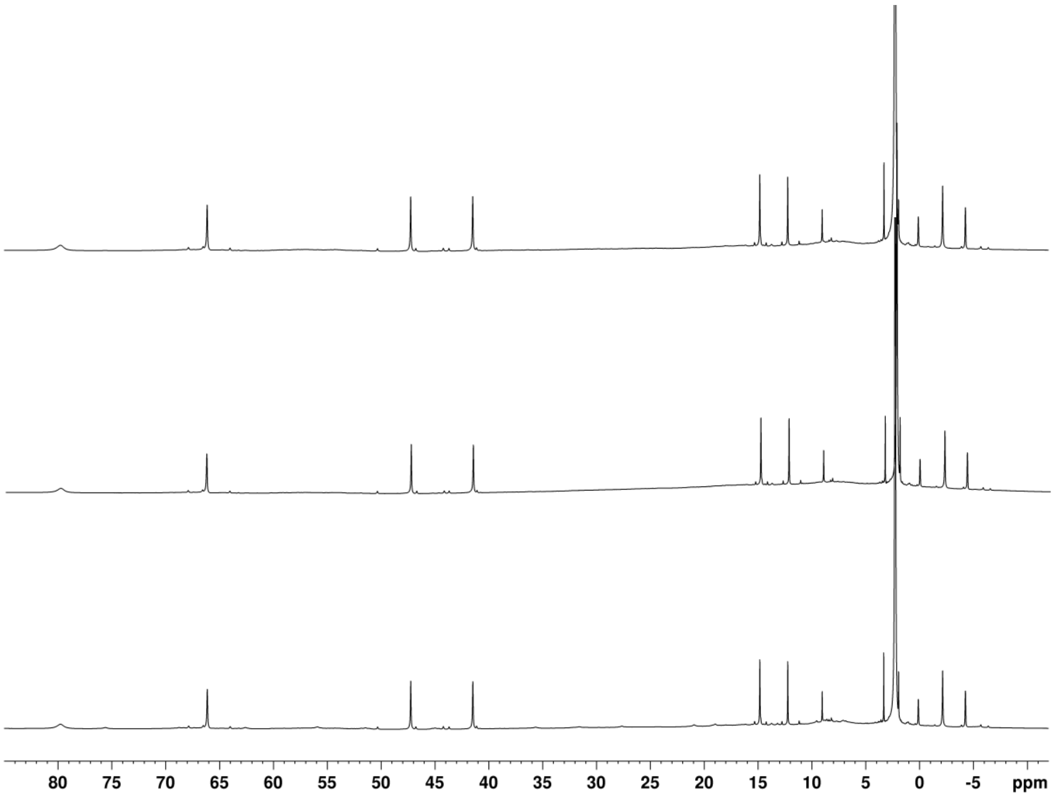

Figure S30: ^{1}H NMR spectrum of Co_2(**1**-*Z*)$_3$ (600 MHz, acetonitrile-d_3, 298 K) after preparation as described above (bottom), after standing at room temperature for 6 months and after (top) heating for 20 h at 50 °C.

3.2 Mixture of Ill-Defined Species with 1-*E* and $Co(BF_4)_2$

Co_2(**1**-*Z*)$_3$ (prepared as described in Section 3.1, 6.51 mg, 3.00 µmol) was dissolved in 0.5 mL of acetonitrile-d_3 and the sample was irradiated with 385 nm for 2 min in order to obtain the mixture of ill-defined species formed with ligand **1**-*E* and $Co(BF_4)_2$ (Figure S31).

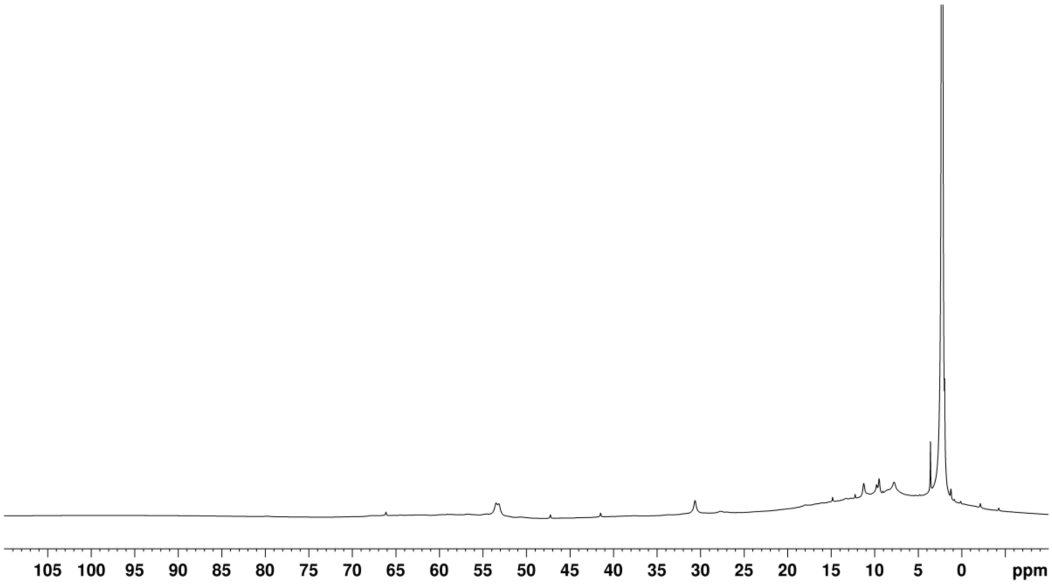

Figure S31: ^{1}H NMR spectrum (600 MHz, acetonitrile-d_3, 298 K) of the mixture of ill-defined species containing ligand **1**-*E* and $Co(BF_2)_4$.

3.3 Co_2(2-*E*)$_3$

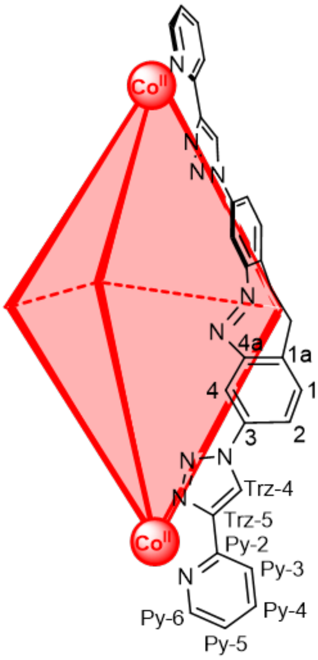

3,8-Bis(4-pyridin-2-yl)-1*H*-1,2,3-triazol-1-yl)-11,12-dihydrodibenzo[*c,g*][1,2]diazocine (**2,** 9.98 mg, 20.0 µmol) and cobalt(II) tetrafluoroborate hexahydrate (4.58 mg, 13.4 µmol) were dissolved in acetonitrile (3 mL). The solution was irradiated with 385 nm for 40 minutes while air-cooling and stirring. The bright red solution was added to diethyl ether (10 mL) and centrifuged (10 min, 3000 rpm) followed by removal of the solvent, addition of diethyl ether

(10 mL) and centrifuged (10 min, 3000 rpm). The resulting solid was dried for 45 minutes *in vacuo* and exclusion of light to obtain a red solid (Co_2(**2**-*E*)$_3$, 12 mg).

^{1}H NMR (600 MHz, acetonitrile-d$_3$, 298 K): δ = 98.5 (s$_{br}$, 2H, H^{Py}-6), 61.8 (s, 2H, H^{Py}-3), 49.5 (s, 2H, H^{Py}-5), 37.7 (s, 2H, H^{Trz}-5), 13.8 (s, 2H, H^{Py}-4), 11.9 (s, 2H, *H*-2), 9.1 (s, 2H, *H*-1), 2.1 (s, 2H, $C_2H_4^{ax./eq.}$), 1.5 (s, 2H, $C_2H_4^{ax./eq.}$), -4.7 (s, 2H, *H*-4) ppm.

^{13}C NMR (125 MHz, acetonitrile-d$_3$, 298 K): δ = 597.8 (d, $^1J_{CH}$ = 163 Hz, C^{Py}-5), 510.6 (d, $^1J_{CH}$ = 161 Hz, C^{Py}-3), 257.0 (s), 228.8 (d, $^1J_{CH}$ = 173 Hz, C^{Trz}-5), 172.9 (d, $^1J_{CH}$ = 173 Hz, *C*-2), 153.0 (s), 151.8 (d, $^1J_{CH}$ = 156 Hz, *C*-1), 137.5 (d, $^1J_{CH}$ = 179 Hz, C^{Py}-4), 135.2 (s), 98.4 (unresolved d), 97.4 (d, $^1J_{CH}$ = 166 Hz, *C*-4), 29.6 (t, $^1J_{CH}$ = 119 Hz, $C_2H_4^{ax./eq.}$), -19.6 (s), -187.5 (s) ppm.

MS (ESI, acetonitrile): *m/z* = 564.4756 [Co_2(**2**-*E*)$_3$+BF_4]$^{3+}$, 525.6535 [Co_1(**2**-*E*)$_2$]$^{2+}$, 401.8570 [Co_2(**2**-*E*)$_3$]$^{4+}$.

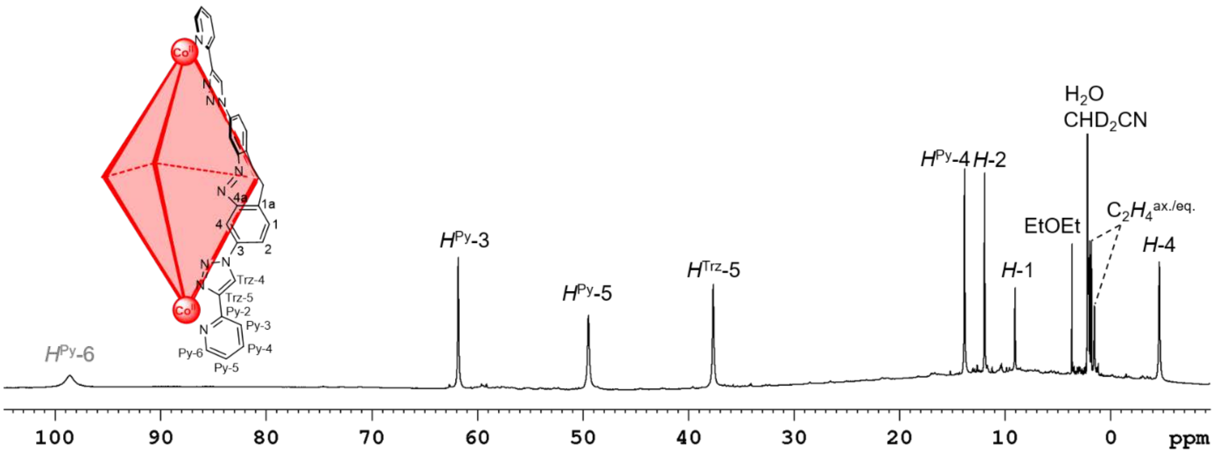

Figure S32: ^{1}H NMR spectrum (600 MHz, acetonitrile-d$_3$, 298 K) of Co_2(**2**-*E*)$_3$.

The ^{13}C spectra (Figures S33-S34) were assigned based on the HMQC spectra (Figures S36-S37). The tertiary carbons C^{Py}-5 and C^{Py}-6 could not be assigned unambiguously due to the absence of a cross-peak in the HMQC, most likely due to the broad linewidth (> 70 Hz) in the ^{1}H NMR spectrum.[1] The assignment of these carbons and the quaternary carbon signals (singlets) were tentatively assigned in grey based on related complexes.[4]

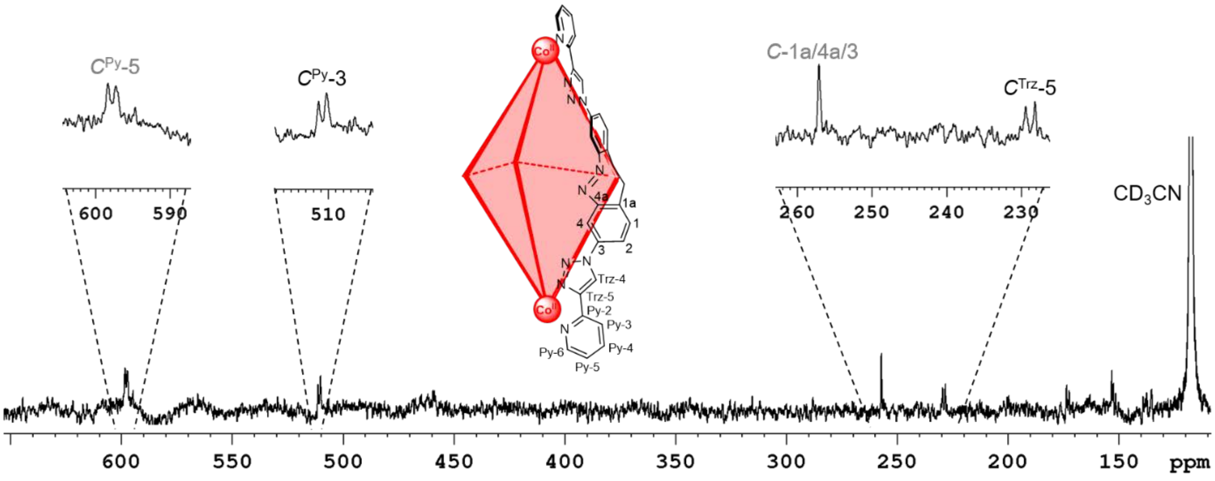

Figure S33: ^{13}C NMR spectrum (125 MHz, acetonitrile-d$_3$, 298 K) of Co_2(**2**-*E*)$_3$.

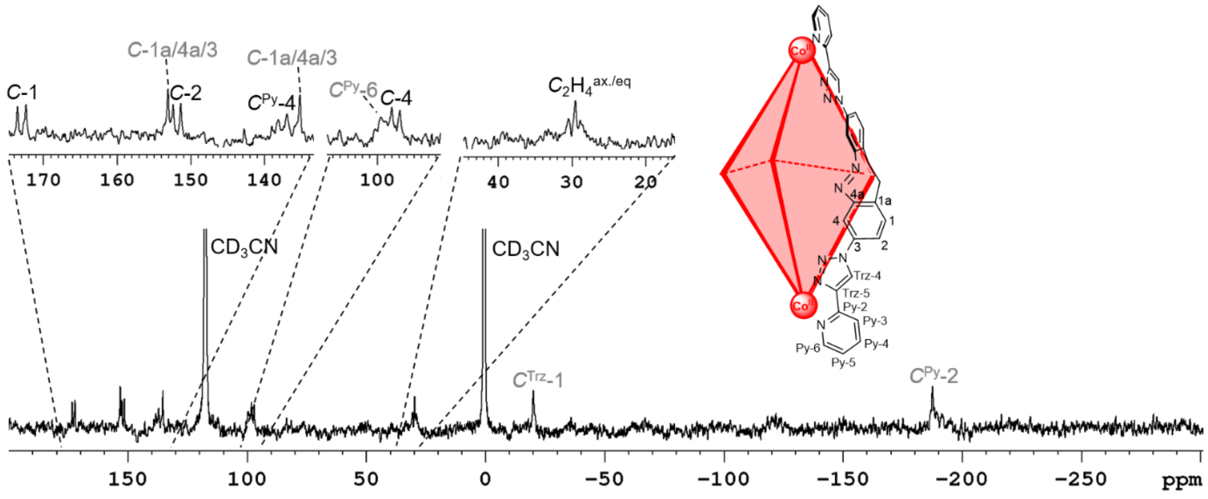

Figure S34: ^{13}C NMR spectrum (125 MHz, acetonitrile-d3, 298 K) of Co2(**2**-*E*)3.

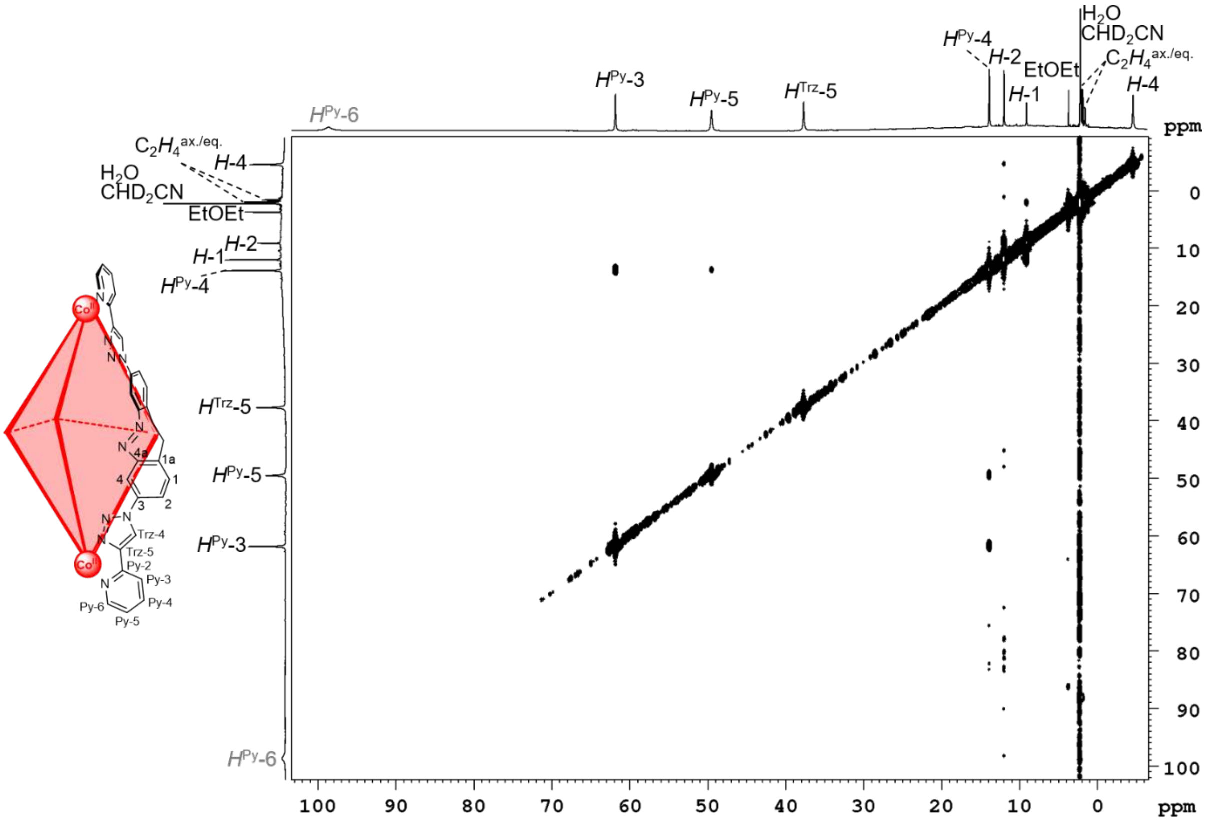

Figure S35: ^{1}H-^{1}H COSY spectrum (600 MHz, acetonitrile-d3, 298 K) of Co2(**2**-*E*)3.

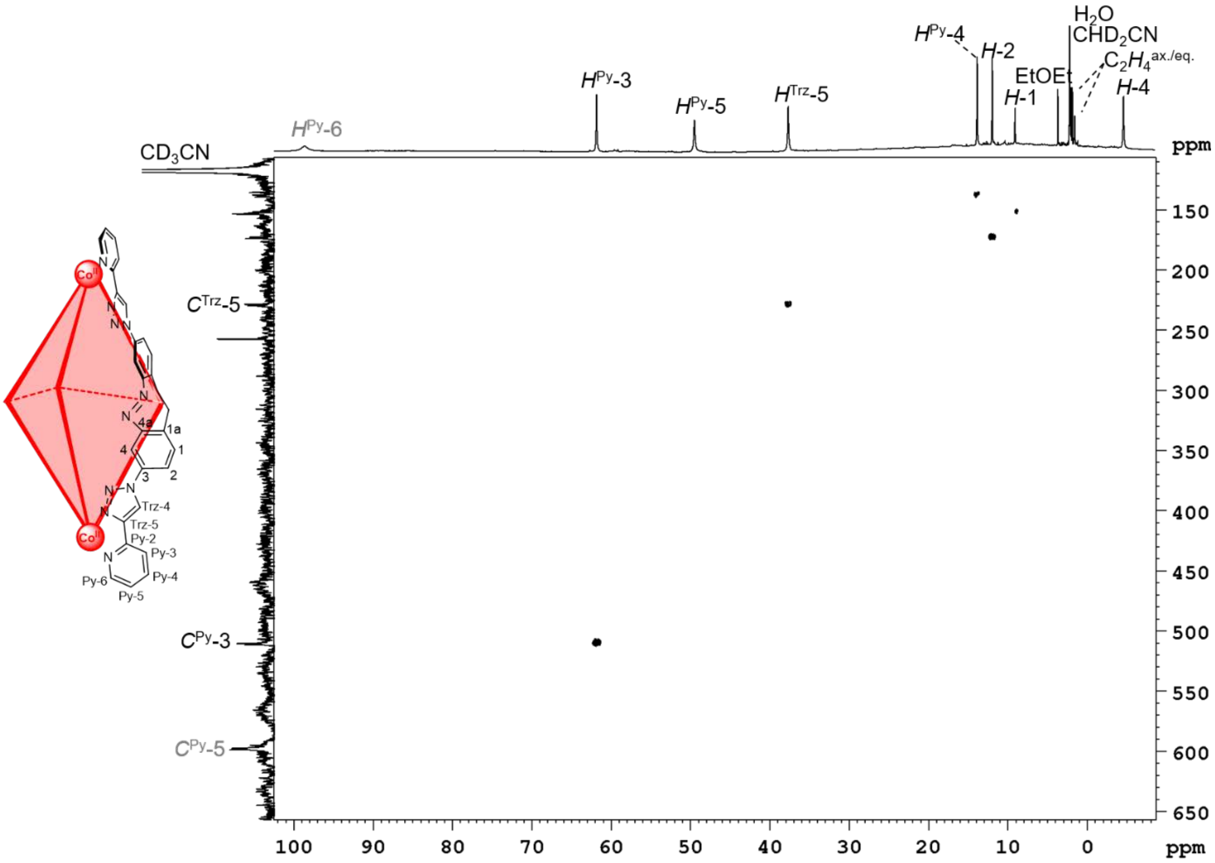

Figure S36: HMQC spectrum (600 MHz, acetonitrile-d$_3$, 298 K) of Co$_2$(**2**-*E*)$_3$.

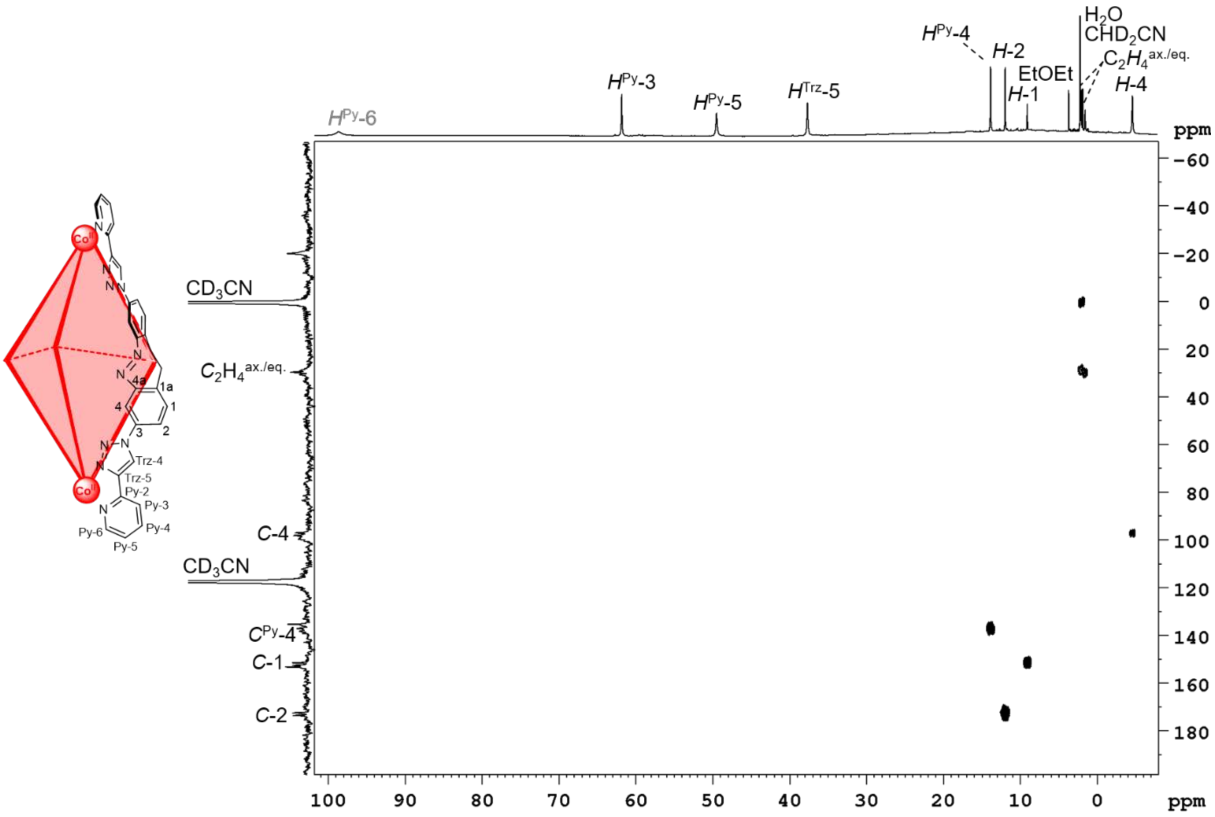

Figure S37: HMQC spectrum (600 MHz, acetonitrile-d$_3$, 298 K) of Co$_2$(**2**-*E*)$_3$.

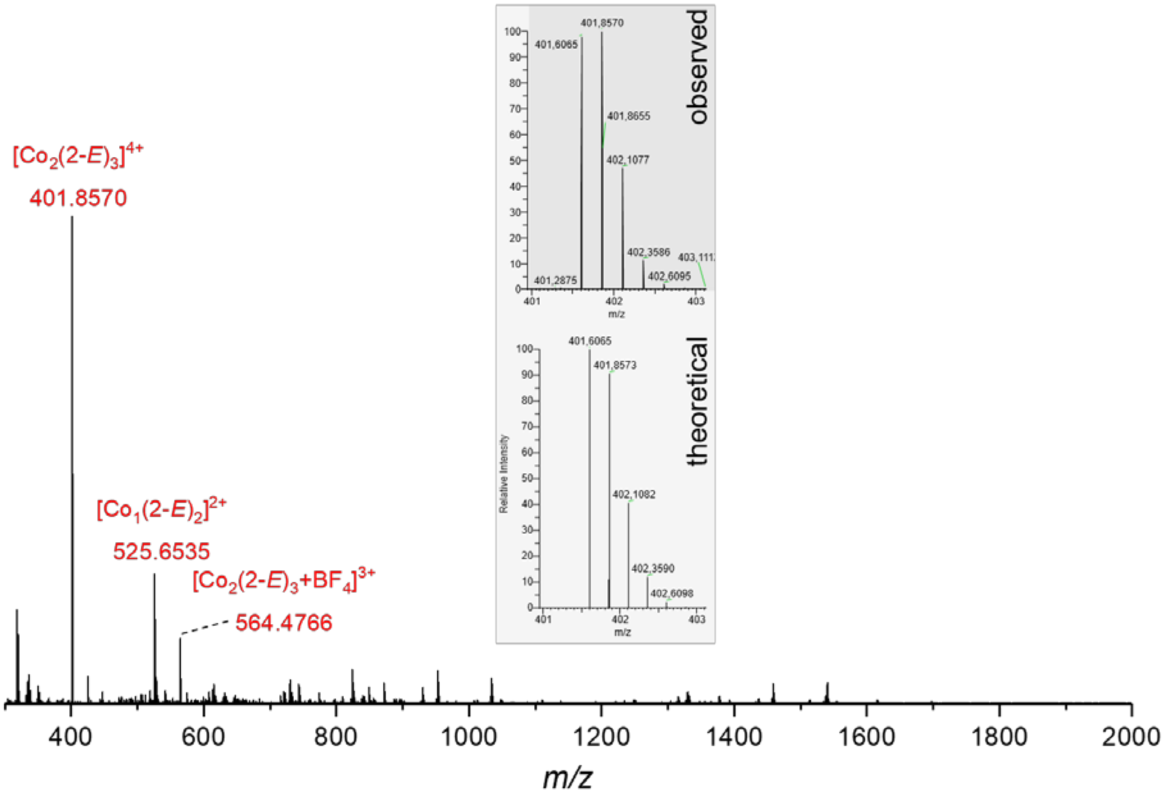

Figure S38: HR-ESI mass spectrum of Co_2(**2**-*E*)$_3$ in the insets the observed and theoretical isotope patterns.

3.4 Mixture of Ill-Defined Species with 2-*Z* and $Co(BF_4)_2$

Co_2(**2**-*E*)$_3$ was prepared as described in Section 3.3. An NMR sample with a concentration of 6 mM in acetonitrile-d_3 was then irradiated with 520 nm for 2 min in order to obtain the mixture of ill-defined species formed with ligand **2**-*Z* and $Co(BF_4)_2$ (Figure S39).

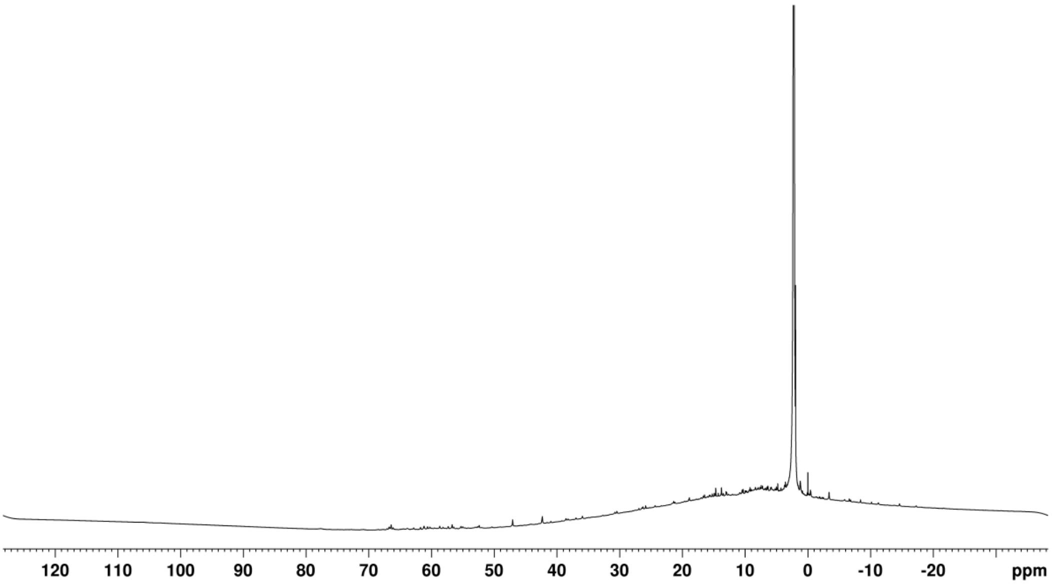

Figure S39: ^{1}H NMR spectrum (600 MHz, acetonitrile-d_3, 298 K) of ligand **2**-*Z* and $Co(BF_4)_2$.

Heating of the NMR sample for 20 h at 50 °C showed no change in signals in the ^{1}H NMR spectrum as shown in Figure S40.

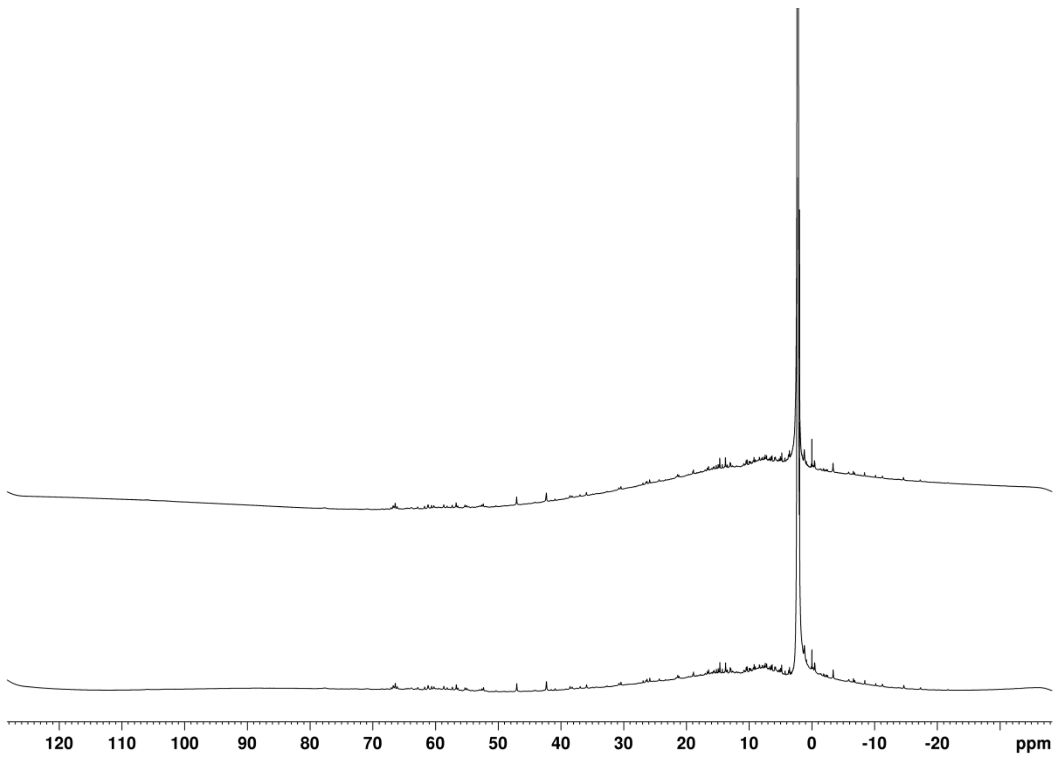

Figure S40: ^{1}H NMR spectrum (600 MHz, acetonitrile-d$_3$, 298 K) of the mixture of ill-defined species formed with ligand **2**-*Z* and $Co(BF_4)_2$ before (bottom) and after (top) heating for 20 h at 50 °C.

4. Photochemical characterization of the ligands

The photoswitching properties of the two ligands **1** and **2** in acetonitrile were investigated via NMR spectroscopy and UV/vis spectroscopy. Both ligands were converted from their thermodynamically stable *Z*-configuration into the metastable *E*-form by excitation of the corresponding n-π* bands (λ_{max}: **1**: 402 nm, **2**: 399 nm). An irradiation wavelength of 385 nm was chosen to minimise back-isomerisation from simultaneous excitation of the n-π* transition (491 nm for ligand **1** and 485 nm for ligand **2**).

The photostationary state (PSS) for *Z* to *E* photoisomerization was determined via ^{1}H NMR measurements through integration of the Trz-5 protons for the *Z*- and *E*-configurations following irradiation with 385 nm for 2 minutes at 298 K. The PSS was found to be 76% for ligand **1** (0.5 mM solution, Figures S41-S42) and 72% for ligand **2** (0.25 mM solution, Figures S45-S46). These lower conversion rates compared to the parent diazocine (92%)[5] were attributed to band overlap due to slightly red-shifted π-π* absorption bands.

The half-life ($t_{1/2}$) was determined from UV/vis relaxation measurements following irradiation with 385 nm for 2 minutes and UV/vis spectra were measured every 10 min. $\ln(A/A_0)$ at 491 nm (ligand **1**) or 485 nm (ligand **2**) was plotted as a function of time and by linear fitting of the following equation (where m is the slope), the thermal half-life was determined to be 2.0 hours for ligand **1** (Figures S43-S44) and 5.5 hours for ligand **2** (Figures S47-S48).

$$t = \frac{ln2}{-m}$$

For the photochemical experiments, the following amounts of the respective ligand were dissolved in acetonitrile (deuterated for the NMR experiments). The samples were prepared under exclusion of light.

Table S1: Amounts of ligands to achieve the needed concentration.

Concentration	Ligand (mg)	Acetonitrile (mL)
0.5 mM (UV/vis)	0.744	3.00
0.5 mM (NMR)	0.124	0.50
0.25 mM (UV/vis)	0.372	3.00
0.25 mM (NMR)	0.062	0.50

4.1 Ligand 1

4.1.1 ^{1}H NMR measurements

The PSS (photostationary state) for ligand **1** (0.5 mM in acetonitrile-d_3) was determined to be 76% (Figures S41-S42).

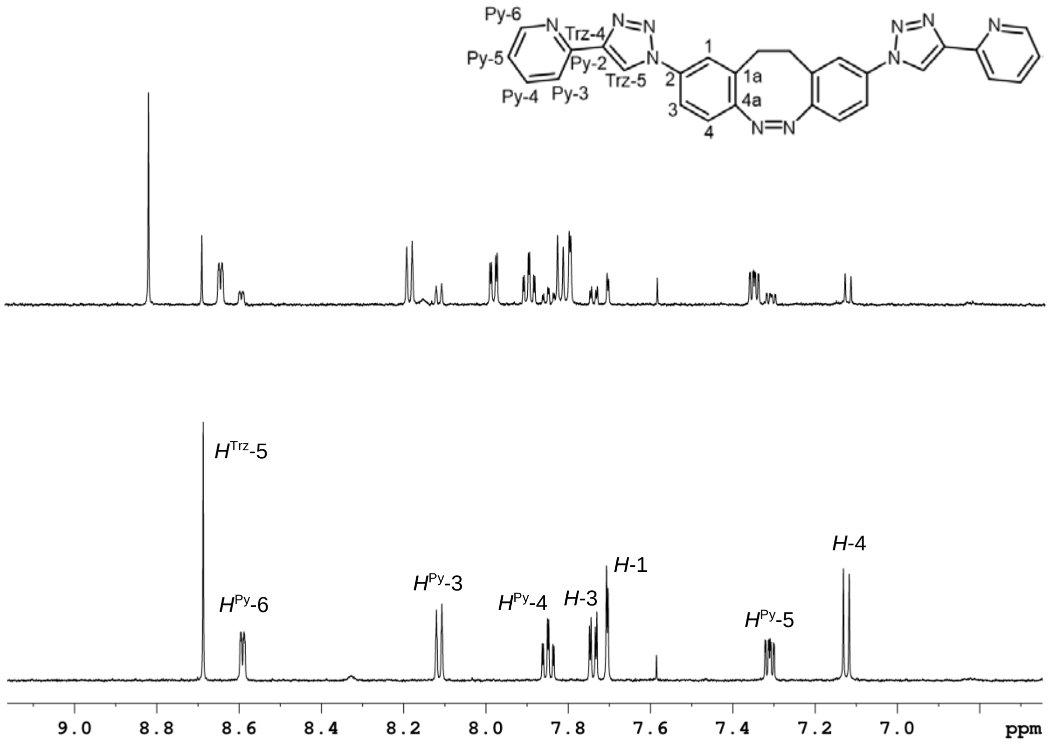

Figure S41: ^{1}H NMR spectrum (600 MHz, acetonitrile-d$_3$, 298 K) of aromatic region of ligand **1** before (bottom) and after irradiation with 385 nm for 2 min (top).

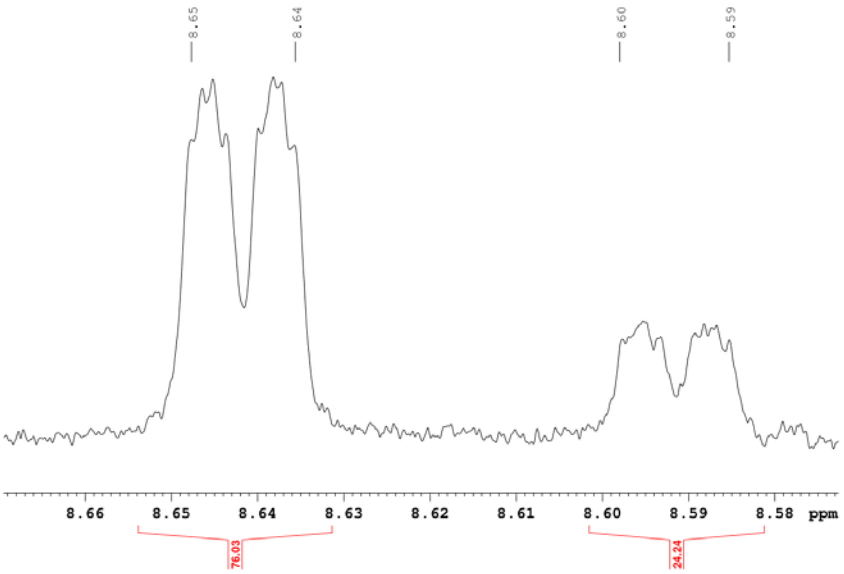

Figure S42: Zoomed region of the ^{1}H NMR spectrum (600 MHz, acetonitrile-d$_3$, 298 K) of ligand **1** after irradiation with 385 nm for 2 min and integration for determination of the PSS.

4.1.2 UV/vis measurements

Following conversion of ligand **1** to its metastable *E*-form by irradiation with 385 nm for 2 min, UV/vis relaxation measurements (0.5 mM, acetonitrile, 298.15 K, $\Delta T = 10$ min) and fitting $\ln(A/A_0)$ at 491 nm as a function of time gave the half-life time ($t_{1/2}$) of compound **1** as 2.0 h (Figures 43-44).

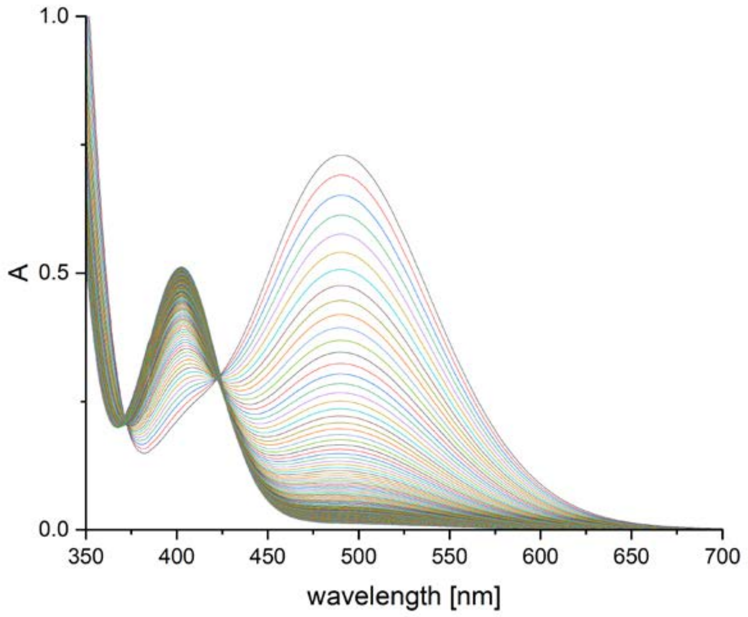

Figure S43: UV/vis relaxation experiment of compound **1** (0.5 mM, acetonitrile, 298.15 K, ΔT=10 min) after irradiation with 385 nm for 2 min and sequential measurement in an interval of 10 minutes.

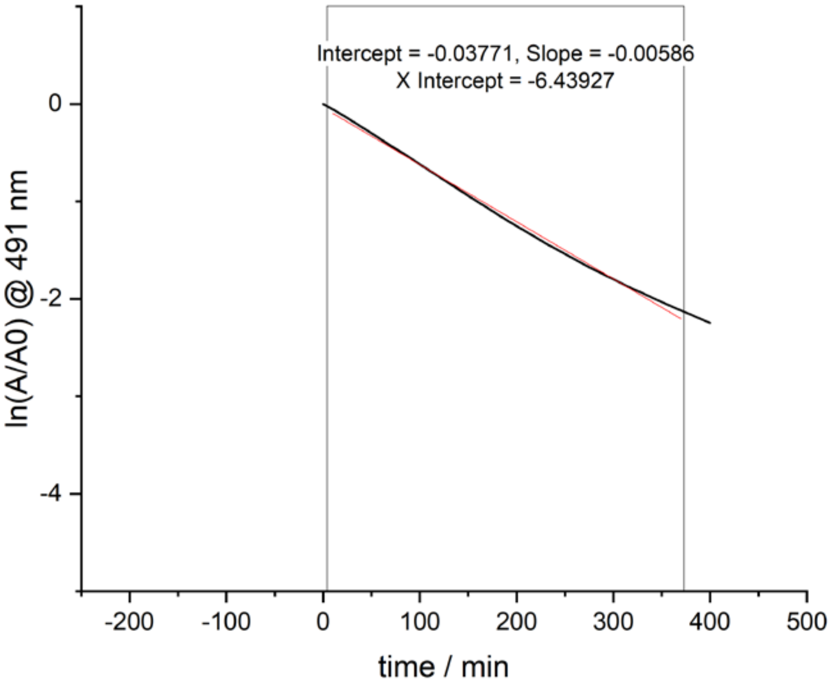

Figure S44: Plot of $\ln(A/A_0)$ measured at 491 nm of compound **1** as a function of time and linear fitting.

4.2 Ligand 2

4.2.1 ^{1}H NMR measurements

The PSS (photostationary state) for ligand **2** (0.25 mM in acetonitrile-d_3) was determined to be 72% (Figures S45-S46).

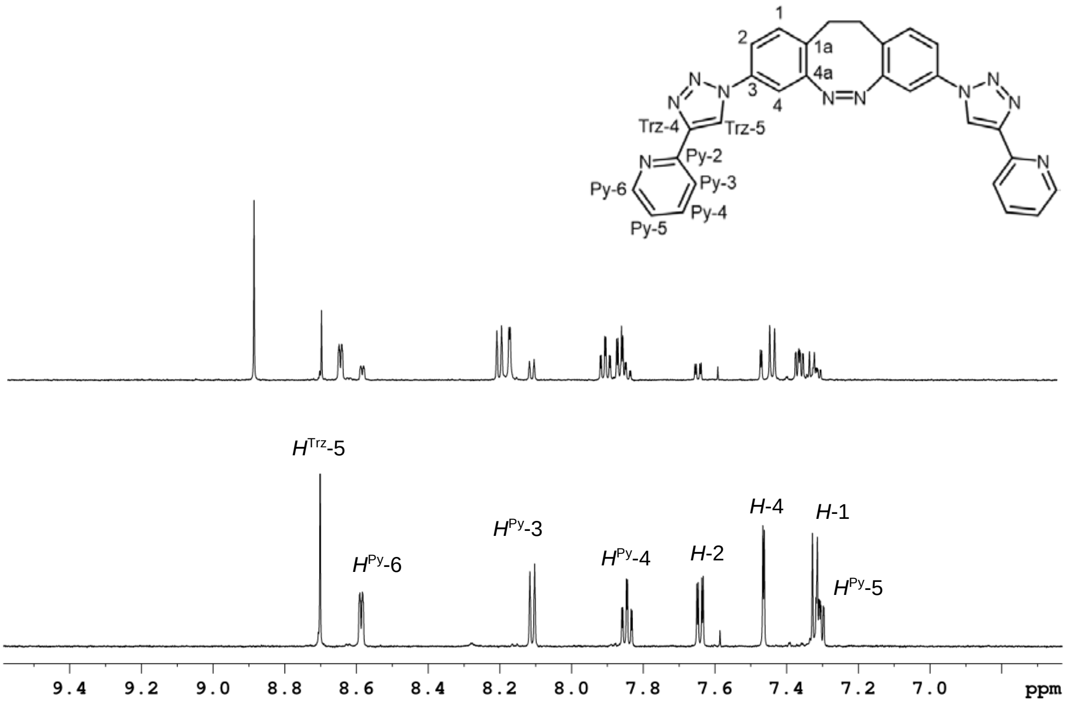

Figure S45: ^{1}H NMR spectrum (600 MHz, acetonitrile-d_3, 298 K) of aromatic region of ligand **2** before (bottom) and after irradiation with 385 nm for 2 min (top).

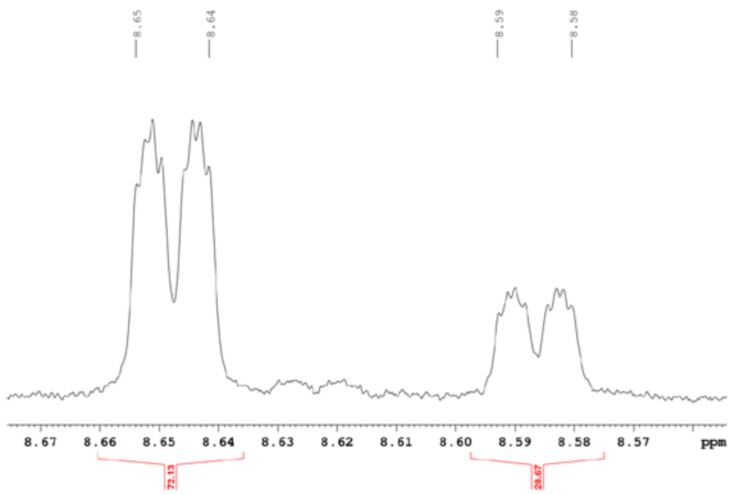

Figure S46: Zoomed region of the ^{1}H NMR spectrum (600 MHz, acetonitrile-d_3, 298 K) of ligand **2** after irradiation with 385 nm for 2 min and integration in order to determine the PSS.

4.2.2 UV/vis measurements

Following conversion of ligand **2** to its metastable *E*-form by irradiation with 385 nm for 2 min, UV/vis relaxation measurements (0.25 mM, acetonitrile, 298.15 K, ΔT = 10 min) and fitting $\ln(A/A_0)$ at 491 nm as a function of time gave the half-life time ($t_{1/2}$) of compound **2** as 5.5 h (Figures 47-48).

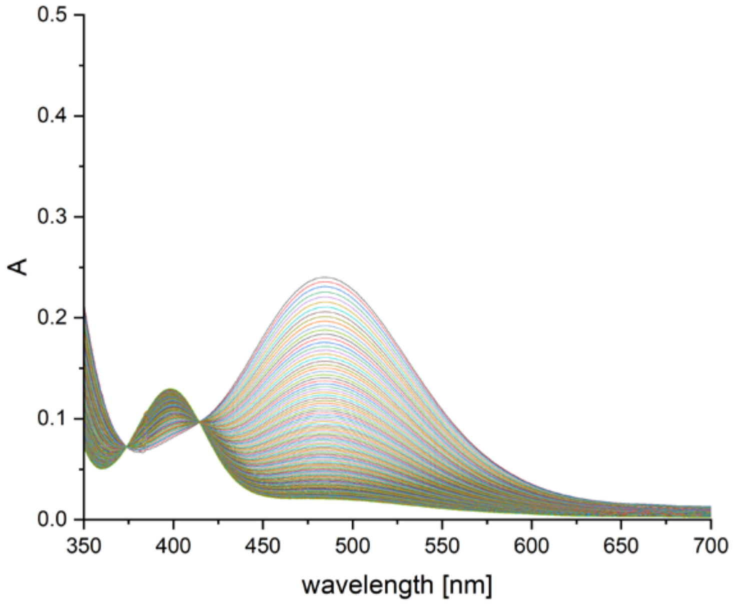

Figure S47: UV/vis relaxation experiment of ligand **2** (0.25 mM, acetonitrile, 298.15 K, ΔT=10 min) after irradiation with 385 nm and sequential measurement in an interval of 10 minutes.

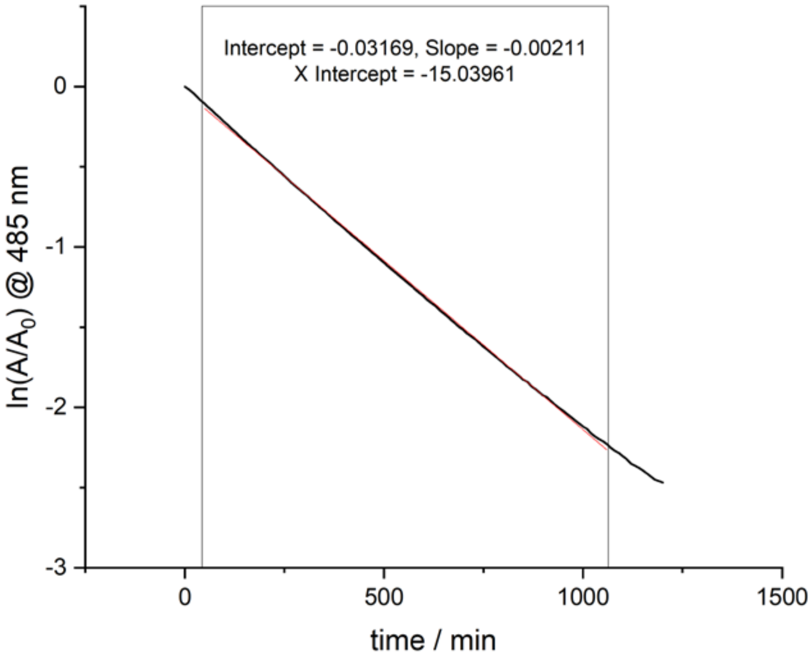

Figure S48: Plot of $\ln(A/A_0)$ measured at 485 nm of ligand **2** as a function of time and linear fitting.

5. Photochemical characterization of the self-assemblies

The photochemical properties of the self-assemblies were investigated via NMR spectroscopy and UV/vis spectroscopy in acetonitrile using similar methods to those in Section 4. Similar absorption maxima were observed for n-π* bands of the ligands in the absence and presence of cobalt(II) ions (Table S2) and therefore, an irradiation wavelength of 385 nm was chosen to minimize back-isomerization from simultaneous excitation of the n-π* transition of the *E*-configuration (491 nm for ligand **1** and 483 nm for ligand **2** in the presence of cobalt(II) ions). It was not possible to quantify the PSS following irradiation due to the paramagnetism of the resulting helicate Co_2(**2**-*E*)$_3$ and the formation of a mixture of ill-defined species with ligand **1**-*Z*. Nevertheless, NMR experiments were carried out to identify the species formed upon photoswitching over multiple cycles and from thermal relaxation (Figures S49-S50 and S55-S56). Furthermore, ^{1}H NMR measurements showed the helicates were stable upon dilution to a concentration of 0.33 mM, the concentration used for UV/vis experiments (Figures S51 and S57).

The half-life ($t_{1/2}$) for both ligands in the presence of Co(II) ions was determined by UV/vis-measurements as 1.6 hours for **1**-*E* (Figures S52-S53) and 9.2 hours for **2**-*E* (Figures S58-S59). The ligands were photostable in the presence of Co(II) ions over multiple cycles using UV/vis spectroscopy (Figures S54 and S60).

Table S3: Photochemical properties of ligands **1** and **2** and their self-assemblies formed with $Co(BF_4)_2$. Note: The PSS for the self-assemblies could not be determined.

Substance	PSS (%)	$t_{1/2}$ (h)	λ_{max} (nm)
Ligand **1**	76	2.0	*Z*: 402, *E*: 491
Ligand **2**	72	5.5	*Z*: 399, *E*: 485
Self-assembly with **1** and $Co(BF_4)_2$	/	9.2	*Z:* 404, *E*: 491
Self-assembly with **2** and $Co(BF_4)_2$	/	1.6	*Z:* 397, *E*: 483

5.1 Self-assemblies with ligand 1

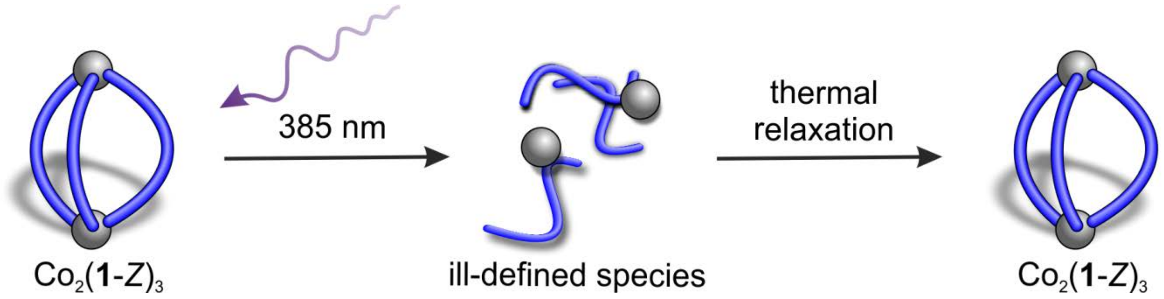

5.1.1 NMR measurements

Reversible photoswitching between Co_2(**1**-*Z*)$_3$ and the mixture of ill-defined species formed from ligand **1**-*E* and Co(II) ions was possible over 20 cycles (Figure S49) and the mixture of

ill-defined species was observed to convert to Co_2(**1**-*Z*)$_3$ following thermal relaxation (Figure S50).

General procedure:

Co_2(**1**-*Z*)$_3$ (prepared according to Section 3.1, 6.51 mg, 3.00 µmol) was dissolved in 0.5 mL of acetonitrile-d$_3$ and the ^{1}H NMR spectrum was recorded.

For the switching experiment (Figure S49), irradiation (385 nm or 520 nm) was carried out for 2 min and during this time the sample was gently shaken. Following irradiation, the sample was left to stand in the dark for 1 min before the ^{1}H NMR spectrum was measured. A total of 20 irradiation cycles were carried out.

For the thermal relaxation experiment (Figure S50), the sample was irradiated with 385 nm for 2 minutes and a ^{1}H NMR spectrum was measured. The sample was kept in the dark at room temperature and ^{1}H NMR spectra were measured over time for 15.5 h.

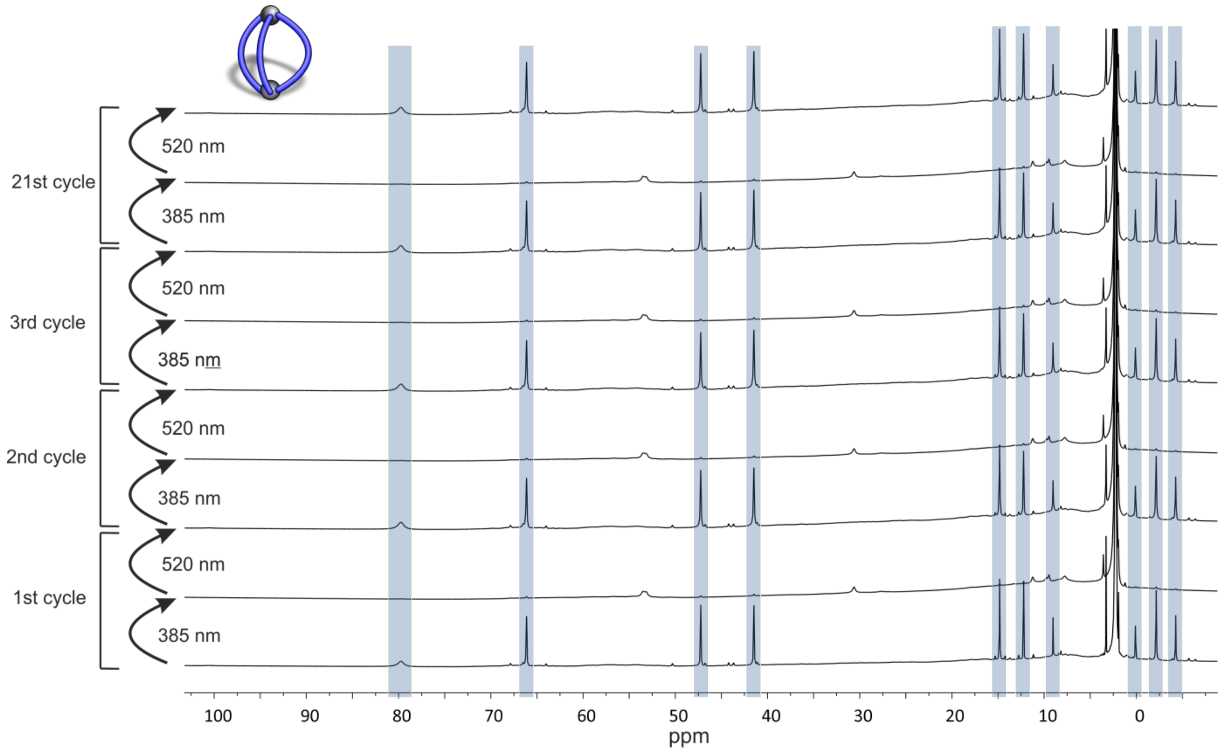

Figure S49: ^{1}H NMR spectra (600 MHz, acetonitrile-d$_3$, 298 K) of the reversible photoswitching between Co_2(**1**-*Z*)$_3$ (6 mM) and a mixture of ill-defined species for over 20 cycles with alternating irradiation of 385 nm and 520 nm for 2 mins each.

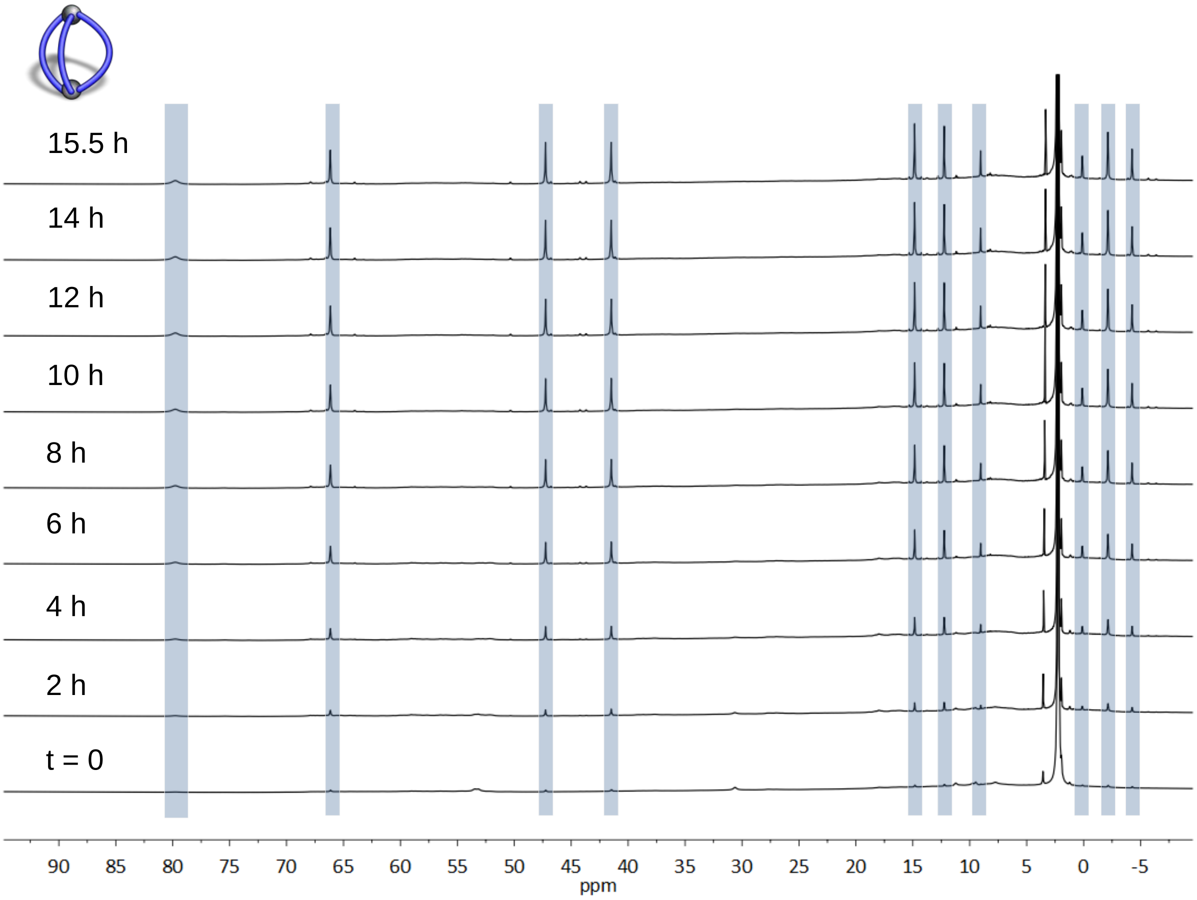

Figure S50: ^{1}H NMR spectra (600 MHz, acetonitrile-d3, 298 K) of the mixture of ill-defined species formed from irradiation of Co_2(**1**-*Z*)$_3$ with 385 nm (t = 0) at a concentration of 6 mM and thermal relaxation to Co_2(**1**-*Z*)$_3$ (blue boxes) over time.

Helicate Co_2(**1**-*Z*)$_3$ is stable at the concentration used for the UV/vis measurements (0.33 mM) since similar ^{1}H NMR spectra are obtained for 0.33 mM (0.36 mg helicate in 0.5 mL acetonitrile-d_3) and 6 mM (6.51 mg helicate in 0.5 mL acetonitrile-d_3) samples (Figure S51).

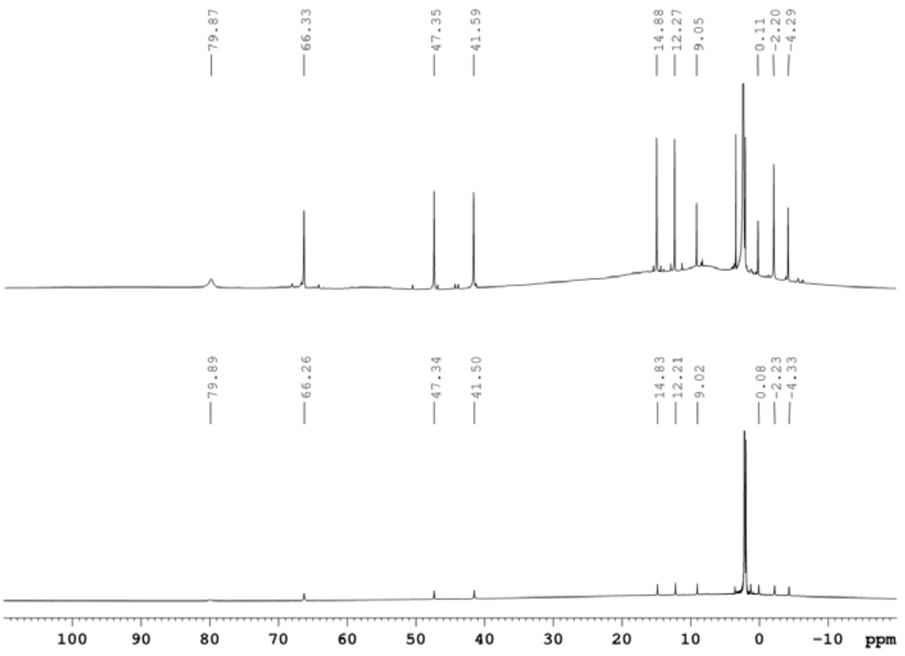

Figure S51: ^{1}H NMR spectra (600 MHz, acetonitrile-d$_3$, 298 K) of Co_2(**1**-*Z*)$_3$ at concentrations of 0.33 mM (bottom) and 6 mM (top).

5.1.2 UV/vis measurements

Following conversion of ligand **1** to its metastable *E*-form by irradiation with 385 nm for 2 min, UV/vis relaxation measurements (acetonitrile, 298.15 K, ΔT = 10 min) and fitting ln(A/A_0) at 492 nm as a function of time gave the half-life time ($t_{1/2}$) of compound **1** in the presence of Co(II) ions as 1.6 h (Figures S52-53). Furthermore, photoswitchable ligand **1** is stable over multiple switching cycles in the presence of Co(II) ions, as shown by the absorbance of a solution of Co_2(**1**-*Z*)$_3$ (MeCN, 298 K, 0.33 mM) was measured at 404 nm (black) and 491 nm (red) after alternating irradiation at 385 nm and 520 nm (Figure S54).

General procedure:

The UV/vis experiments were carried out using similar procedures to those in Section 5.1.1 but at a concentration of 0.33 mM (2.17 mg of Co_2(**1**-*Z*)$_3$ was dissolved in 3 mL acetonitrile).

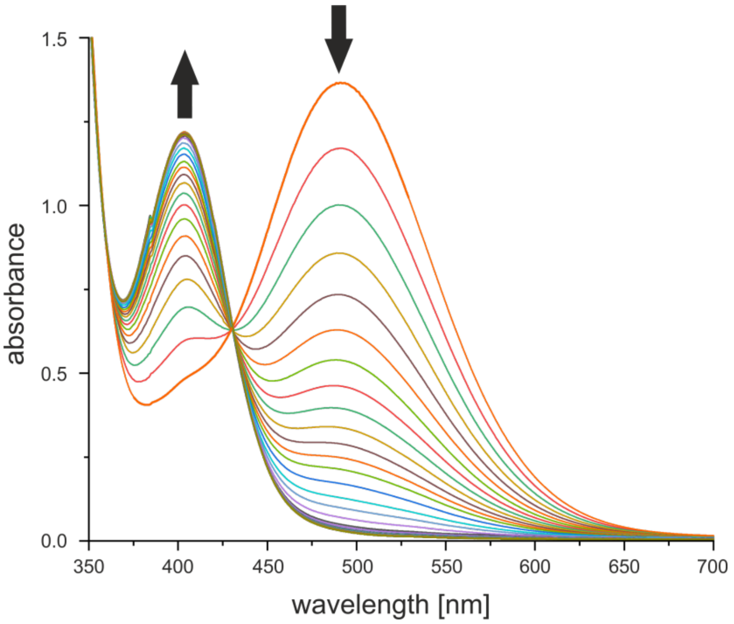

Figure S52: UV/vis relaxation experiment (MeCN, 298 K) following irradiation of helicate $Co_2(\mathbf{1}\text{-}Z)_3$ (0.33 mM) with 385 nm for 2 min.

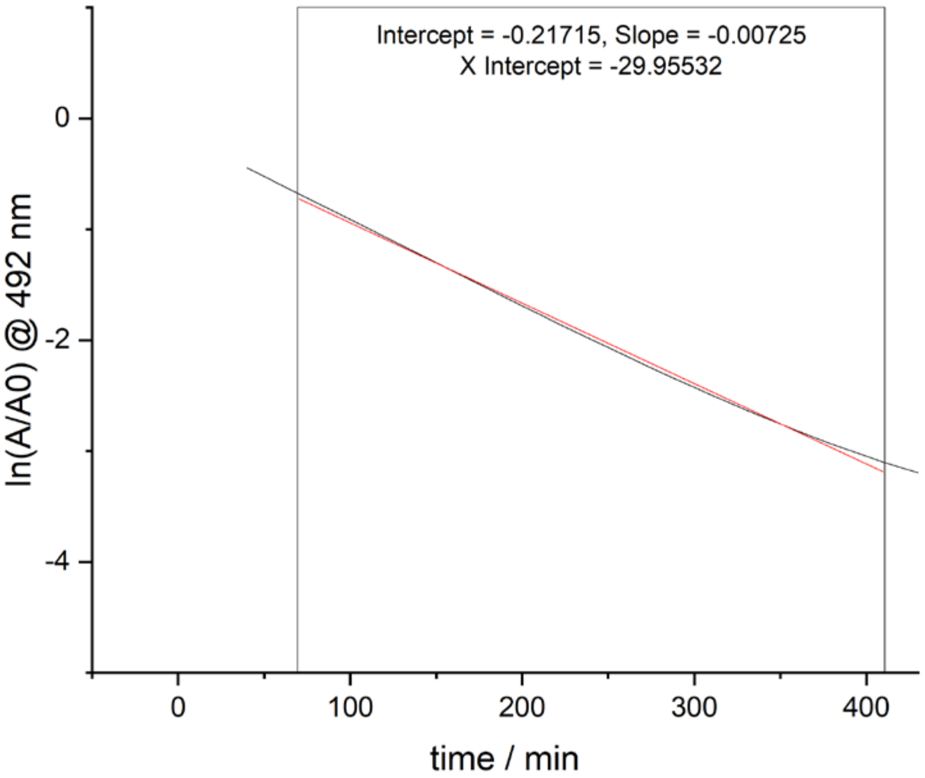

Figure S53: Plot of $\ln(A/A_0)$ measured at 492 nm of ligand **1** in the presence of Co(II) ions as a function of time and linear fitting.

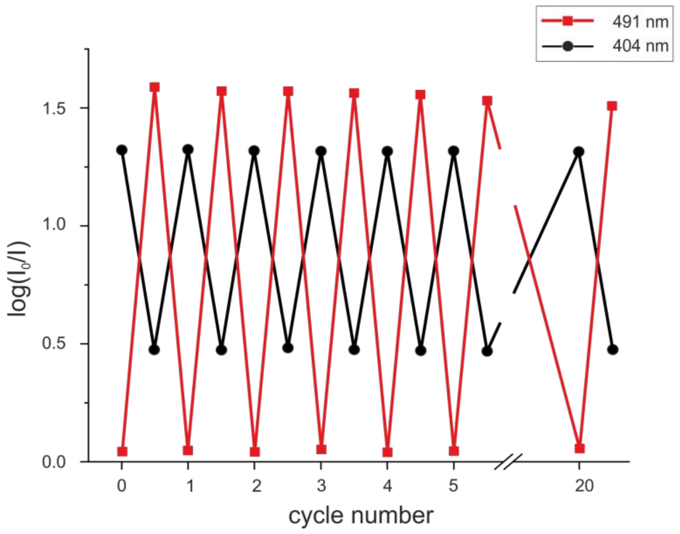

Figure S54: Absorbance at 404 nm and 491 nm of the solution (MeCN, 298 K) prepared from helicate Co_2(**1**-*Z*)$_3$ (0.33 mM) after alternating irradiation at 385 nm and 520 nm.

5.2 Self-assemblies with ligand 2

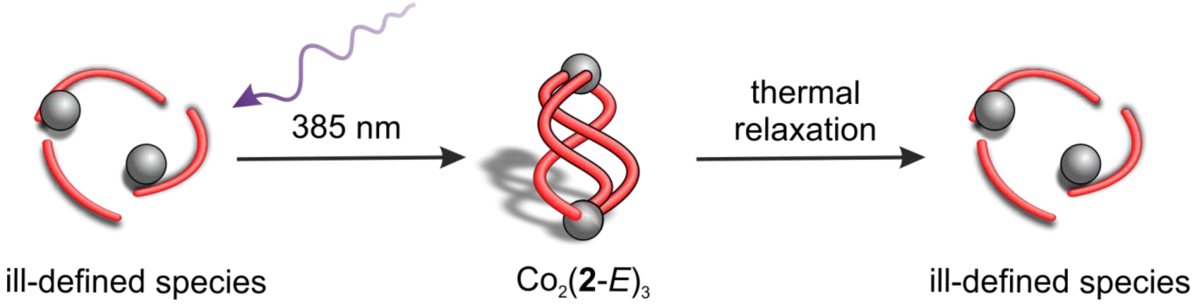

5.2.1 NMR measurements

Reversible photoswitching between a mixture of ill-defined species (formed from ligand **2**-*Z* and Co(II) ions) and Co_2(**2**-*E*)$_3$ was possible over 20 cycles (Figure S55) and Co_2(**2**-*E*)$_3$ was observed to convert to the mixture of ill-defined species following thermal relaxation (Figure S56).

General procedure:

Co_2(**2**-*E*)$_3$ (prepared according to Section 3.3, 6.51 mg, 3.00 µmol) was dissolved in 0.5 mL of acetonitrile-d_3 and the ^{1}H NMR spectrum was recorded.

For the switching experiment (Figure S55), irradiation (initially 520 nm to generate the mixture of ill-defined species for the start of the 1st cycle then alternating 385 nm or 520 nm) was carried out for 2 min and during this time the sample was gently shaken. Following irradiation, the sample was left to stand in the dark for 1 min before the ^{1}H NMR spectrum was measured. A total of 20 irradiation cycles were carried out.

For the thermal relaxation experiment (Figure S56), the sample was irradiated with 385 nm for 2 minutes to ensure the PSS was reached and a ^{1}H NMR spectrum was measured (t=0). The sample was kept in the dark at room temperature and ^{1}H NMR spectra were measured over time for 16 h.

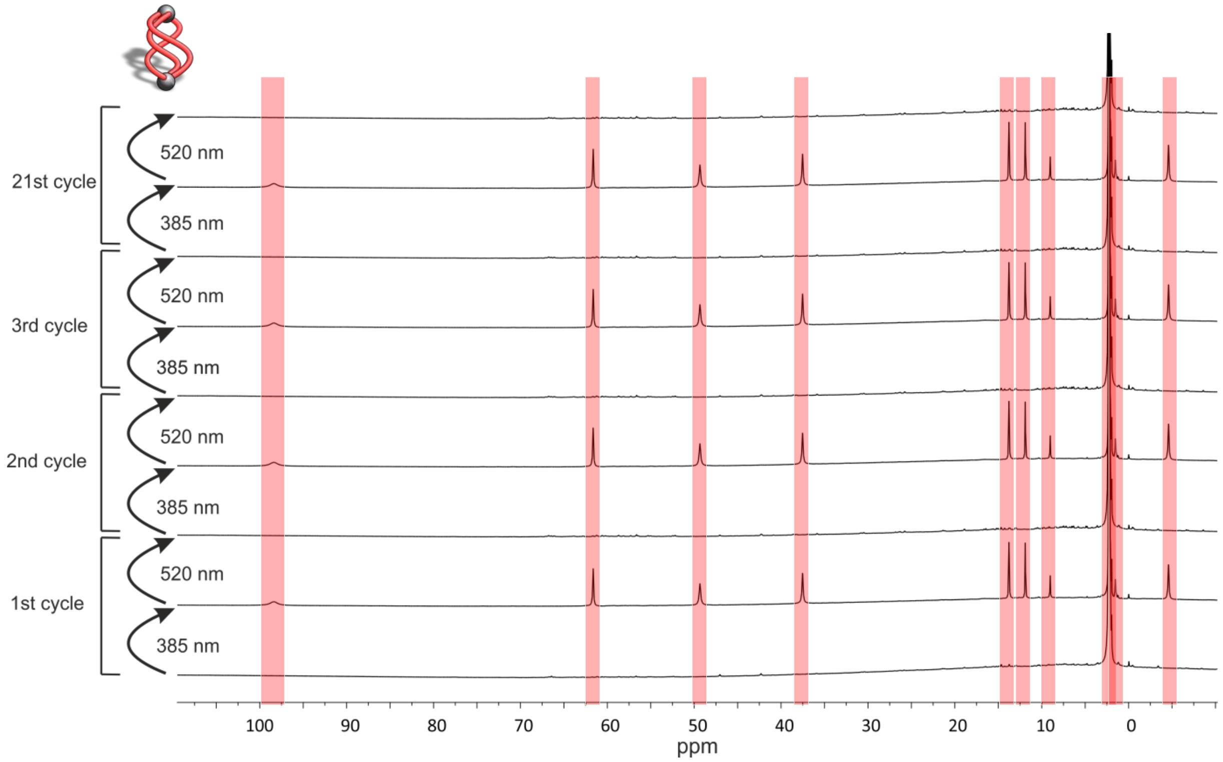

Figure S55: ^{1}H NMR spectra (600 MHz, acetonitrile-d_3, 298 K) of the reversible photoswitching between the mixture of ill-defined species and Co_2(**2**-*E*)$_3$ (6 mM) with alternating irradiation with 385 nm and 520 nm for 2 mins each, for a total of 20 cycles.

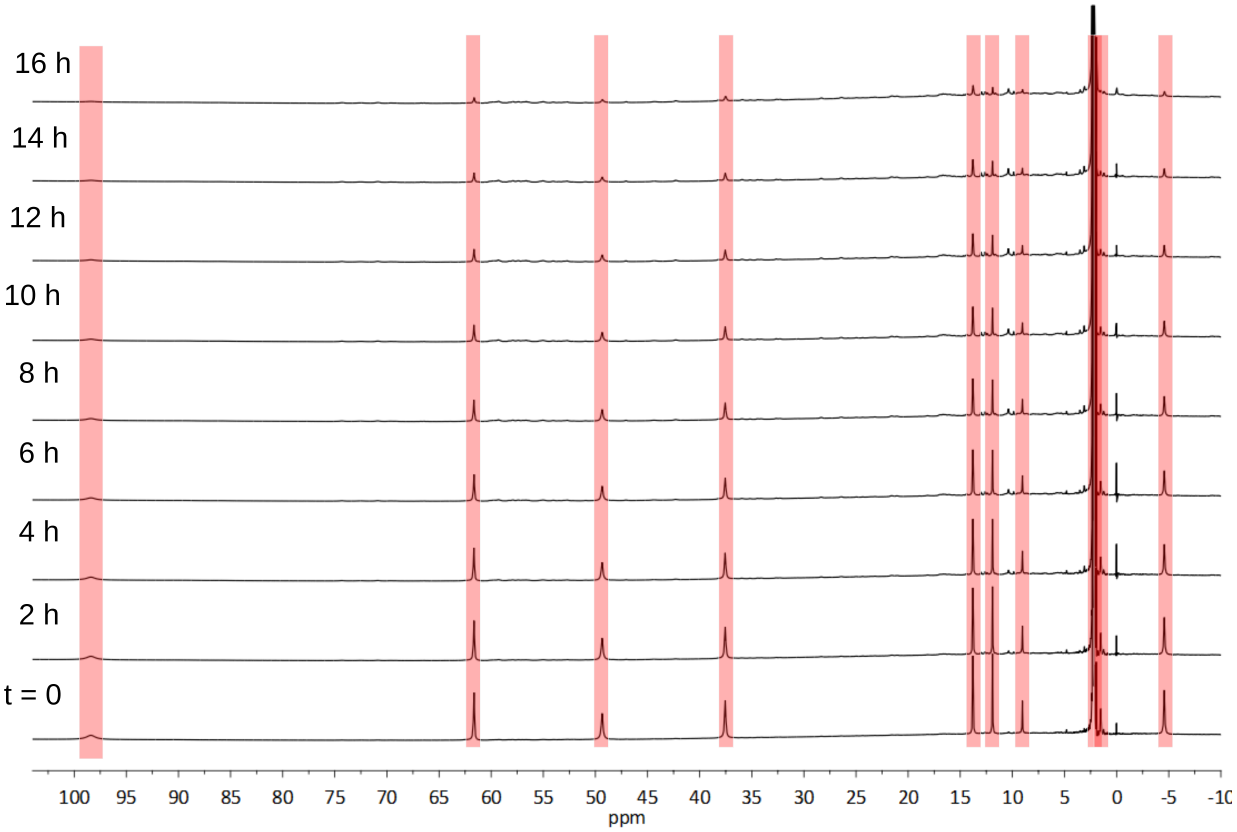

Figure S56: ^{1}H NMR spectra (600 MHz, acetonitrile-d_3, 298 K) of Co_2(**2**-*E*)$_3$ at a concentration of 6 mM after irradiation with 385 nm, the first spectrum is shown at the bottom.

Helicate Co_2(**2**-*E*)$_3$ is stable at the concentration used for the UV/vis measurements (0.33 mM) since similar ^{1}H NMR spectra are obtained for 0.33 mM (0.36 mg helicate in 0.5 mL acetonitrile-d_3) and 6 mM (6.51 mg helicate in 0.5 mL acetonitrile-d_3) samples (Figure S57).

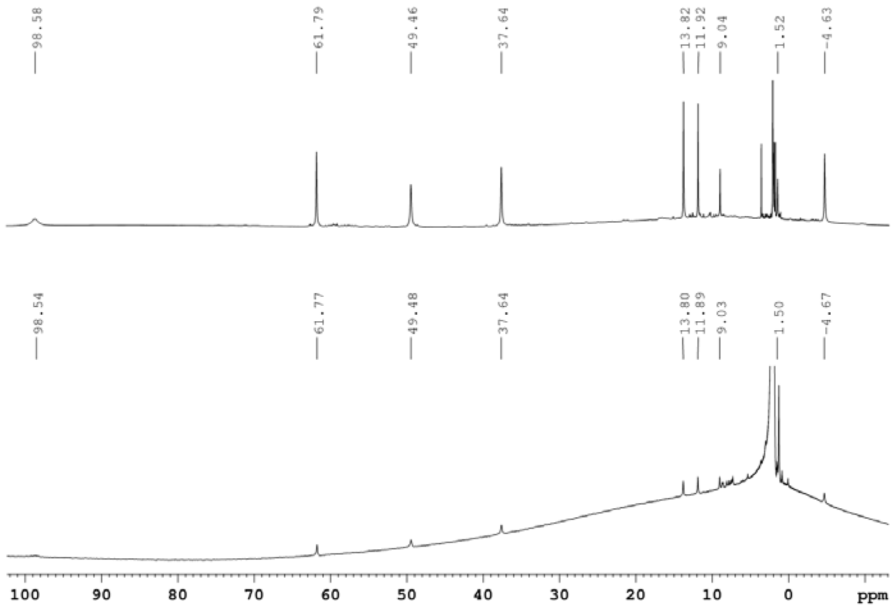

Figure S57: ^{1}H NMR spectra (600 MHz, acetonitrile-d_3, 298 K) of Co_2(**2**-*E*)$_3$ at a concentration of 0.33 mM (bottom) and 6 mM (top).

5.2.2 UV/vis measurements

Following conversion of ligand **2** to its metastable *E*-form by irradiation with 385 nm for 2 min, UV/vis relaxation measurements (acetonitrile, 298.15 K, ΔT = 10 min) and fitting $\ln(A/A_0)$ at 484 nm as a function of time gave the half-life time ($t_{1/2}$) of compound **2** in the presence of Co(II) ions as 9.2 h (Figures S58-59). Furthermore, photoswitchable ligand **2** is stable over multiple switching cycles in the presence of Co(II) ions, as shown by the absorbance at 397 nm (black) and 483 nm (red) after alternating irradiation at 385 nm and 520 nm.

General procedure:

The UV/vis experiments were carried out using similar procedures to those in Section 5.2.1 but at a concentration of 0.33 mM (2.17 mg of Co_2(**2**-*E*)$_3$ was dissolved in 3 mL acetonitrile) and the sample was irradiated with 385 nm for 2 minutes to ensure the PSS was reached.

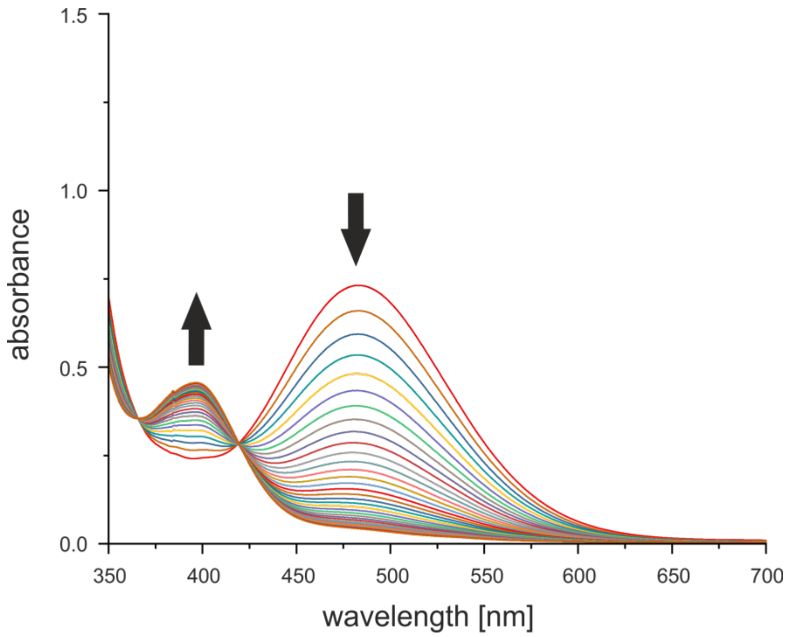

Figure S58: UV/vis relaxation experiment of helicate Co_2(**2**-*E*)$_3$ (MeCN, 298 K, 0.33 mM) after irradiation with 385 nm for 2 min.

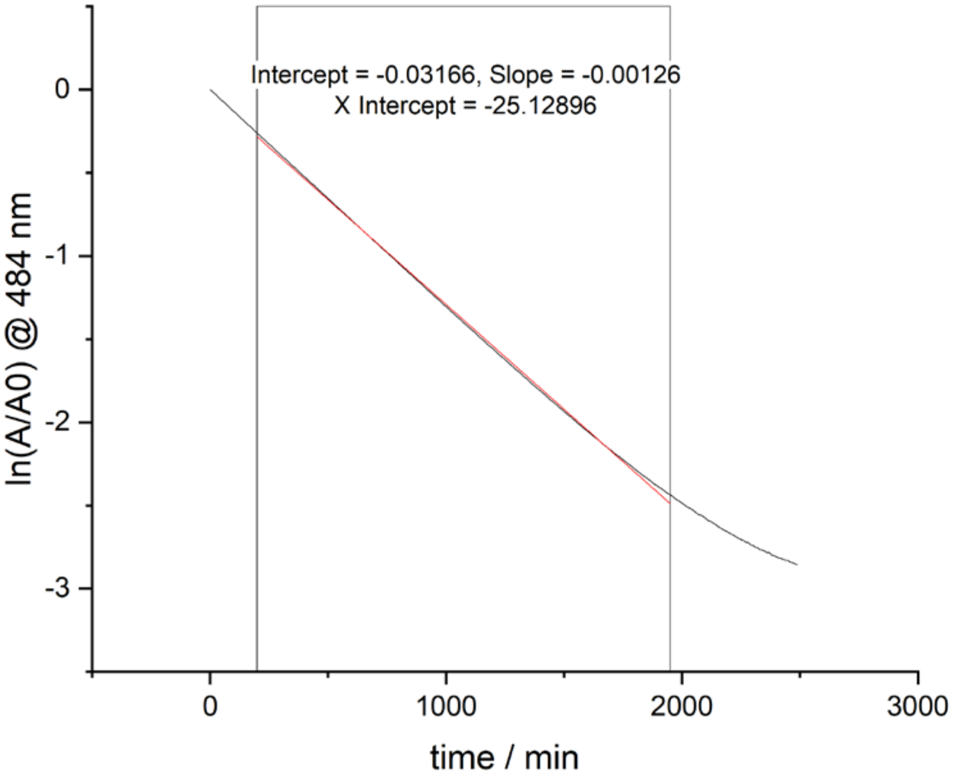

Figure S59: Plot of ln(A/A_0) measured at 484 nm of ligand **2** in the presence of Co(II) ions as a function of time and linear fitting.

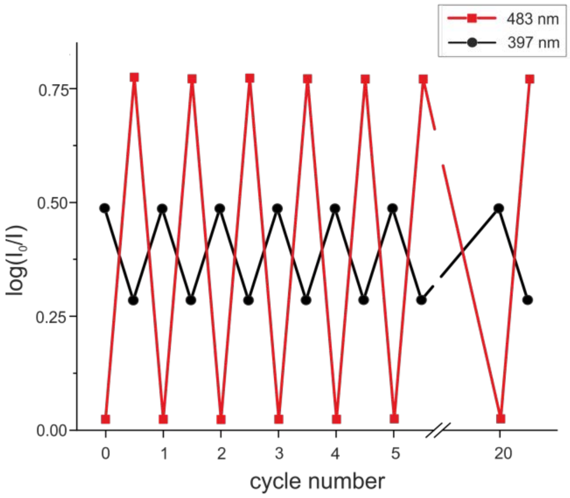

Figure S60: Absorbance of a solution of Co_2(**2**-*E*)$_3$ (MeCN, 298 K, 0.33 mM) at 397 nm and 483 nm after alternating irradiation at 385 nm and 520 nm.

6. Ligand Competition Experiments

6.1 Helicate Stability Experiments

In order to compare the relative stability of the two helicates Co_2(**1**-*Z*)$_3$ and Co_2(**2**-*E*)$_3$, competition experiments were carried out where a competing ligand (**1**-*Z* or **2**-*E*, respectively) was added to a solution containing the helicate and changes to the ^{1}H NMR spectra were monitored over time.

6.1.1 Addition of 2-*E* to Co$_2$(1-*Z*)$_3$

Following addition of 3 equivalents of **2**-*E* to the preformed helicate Co_2(**1**-*Z*)$_3$, signals corresponding to Co_2(**2**-*E*)$_3$ appeared in the ^{1}H NMR spectrum (Figures S61 and S62). Time course NMR measurements revealed that these signals decrease over time due to thermal relaxation of Co_2(**2**-*E*)$_3$, while those for the Co_2(**1**-*Z*)$_3$ helicate remained. Ill-defined species are also proposed to form following addition of **2**-*E* and during thermal relaxation.

Procedure:
Ligand **2**-*E* was obtained by dissolving **2**-*Z* (1.49 mg, 3.00 µmol) in 2 mL of dichloromethane and the solution was irradiated with 385 nm light for 5 min before removal of the solvent by a nitrogen stream.

Co_2(**1**-*Z*)$_3$ (prepared according to Section 3.1, 6.51 mg, 3.00 µmol) was dissolved in 0.5 mL of acetonitrile-d$_3$ and the ^{1}H NMR spectrum was measured. 3 Equivalents of ligand **2**-*E* (prepared above) relative to the helicate were added to the solution of Co_2(**1**-*Z*)$_3$ and NMR spectra were measured over 17.5 hours.

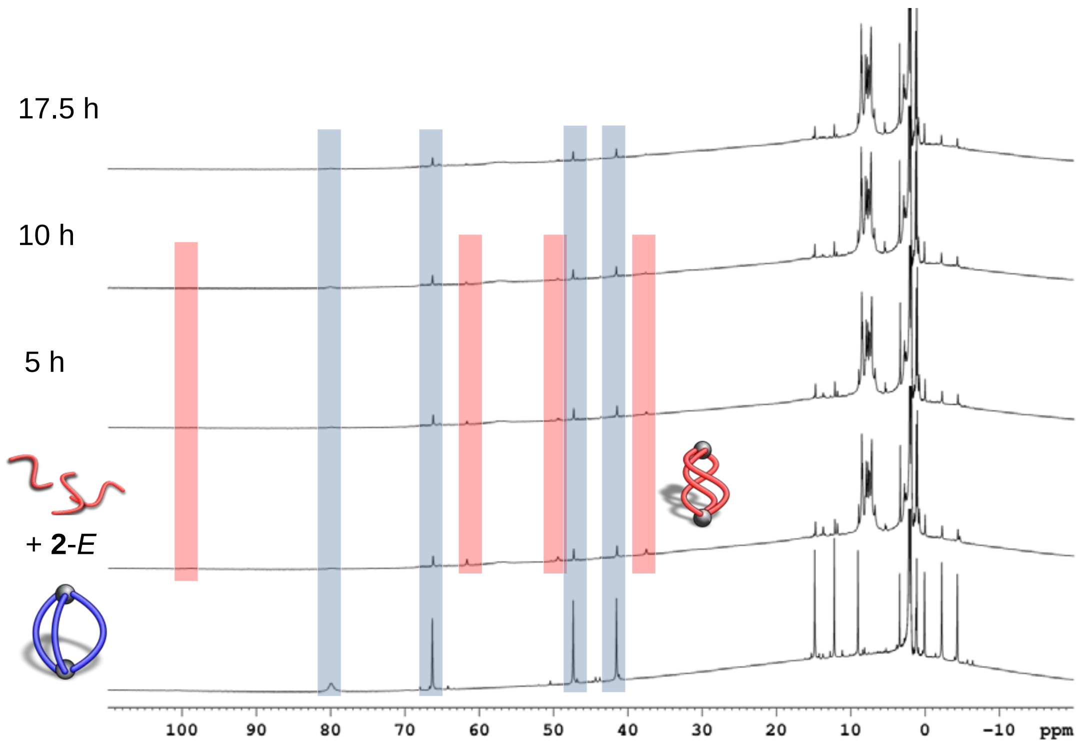

Figure S61: ^{1}H NMR spectra (500 MHz, acetonitrile-d3, 298 K) of pre-formed helicate Co_2(**1**-*Z*)$_3$ (bottom spectrum, blue boxes, 6 mM), following addition of ligand **2**-*E* and over time showing the appearance and disappearance of Co_2(**2**-*E*)$_3$ (red boxes) due to thermal relaxation. For clarity, signals below 30 ppm are not assigned with colored boxes.

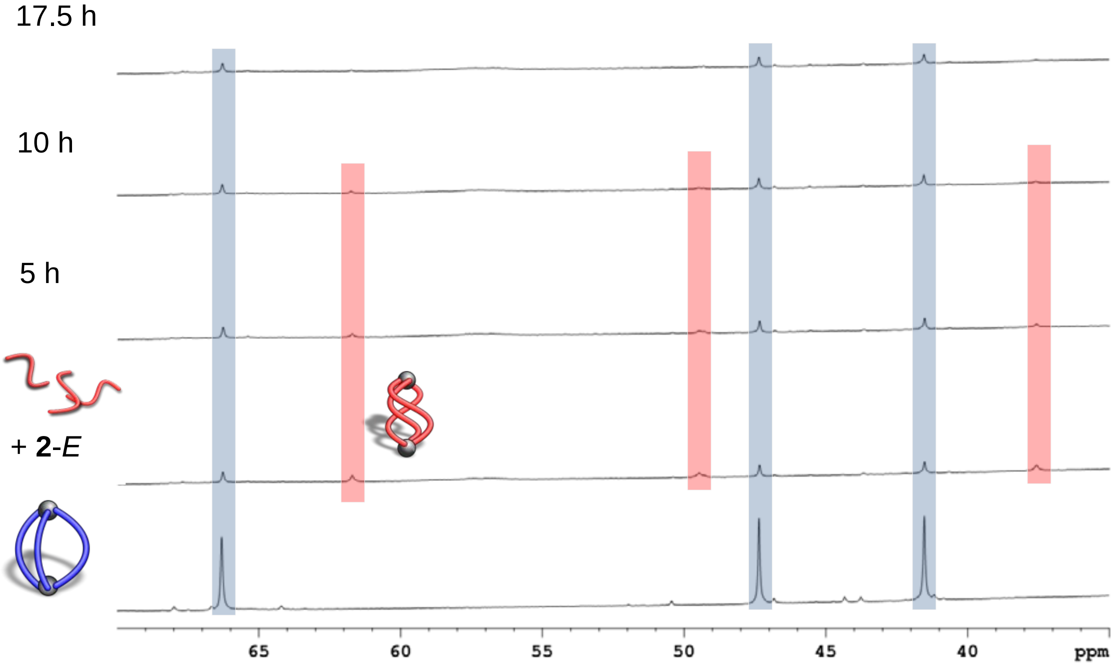

Figure S62: Zoomed region of the ^{1}H NMR spectra (500 MHz, acetonitrile-d3, 298 K) in Figure S61 of pre-formed helicate Co_2(**1**-*Z*)$_3$ (bottom spectrum, blue boxes, 6 mM) and following the addition of **2**-*E*.

6.1.2 Addition of 1-*Z* to Co_2(2-*E*)$_3$

Addition of **1**-*Z* to Co_2(**2**-*E*)$_3$ resulted in the formation of a small amount of helicate Co_2(**1**-*Z*)$_3$, indicating that **1**-*Z* is competitive with **2**-*E* for the cobalt ions, although Co_2(**2**-*E*)$_3$ was still the major species (Figures S63 and S64). Over time, as ligand **2**-*E* relaxes, Co_2(**2**-*E*)$_3$ is degraded and the amount of Co_2(**1**-*Z*)$_3$ increases.

Procedure:
Co_2(**2**-*E*)$_3$ (prepared according to Section 3.3, 7.60 mg, 3.50 µmol) was dissolved in 0.5 mL of acetonitrile-d$_3$ and the ^{1}H NMR spectrum was measured. 3 Equivalents of ligand **1**-*Z* (1.74 mg, 3.50 µmol) relative to the helicate were added to the solution of Co_2(**1**-*Z*)$_3$ and NMR spectra were measured over 17.5 hours.

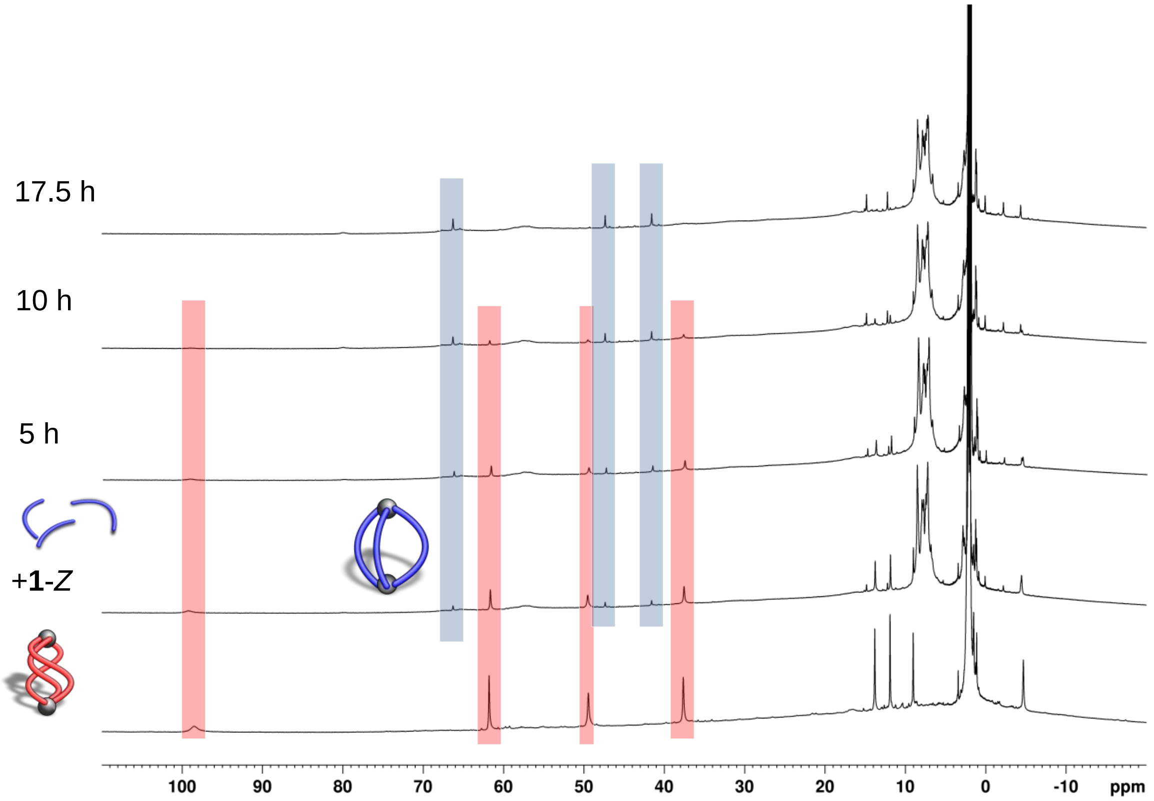

Figure S63: ^{1}H NMR spectra (500 MHz, acetonitrile-d$_3$, 298 K) of pre-formed helicate Co_2(**2**-*E*)$_3$ (bottom spectrum, red boxes, 7 mM), following addition of ligand **1**-*Z* and over time showing the appearance of Co_2(**1**-*Z*)$_3$ (blue boxes). For clarity, signals below 30 ppm are not assigned with colored boxes.

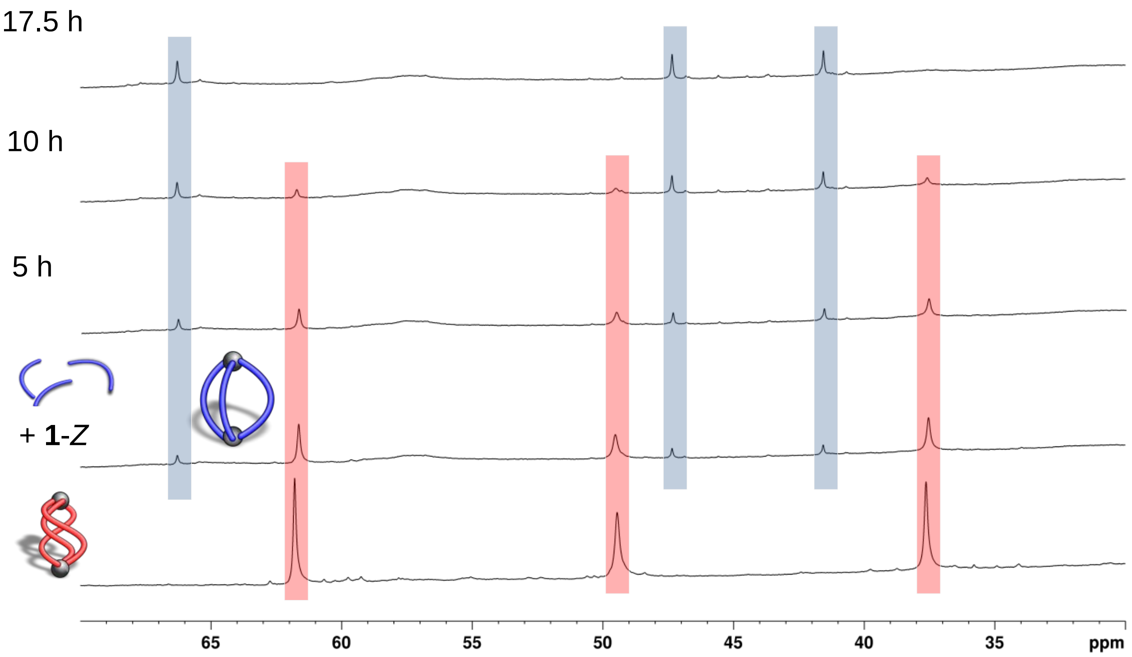

Figure S64: Zoomed region of the ^{1}H NMR spectra (500 MHz, acetonitrile-d$_3$, 298 K, 7 mM) in Figure S63 of pre-formed helicate Co_2(**2**-*E*)$_3$ (bottom spectrum, red boxes, 7 mM) and following the addition of **1**-*Z*.

6.2 Light-Controlled Assembly/Disassembly of Co_2(1-*Z*)$_3$ and Co_2(2-*E*)$_3$

Switching between the two helicates, Co_2(**1**-*Z*)$_3$ and Co_2(**2**-*E*)$_3$, is induced in the ligand competition experiment by alternating irradiation of 385 nm and 520 nm light and is fully reversible (Figure S65). Irradiation with 385 nm disassembles Co_2(**1**-*Z*)$_3$ (blue boxes) and assembles Co_2(**2**-*E*)$_3$ (red boxes), whereas irradiation with 520 nm disassembles Co_2(**2**-*E*)$_3$ (red boxes) and assembles Co_2(**1**-*Z*)$_3$ (blue boxes).

Procedure:
Co_2(**1**-*Z*)$_3$ (prepared according to Section 3.1, 6.51 mg, 3.00 µmol) was dissolved in 0.5 mL of acetonitrile-d$_3$ and 3 equivalents of ligand **2**-*Z* (1.49 mg, 3.00 µmol) relative to the helicate were added before measuring the ^{1}H NMR spectrum. For switching, irradiation (385 nm or 520 nm) was carried out for 2 min and during this time the sample was gently shaken. Following irradiation, the sample was left to stand in the dark for 2 min before the ^{1}H NMR measurement was carried out.

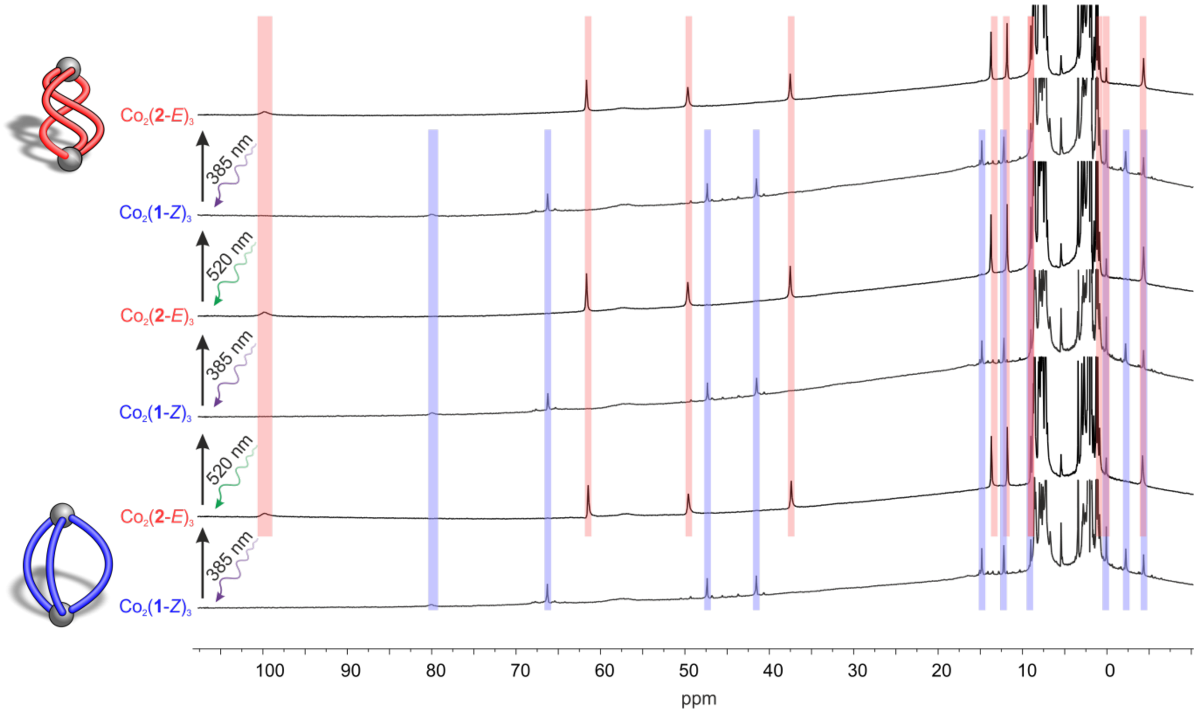

Figure S65: ^{1}H NMR spectra (500 MHz, acetonitrile-d$_3$, 298 K) of the ligand competition experiment showing the formation of either helicate Co_2(**1**-*Z*)$_3$ (blue boxes) or Co_2(**2**-*E*)$_3$ (red boxes) induced by alternating irradiation with 385 nm and 520 nm.

In a related ligand competition experiment, the thermal relaxation from helicate Co_2(**2**-*E*)$_3$ back to Co_2(**1**-*Z*)$_3$ was measured over time at room temperature and under exclusion of light (Figure S66).

Procedure:

Co_2(**1**-*Z*)$_3$ (prepared according to Section 3.1, 5.43 mg, 2.50 µmol) was dissolved in 0.5 mL of acetonitrile-d$_3$ and 3 equivalents of ligand **2**-*Z* (1.24 mg, 2.50 µmol) relative to the helicate were added. The sample was irradiated with 385 nm for 2 minutes and a ^{1}H NMR spectrum was measured. The sample was kept in the dark at room temperature and ^{1}H NMR spectra were measured over time.

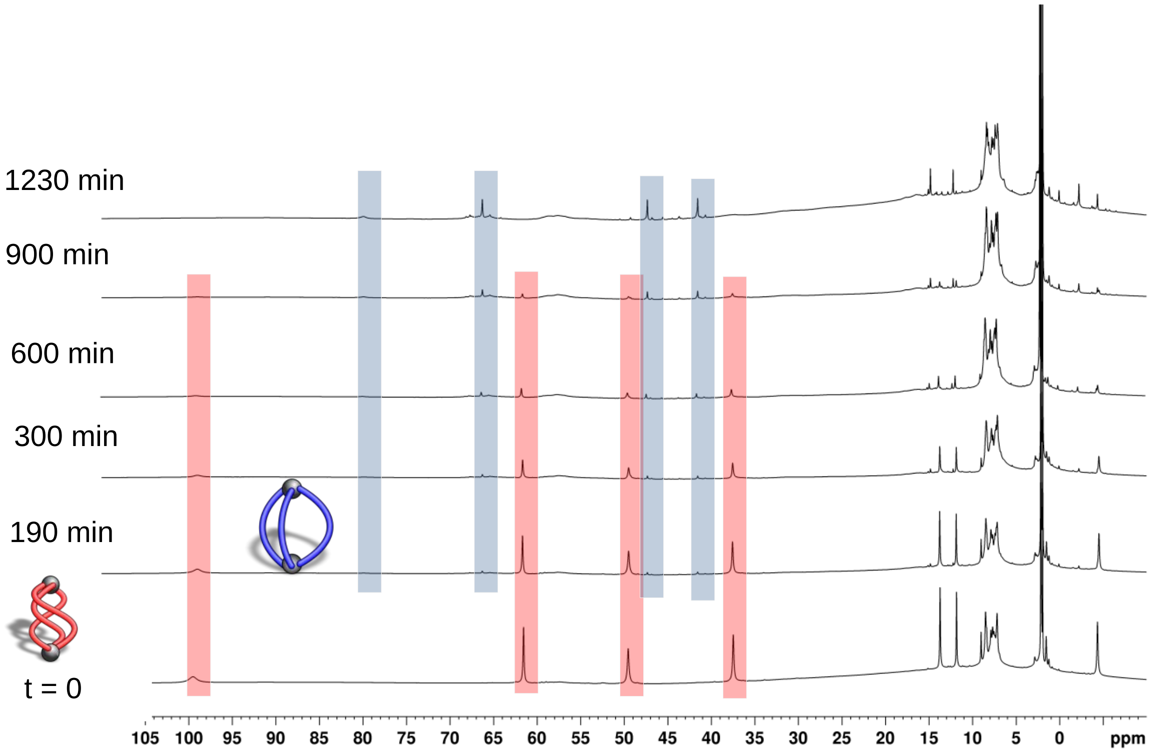

Figure S66: ^{1}H NMR spectra (600 MHz, acetonitrile-d$_3$, 298 K) of the ligand competition experiment after irradiation with 385 nm for 2 min showing the transformation from Co_2(**2**-*E*)$_3$ (red boxes) to Co_2(**1**-*Z*)$_3$ (blue boxes) over time due to thermal relaxation. For clarity, signals below 30 ppm are not assigned with colored boxes.

7. Quantum mechanical calculations

All structures were obtained from a series of geometry optimizations. Pre-optimizations were caried out with xtb[6] and the GFN2-xtb method.[7] The resulting structures were refined in ORCA[8] using a ωB97X-D3[6]/def2-SVP[9] level of theory with Grimme's Dispersion correction[10] as well as an auxiliary basis set[11] and the ROJCOSX approximation for Coulomb and HF Exchange.[12] The structures were then further refined using a ωB97X-D3/def2-TZVP level of theory.

The bite angle (ϴ) of the diazocine ligands was calculated from two straight lines given by the coordinates of the carbon atom in the diazocine phenyl ring carrying the nitrogen substituent (4, 4'- for **2**-*Z* and **2**-*E*, 3, 3'- for **1**-*Z* and **1**-*E*) and the neighboring nitrogen atom (see Figure S67: $\overrightarrow{N_1C_1}$ and $\overrightarrow{N_2C_2}$). The minimal distance between the two lines (given by P_1 and P_2) was calculated and a center point (M) equidistant and perpendicular to both straight lines was obtained. The bite angles were calculated from the nitrogen atoms on either side of the diazocine (N_1 and N_2) and the center point (M) between the initial straight lines (angle between $\overrightarrow{N_1M}$ and $\overrightarrow{N_2M}$). The bite angle for the free **2**-*Z* ligand is 63°, while a bite angle of 137° was obtained for the freely optimized **1**-*E*.

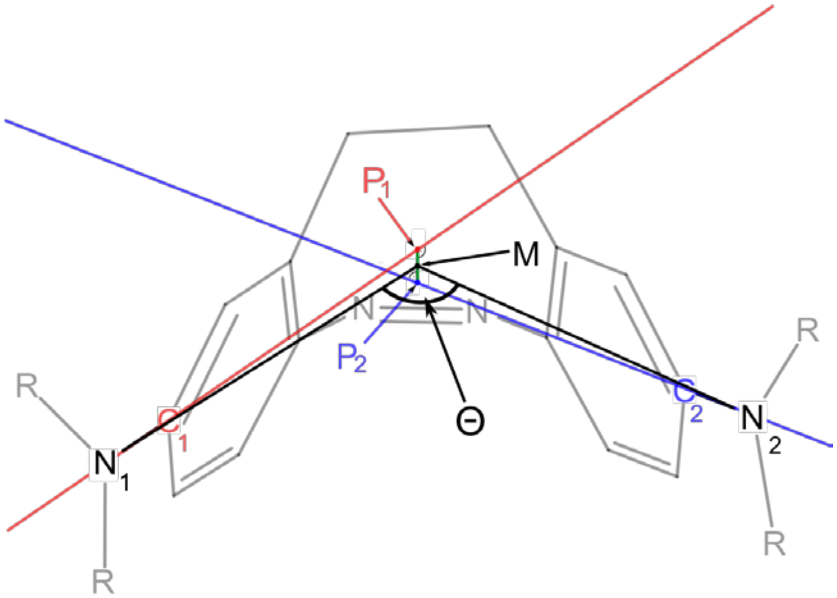

Figure S67: Schematic representation of the method used to determine the bite angle Θ. Two straight lines are given by the vectors $\overrightarrow{N_1C_1}$ and $\overrightarrow{N_2C_2}$ (blue and red). The line $\overline{P_1P_2}$ represents the minimal distance between the two straight lines and is perpendicular to both initial vectors. M is the center of $\overline{P_1P_2}$. Θ is defined as the angle between the vectors $\overrightarrow{N_1M}$ and $\overrightarrow{N_2M}$. which corresponds to the projection of $\overrightarrow{N_1C_1}$ and $\overrightarrow{N_2C_2}$ onto the N_1MN_2 plane.

A comparison of the total energies of the experimentally observed self-assemblies (Co_2(**1**-*Z*)$_3$ and Co_2(**2**-*E*)$_3$) as the mesocate (ΔΛ) and helicate (ΛΛ) indicates the helicates are lower in energy compared to the mesocate (Table S4). Thus, Co_2(**1**-*Z*)$_3$ and Co_2(**2**-*E*)$_3$ are proposed to be helicates.

Table S4: Total energy of the Co_2(**1**-*Z*)$_3$ and Co_2(**2**-*E*)$_3$ self-assemblies as mesocates (ΔΛ) and helicates (ΛΛ).

Self-Assembly	Total Energy (Hartree)	Relative Energy (kcal/mol)
ΔΛ-Co_2(**1**-*Z*)$_3$	-7644.493636020295	1.22
ΛΛ-Co_2(**1**-*Z*)$_3$	-7644.495581080593	0
ΔΛ-Co_2(**2**-*E*)$_3$	-7644.491614934993	1.77
ΛΛ-Co_2(**2**-*E*)$_3$	-7644.494445751221	0

Strain energies (E_{strain}) for the proposed helicates were obtained comparing the total energy of a freely optimized ligand with a ligand from the proposed helicate structures (Table S5). The calculations predict less strain energy within the respective helicates for **1**-Z and **2**-E when compared to **2**-Z and **1**-*E* (Table S6). This suggests Co_2(**1**-*Z*)$_3$ and Co_2(**2**-*E*)$_3$ are the less strained helicates.

$$\boldsymbol{E_{strain}} = \sum \boldsymbol{E_{Co_2(L)_3}} - \boldsymbol{E_{L_{free}}}$$

Table S5: Total energies for a single ligand calculated on the ωB97X-D3/def2-TZVP level of theory. The energies for ligands Co_2(**L**)$_3$ were obtained by a single point calculation for the ligand as present in the proposed helicate.

Ligand	Total Energy of a Single Ligand (Hartree)	Bite Angle Θ in °
**1**-*E* (free optimization)	-1626.504907121961	137
**1**-*Z* (free optimization)	-1626.518811203468	61
**2**-*E* (free optimization)	-1626.504426802891	110
**2**-*Z* (free optimization)	-1626.517445797561	63
**1**-*E* (in Co_2(**1**-*E*)$_3$)	-1626.504907121961	125
**1**-*Z* (in Co_2(**1**-*Z*)$_3$)	-1626.518811203468	81
**2**-*E* (in Co_2(**2**-*E*)$_3$)	-1626.504426802891	110
**2**-*Z* (in Co_2(**2**-*Z*)$_3$)	-1626.517445797561	82

Table S6: Strain energy per ligand in Co_2(**L**)$_3$.

Ligand	E_{strain} (Hartree)	E_{strain} (kcal/mol)
**1**-*E*	0.015315495872982865	9.61
**1**-*Z*	0.013784665178945943	8.65
**2**-*E*	0.01172895116997097	7.36
**2**-*Z*	0.015175018660102069	9.52

The properties of the molecular cavity and windows was analyzed using *py*window.[13] *py*window estimates the volume of the cavity (V_{void}) by fitting a sphere with maximum diameter (D_{void}) inside a given structure. The calculated pore diameter corresponds to the diameter of the largest sphere (D_{wind}) that fits into that window.

Table S7: Properties of the molecular cavities and pores of Co_2(**2**-*E*)$_3$ and Co_2(**1**-*Z*)$_3$.

Helicate	n_{wind}	$D_{wind,1}$ (Å)	$D_{wind,2}$ (Å)	$D_{wind,3}$ (Å)	D_{void} (Å)	V_{void} (Å^3)
Co_2(**2**-*E*)$_3$	3	3.84	3.08	3.31	4.63	51.90
Co_2(**1**-*Z*)$_3$	3	5.30	5.32	4.97	6.51	144.58

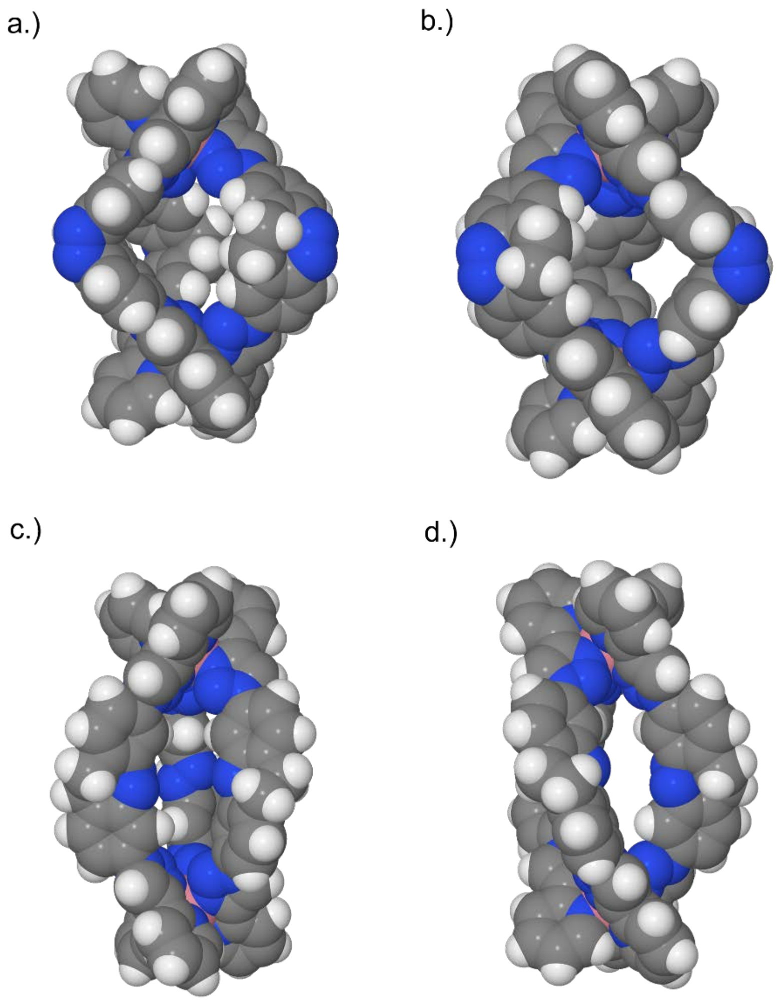

Figure S68: Van der Waals representation of the optimized structures for helicates and mesocates of Co_2(**1**-*Z*)$_3$ and Co_2(**2**-*E*)$_3$. a) ΔΛ-Co_2(**1**-*Z*)$_3$ b) ΛΛ-Co_2(**1**-*Z*)$_3$ c) ΔΛ-Co_2(**2**-*E*)$_3$ d) ΛΛ-Co_2(**2**-*E*)$_3$.

7.1 Xyz files for the ligands

7.1.1 1-*E* (in Co_2(1-*E*)$_3$)

58
ENERGY = -7644.491169817798 Input = wB97X-D3 def2-TZVP def2/J D3ZERO RIJCOSX OPT

H	-3.60210	-15.74290	7.58230
C	-2.81030	-15.26030	8.13860
H	-1.27850	-16.67290	7.61030
C	-1.52280	-15.77090	8.15690
C	-3.07530	-14.11010	8.85420
C	-0.55120	-15.10980	8.88650
H	-4.06650	-13.67660	8.86570
N	-2.14510	-13.46720	9.56550
H	0.46220	-15.48670	8.92610
C	-0.89090	-13.95910	9.57890
C	0.06930	-13.17990	10.36000
C	1.43670	-13.15110	10.45040
H	2.22380	-13.72500	9.99180
N	-0.40220	-12.17530	11.13300
N	1.69040	-12.11480	11.27640
N	0.56660	-11.53240	11.68170
H	2.43130	-9.65110	10.88890
C	3.20120	-10.23550	11.37990
C	5.39350	-7.29610	12.43840

H	4.93970	-6.30490	12.36030
C	2.95800	-11.57320	11.65360
N	6.51700	-9.78990	12.75150
C	7.80820	-8.12800	12.11110
C	4.41900	-9.66640	11.71350
N	7.14610	-9.34010	11.79160
C	9.17420	-7.94090	12.03870
C	6.90350	-7.08120	12.35220
H	9.82510	-8.77780	11.82250
C	3.89290	-12.37750	12.27760
C	9.69380	-6.67390	12.25280
C	5.36260	-10.50030	12.33340
C	7.44180	-5.81830	12.53590
H	3.66870	-13.41210	12.50450
C	5.11030	-11.82320	12.63770
C	8.81640	-5.63460	12.49330
H	10.76100	-6.49740	12.20690
C	4.69280	-8.20060	11.38380
H	6.80050	-4.96510	12.72940
N	9.31530	-4.30460	12.68440
N	9.00350	-3.36140	11.81040
H	5.86000	-12.41320	13.14830
C	10.01370	-3.80790	13.72950
H	10.33020	-4.42430	14.55350
N	9.49040	-2.26590	12.27660
C	10.12210	-2.46990	13.45570
C	10.63810	-1.27330	14.11320
C	11.37350	-1.27170	15.28290
H	11.62130	-2.20410	15.77260
N	10.30880	-0.13610	13.46530
C	11.78850	-0.05930	15.80930
C	10.70920	1.02480	13.98410
C	11.44940	1.10880	15.14890
H	12.36910	-0.02940	16.72270
H	10.42330	1.91820	13.44510
H	11.75250	2.07640	15.52460
H	5.24010	-8.14950	10.43970
H	3.71720	-7.75160	11.18270
H	5.14570	-7.65030	13.44180

7.1.2 1-*Z* (in Co_2(1-*Z*)$_3$)

58

ENERGY = -7644.495581080593 Input = wB97X-D3 def2-TZVP def2/J D3ZERO RIJCOSX OPT

H	-7.65060	-13.69980	-0.03810
C	-6.77890	-13.41590	0.53550
H	-5.36080	-14.47420	-0.68540
C	-5.50890	-13.84140	0.18070
C	-6.91860	-12.61170	1.64880
C	-4.42990	-13.44740	0.95190
H	-7.88990	-12.25380	1.96520
N	-5.88250	-12.23120	2.40130
H	-3.42610	-13.76210	0.69940
C	-4.64700	-12.64170	2.05810
C	-3.57440	-12.18820	2.94310
C	-2.23010	-12.44260	3.02770
H	-1.55640	-13.06130	2.45990
N	-3.88950	-11.36520	3.96970
N	-1.82850	-11.74800	4.11250
N	-2.84540	-11.10120	4.67430
H	0.46240	-11.21020	2.88450
C	0.58260	-11.46200	3.93240
C	3.67260	-9.83750	3.84740
H	4.69810	-9.84540	3.46700
C	-0.52920	-11.73380	4.71140
N	3.24970	-12.15070	6.37520
C	3.79070	-9.87460	6.43540

C	1.85410	-11.51970	4.48920
N	4.08060	-11.27410	6.58230
C	3.80480	-9.18170	7.64210
C	3.67310	-9.19800	5.21970
H	3.91690	-9.74160	8.56200
C	-0.41640	-12.05200	6.05480
C	3.70240	-7.80950	7.67390
C	1.95890	-11.86300	5.83740
C	3.61680	-7.80480	5.26820
H	-1.29990	-12.27540	6.63760
C	0.84260	-12.13360	6.61500
C	3.64520	-7.12550	6.46990
H	3.68610	-7.29620	8.62590
C	3.10150	-11.25830	3.69150
H	3.56310	-7.24340	4.34440
N	3.65670	-5.69260	6.48040
N	3.24660	-4.99220	5.43380
H	0.97090	-12.44180	7.64480
C	4.09100	-4.88550	7.47450
H	4.49760	-5.25890	8.39700
N	3.40510	-3.74970	5.73990
C	3.91560	-3.62090	6.98720
C	4.13900	-2.26320	7.46690
C	4.65780	-1.94380	8.70800
H	4.93270	-2.72610	9.40300
N	3.78490	-1.32310	6.56880
C	4.82060	-0.60910	9.04040
C	3.94660	-0.04100	6.89930
C	4.45760	0.35880	8.11980
H	5.22590	-0.32930	10.00460
H	3.65840	0.69020	6.15660
H	4.56970	1.41240	8.33590
H	3.85770	-11.99390	3.96850
H	2.89950	-11.41880	2.63190
H	3.11140	-9.16740	3.19240

7.1.3 2-*Z* (in Co_2(2-*Z*)$_3$)

58

ENERGY = -7644.494445751221 Input = wB97X-D3 def2-TZVP def2/J D3ZERO RIJCOSX OPT

H	-8.37100	-14.93870	0.22750
C	-7.51360	-14.60330	0.79460
H	-6.08910	-15.88230	-0.18390
C	-6.24850	-15.12100	0.56920
C	-7.66850	-13.63900	1.77030
C	-5.18780	-14.64890	1.32100
H	-8.63760	-13.20730	1.98310
N	-6.65130	-13.18470	2.50680
H	-4.18800	-15.03230	1.16680
C	-5.41810	-13.67630	2.28050
C	-4.36200	-13.10760	3.11650
C	-3.00440	-13.27670	3.19460
H	-2.30680	-13.87960	2.64000
N	-4.70180	-12.17780	4.03830
H	2.19370	-10.71080	7.97140
N	-2.61950	-12.43160	4.17360
N	-3.65990	-11.77270	4.67450
H	-1.74960	-10.13460	5.17600
C	-0.99700	-10.90910	5.13060
C	3.00230	-10.53710	7.25730
H	3.89300	-10.96350	7.71980
C	-1.29830	-12.17350	4.65260
N	0.71870	-9.45490	6.14460
C	3.25230	-9.03320	7.16090
C	0.29810	-10.66300	5.53150
N	1.60590	-8.89950	5.49140
C	4.25480	-8.43750	7.91580

C	2.50350	-8.18770	6.32750
H	4.87310	-9.05200	8.55900
C	-0.34600	-13.17690	4.63290
C	4.49740	-7.07280	7.85890
C	1.30570	-11.63910	5.51780
C	2.73250	-6.83280	6.23040
H	-0.60010	-14.17880	4.31110
C	0.94220	-12.90110	5.06680
C	3.72500	-6.28540	7.02520
H	5.30430	-6.64260	8.43870
C	2.74020	-11.35950	5.96160
H	2.14340	-6.21510	5.56700
N	3.94990	-4.87440	6.96350
N	3.79980	-4.23000	5.81730
H	1.67790	-13.69640	5.05760
C	4.27870	-4.03200	7.96820
H	4.41430	-4.36470	8.98260
N	4.02490	-2.98810	6.06850
C	4.32830	-2.79980	7.37350
C	4.58930	-1.42140	7.77340
C	4.93730	-1.02260	9.04980
H	5.03860	-1.75370	9.84090
N	4.45750	-0.54640	6.75540
C	5.15660	0.32350	9.29220
C	4.66810	0.74690	7.00040
C	5.01870	1.22380	8.25000
H	5.43320	0.66290	10.28240
H	4.54980	1.42140	6.16280
H	5.17970	2.28310	8.39560
H	3.20710	-12.33340	6.11200
H	3.27920	-10.90530	5.12670

7.1.4 2-*E* (in Co_2(2-*E*)$_3$)

58

ENERGY = -7644.499062571830 Input = wB97X-D3 def2-TZVP def2/J D3ZERO RIJCOSX OPT

H	4.23450	-14.43950	4.07980
C	5.04280	-14.08190	4.70270
H	6.62150	-14.97350	3.54670
C	6.36500	-14.37530	4.41200
C	4.76470	-13.31440	5.81610
C	7.35530	-13.89120	5.24730
H	3.74580	-13.06310	6.07870
N	5.71400	-12.84980	6.63380
H	8.39830	-14.09500	5.04470
C	6.99940	-13.13520	6.35230
C	7.97420	-12.62100	7.31250
C	9.28680	-12.91050	7.57730
H	10.00690	-13.57260	7.12710
N	7.55710	-11.74740	8.25860
N	9.56830	-12.19070	8.68260
N	8.51020	-11.49320	9.08690
H	12.03800	-11.56260	7.88120
C	11.97620	-11.92380	8.90060
C	15.60680	-12.22090	11.58850
H	16.25990	-12.45260	12.42990
C	10.76550	-12.25380	9.46520
N	14.31480	-11.76250	8.83920
C	15.34330	-10.74510	11.54590
C	13.15230	-12.07690	9.63410
N	15.10750	-10.86540	9.11290
C	15.39340	-9.95150	12.68530
C	15.04780	-10.12280	10.33420
H	15.60110	-10.41210	13.64390
C	10.69420	-12.74220	10.75890
C	15.19950	-8.58420	12.62370
C	13.12470	-12.63480	10.91590

C	14.92050	-8.74580	10.23430
H	9.73910	-13.00750	11.19280
C	11.86840	-12.92730	11.45560
C	14.99610	-7.98930	11.38850
H	15.22910	-8.00180	13.53480
C	14.32380	-13.04140	11.73940
H	14.79660	-8.27930	9.26700
N	14.94000	-6.56090	11.29600
N	14.49540	-5.96780	10.19860
H	11.82340	-13.34500	12.45420
C	15.38090	-5.65740	12.20070
H	15.81420	-5.93370	13.14480
N	14.64180	-4.70140	10.38270
C	15.17890	-4.44730	11.59920
C	15.42930	-3.04820	11.92240
C	15.92850	-2.59610	13.12930
H	16.15570	-3.29630	13.92210
N	15.12550	-2.21360	10.90870
C	16.12870	-1.23650	13.30120
C	15.32090	-0.90620	11.08500
C	15.82000	-0.37740	12.26060
H	16.51990	-0.85400	14.23550
H	15.06840	-0.26340	10.25270
H	15.96110	0.69080	12.35210
H	14.01520	-13.02020	12.78620
H	14.55000	-14.08810	11.51580
H	16.14750	-12.52210	10.69070

7.1.5 1-*E* (free optimization)

58

ENERGY = -1626.504907121961 Input = wB97X-D3 def2-TZVP def2/J D3ZERO RIJCOSX OPT

H	-24.25652045485303	-19.29341548168389	13.36229471521733
C	-23.65286523790430	-18.47856865599104	13.74068059004637
H	-24.92026063296894	-18.02859021624352	15.42015220083576
C	-24.01572133794122	-17.77868245138149	14.87859661754813
C	-22.48809216854827	-18.10476133797534	13.08704882348581
C	-23.20759439165550	-16.74259861265539	15.30697932007119
H	-22.16843901720011	-18.62956950751598	12.19176799592848
N	-21.69878481702075	-17.11926677830183	13.49395542597154
H	-23.47307548493048	-16.15472276579384	16.17679475938371
C	-22.05194261824618	-16.44621042581080	14.58663749891817
C	-21.16214164998551	-15.36346203143823	15.03865168305010
C	-20.91111635827063	-14.94836824106997	16.31868890890762
H	-21.23594838469085	-15.31316818079973	17.27740950796698
N	-20.42430703499662	-14.58825645849897	14.19705105041961
N	-20.03169970846413	-13.93406607418070	16.18006877162882
N	-19.75258046899531	-13.73609175025064	14.88452742286368
C	-19.42226728379860	-13.14792490033310	17.19122254492475
C	-20.12260866060481	-12.86750478753755	18.35338560215686
C	-18.13669699124719	-12.66404806867707	16.99172164063959
C	-19.51211222023850	-12.12024943855257	19.34579053591296
H	-21.14418313009433	-13.20443592228900	18.47259004273310
C	-17.50408756940298	-11.91887553726559	17.97486251880705
H	-17.63800018731882	-12.87732295528547	16.05459855663575
H	-20.03920548647245	-11.87267481100331	20.25821021308300
C	-18.21295621122825	-11.69186594176443	19.16406549974487
C	-16.09270141926635	-11.38103855982290	17.73852824119434
N	-16.46759087068848	-11.35956746593486	20.45242069827162
C	-15.75364920275504	-9.92718215695104	18.18149844384980
H	-15.37206287403002	-12.07102876237862	18.18373692320400
H	-15.92385159937688	-11.43286839328105	16.66197023295027
N	-17.51815729765134	-10.84012891446836	20.06066195701697
C	-15.07543668818864	-9.70565251781117	19.53298136019560
H	-15.07735668977116	-9.51663485581690	17.43031014496994

H -16.65514153081469 -9.31238172343894 18.13407421377974
C -15.46668330242277 -10.38390825550297 20.69729767994181
C -14.01614317487157 -8.81807152839192 19.64091557074578
C -14.81614862550551 -10.22081471776316 21.90268527576573
C -13.39130294123645 -8.60900959671312 20.86357221621353
H -13.66523096778089 -8.27121740501345 18.77527070940216
C -13.78600991648271 -9.30065512134520 21.99746122384773
H -15.13360009852112 -10.78835286497478 22.76794466074214
H -13.30560332319032 -9.12014356504913 22.95007653440266
N -12.32681148391860 -7.67470102284324 20.94207344647902
N -12.27269742247311 -6.62769270666174 20.10624755802190
C -11.29299535764848 -7.63230644241222 21.80854658831670
N -11.23879461430069 -5.92928862130960 20.40653767306022
C -10.59445476864587 -6.50735833279793 21.45814270962778
H -11.11368872195378 -8.40132388660247 22.53920312588323
C -9.37845487995651 -5.94421183528788 22.06800944870182
N -8.63121371957514 -5.14127776656002 21.31466376707625
C -9.04411668009249 -6.25569332605483 23.38517371393122
C -7.53722651974435 -4.61879429537353 21.85227319416258
C -7.89532723765411 -5.71405586782132 23.92987624509856
H -9.68589617695974 -6.89538953883283 23.97810144788096
C -7.12081884101014 -4.87180215656971 23.15031859018400
H -6.95822306296734 -3.96446812932426 21.20754996474979
H -7.61259444958535 -5.94316071752703 24.95038190590399
H -6.21460803388129 -4.42151761706350 23.53434208754507

7.1.6 1-*Z* (free optimization)

58
ENERGY = -1626.518811203468 Input = wB97X-D3 def2-TZVP def2/J D3ZERO RIJCOSX OPT
H -11.46271519084897 -26.09299646194644 11.37353952991668
C -11.65056532968222 -25.24845695960826 12.02371415383324
H -9.70600832186620 -24.35867343764932 11.79210209614131
C -10.68275671231958 -24.28591029129419 12.25510080698261
C -12.87681225685568 -25.10477256583458 12.65447065900631
C -10.97983340275501 -23.23312105334400 13.10036833440373
H -13.66168338941271 -25.83948630977478 12.50166241633973
N -13.17782505739294 -24.09481578151678 13.45883927754948
H -10.23693071331076 -22.47758449202298 13.32367157932433
C -12.24523728810105 -23.17135792987675 13.68083756562072
C -12.60564457730739 -22.06044396866128 14.57716561940281
C -12.09429677545268 -20.79082900896795 14.63855687858580
H -11.35691757521581 -20.27068106872282 14.05235819944632
N -13.57491873232842 -22.16364639743034 15.52790713651566
N -12.79103189922256 -20.19170405907197 15.62687072408682
N -13.68095676700003 -21.04783525691990 16.15145199948483
C -12.68984301818579 -18.87073629884341 16.12949873332254
C -13.82707078476348 -18.23677466596021 16.60171362700235
C -11.46232518387267 -18.23098048097945 16.15869326435566
C -13.70823851097738 -16.96523815726652 17.12576558406994
H -14.78134731539985 -18.74289555262558 16.56126653520625
C -11.32719722789963 -16.94775323642654 16.67556782892363
H -10.57904219233239 -18.74739046707352 15.80168008767511
H -14.58062695839421 -16.44222391160839 17.49734018032399
C -12.47090965078486 -16.34165455020032 17.19342936153872
C -9.97554211206156 -16.27776177538876 16.65605559548020
N -12.46612542776368 -15.01891757052230 17.76310310243673
C -9.44853280723107 -15.74405313874404 18.01497656698555
H -9.26044498154651 -16.99894060117392 16.25903222308099
H -10.00346738569359 -15.44883618083946 15.94405644079646
N -11.99654091458021 -14.83972602208801 18.88850901402210
C -10.11310255513364 -16.36497059423238 19.20999314160930
H -8.37428505510321 -15.92000925104891 18.06802217042978
H -9.59053287898931 -14.66322488882117 18.05929682049053
C -11.38350640084337 -15.93444046885824 19.59045078548980

C -9.52027926568343 -17.38786581534871 19.93126949859510
C -12.02389989819318 -16.47002119066099 20.69361628160836
C -10.17932886048042 -17.95775620313659 21.00973898773016
H -8.53921448342604 -17.75506096587224 19.65901886221393
C -11.42460029315512 -17.49551392935683 21.40358936322378
H -12.98956497987239 -16.08409209083824 20.99476523986032
H -11.91554958217523 -17.91262501735920 22.27320391146760
N -9.56076879501875 -19.01969785881838 21.71512219131488
N -8.22512808372921 -19.10051942964919 21.80415886137675
C -10.13509039990009 -20.06228222669109 22.35058849199584
N -7.93410823818793 -20.15842261103429 22.46989685356525
C -9.08252859237128 -20.79236229349744 22.83493876556243
H -11.19897080334760 -20.22285688215650 22.35981487518331
C -9.10345964056207 -22.04410626529966 23.60943313274686
N -8.03454752680068 -22.83214281024169 23.52298940423025
C -10.21251361459199 -22.37128186120858 24.38753628485273
C -8.04065907121624 -23.96774388183407 24.20798204743588
C -10.21072553863374 -23.56361247583801 25.08674356191760
H -11.05376311324156 -21.69274195232504 24.45486085720310
C -9.09963765473476 -24.38513035089656 24.99965979077033
H -7.14891777832089 -24.58045733831490 24.11647759913800
H -11.06063657261548 -23.84237175919154 25.69809895448081
H -9.04969186311239 -25.32629193508435 25.53152814364471

7.1.7 2-*E* (free optimization)

58
ENERGY = -1626.504426802891 Input = wB97X-D3 def2-TZVP def2/J D3ZERO RIJCOSX OPT
N -6.50064733686131 -10.93130649983989 5.13158670405925
C -6.75031971924383 -12.22359801408555 5.43212926204707
N -6.46744166731871 -10.76404837990769 3.80140104227322
N -6.68294958154097 -11.90294273890152 3.25056938096182
C -6.86693840335608 -12.84347626420977 4.21815432113924
C -7.13592940028759 -14.26245820728650 3.93367098044798
N -6.55904150330774 -14.79018133595202 2.85645560763583
C -6.78109976799494 -16.07169924410977 2.59709997865181
C -7.56872060104569 -16.89341022558445 3.38929097265833
C -8.16804215390437 -16.34097657624997 4.50797688231012
C -7.95530734097771 -15.00301241148964 4.78302956189703
H -6.29910693061706 -16.46613577422450 1.70767670497220
H -7.70924995101259 -17.93468176100366 3.12993673874522
H -8.80049780244640 -16.94091011712433 5.15120541559219
H -8.43115350789269 -14.52841238352511 5.63216278796283
H -6.77221903690488 -12.59705372071074 6.44068372600234
C -6.27213620982177 -9.84305359288032 6.00909232484198
C -5.54954901890526 -8.74383569791975 5.57142860117374
C -6.77579928301065 -9.88297914728902 7.29922925071509
H -5.18195000384836 -8.72173173096187 4.55526778627819
C -5.32516683610965 -7.68547842302203 6.43650932262857
H -7.38692953066716 -10.70539226628102 7.64689272954771
C -6.48244547041890 -8.84017386267218 8.15309094226756
C -5.77074511764424 -7.70286343370406 7.75262411449779
H -4.77474830496899 -6.82400169339698 6.07537909385545
N -7.01688935051100 -8.72423056237598 9.46230194796989
C -5.49042488131088 -6.52417037842980 8.68343675888424
N -6.11687883074301 -8.64116385198165 10.30355899967154
C -6.62078629042434 -6.00136175789035 9.61753668320167
H -4.60249946781803 -6.74445213458020 9.28081929616617
H -5.21041543104540 -5.69070329440794 8.03674050105583
C -6.50333555285931 -7.84484614393647 11.41072300059886
C -6.69827557580499 -6.50152133327328 11.05931529701196
H -6.49705475237767 -4.91861991564673 9.68079336030661
H -7.58789236563205 -6.15875820930323 9.13337110618062
C -6.53088834548378 -8.29815419013452 12.71119935382317
C -6.96152432104991 -5.62305234613221 12.10090082790962

C -6.81574588588446 -7.38801688869396 13.71738103648682
H -6.34146744728512 -9.33612239076832 12.94570325040067
C -7.02767235490924 -6.05192285573554 13.41836484227093
H -7.10818380773569 -4.57086409074942 11.88465417106601
H -7.20826528529917 -5.33510973287964 14.20889091802747
N -6.82589070737394 -8.57350733297288 19.47549125314605
N -6.38224522986919 -9.12602857180637 16.68117440468897
C -7.20733475248142 -8.53965562730993 20.74474362791207
C -7.67389339083401 -8.11860564954254 18.55571070839361
C -7.22674626501748 -8.16793745858960 17.15386476816239
N -6.17501242979183 -8.93075366904417 15.43097192488728
C -8.43303651253100 -8.04910867722932 21.16827780388991
H -6.49045624801829 -8.92204521917897 21.46503272646335
C -8.93139683969640 -7.61426268870960 18.88070179686149
C -7.54358832572692 -7.33880090053229 16.11170723331100
N -6.86738218620707 -7.84402703782151 15.05797918936520
H -8.68837614488652 -8.04470365704686 22.21996309448264
C -9.31173338654979 -7.57444484255831 20.20944221161002
H -9.60517060859793 -7.28099287075886 18.10118486545450
H -8.17162755996364 -6.46823328935890 16.04065327050304
H -10.28467498617172 -7.19000892828666 20.49116553667218

7.1.8 2-*Z* (free optimization)

58
ENERGY = -1626.517445797561 Input = wB97X-D3 def2-TZVP def2/J D3ZERO RIJCOSX OPT
H -1.75774064402819 -13.80926857217354 -0.18656547434929
C -1.87387522675611 -14.07818412564171 0.85524818698340
H -0.71301189543067 -15.88597041852171 0.74674198652350
C -1.29751338311412 -15.22487996804356 1.37517878744086
C -2.61325061161964 -13.27680155126406 1.71100097948712
C -1.48433821399274 -15.51599582184292 2.71256407122429
H -3.08305929811458 -12.36942835441692 1.34344660350940
N -2.79389423453730 -13.54165648299997 2.99779018809834
H -1.05890162850117 -16.41464075491043 3.14103611125267
C -2.23915077752336 -14.64330385040665 3.49593165892368
C -2.44959856068719 -14.91800376282651 4.92727752028123
C -1.83885387859809 -15.84890046516296 5.72529394610397
H -1.08846701916644 -16.59518810218348 5.53407080640386
N -3.35220807385760 -14.24504751800340 5.69461651086597
N -2.40975246902017 -15.68755355826819 6.93709749034685
N -3.32691153625167 -14.70961176224908 6.88943812981916
C -2.17165587082293 -16.40291143046169 8.13603175526353
C -3.16621975626270 -16.49099202351169 9.09720436121893
C -0.95582853546792 -17.02689878485901 8.33961851661287
H -4.11431104526161 -15.99761861190326 8.93720151879979
C -2.92031852084161 -17.21876756449774 10.24433936574918
H -0.16152923252551 -16.96314006695397 7.60701717060789
C -0.72454393324664 -17.73452623900900 9.51051522765037
C -1.71239250658831 -17.87161597136648 10.48489474719427
H -3.70316532165865 -17.29252817456098 10.99032955660178
N 0.56869594820154 -18.37018713347788 9.55963925090923
C -1.57583885179666 -18.70078611033316 11.73987475942114
N 1.44135774652139 -17.96964748807705 10.32924428598571
C -0.25427172011959 -18.57439641023950 12.52844586754068
H -2.40178261254061 -18.41913267437981 12.39473543977243
H -1.73345878448228 -19.75419995387950 11.49054314917082
C 1.16469125041307 -16.93246034540736 11.28288025889438
C 0.39114884812338 -17.22488881023530 12.40254586662851
H -0.45856665374360 -18.78497192408331 13.57879049331165
H 0.44939958923013 -19.33754422115715 12.19259366676628
C 1.79319472247752 -15.70902488548566 11.12590136219498
C 0.24168122013316 -16.22728296438445 13.35720206335719
C 1.61869438901272 -14.73564478676988 12.09578180177875
H 2.39744865472190 -15.53091811569588 10.24590044197806
C 0.84132162847746 -14.98960994274131 13.21692049775177

```
H   -0.35519931513507    -16.42765030876382     14.23975864652715
H    0.72201227917907    -14.22034280885013     13.96619542186426
N    2.25234044258711    -13.47561698689679     11.96132080148382
C    3.32529792117293    -13.12889299737944     11.21656499142704
N    1.81690462071530    -12.41755972015571     12.66192670584184
C    3.52675914849077    -11.80574035213536     11.49781472769319
H    3.87106638906505    -13.82734508743746     10.60725973415398
N    2.57444426478874    -11.41920982657487     12.39155212051311
C    4.55893365909160    -10.89097430285128     10.98256594420076
C    4.99756689634434    -10.99197390683551      9.66451392231779
N    5.04118612831006     -9.98576376829476     11.83147592199658
C    5.97987958563065    -10.12560761851509      9.21984157520504
H    4.55646983768850    -11.72218807673692      8.99741295349096
C    5.97427269569178     -9.15417139461208     11.38896570994621
C    6.48315855404579     -9.18298228890932     10.09931277756351
H    6.33880442839333    -10.17812247987915      8.19897881280517
H    6.33919905956472     -8.42628200350541     12.10721979464716
H    7.24828020362069     -8.47984636927937      9.79690051024639
```

7.2 Xyz files for the self-assemblies

7.2.1 ΔΛ-Co_2(1-*Z*)$_3$

```
176
 ENERGY = -7644.495581080593 Input = wB97X-D3 def2-TZVP def2/J D3ZERO RIJCOSX OPT
C   -6.81946285991986     -6.15740833713036      7.24405793113718
C   -5.87817871127667     -7.09243062891592      7.64004460320574
C   -4.94232268179767     -6.81647273912391      8.62215720346070
C   -4.94300818336395     -5.57929277828130      9.25390198282338
C   -5.91276617265649     -4.65255600024141      8.86926594603188
C   -6.84371299879283     -4.93144988737957      7.87886464493453
H   -7.54984396034000     -6.40604121385531      6.48569216107717
H   -7.60696871774961     -4.20104039433107      7.64237329514789
H   -4.21434195300632     -7.56850534938519      8.90558486477509
C   -2.78073469569736     -4.36726677862270      9.90188351100364
C   -3.97792655239167     -5.22736119033184     10.35114313133475
H   -2.33628737952297     -3.92229522893741     10.79676261732024
H   -2.01859411669339     -5.02812476016684      9.48382149483303
N   -6.09420657945119     -3.47965041268558      9.66296491153444
N   -5.29096511321874     -2.55417621479157      9.66223590645042
C   -3.00900364578262     -3.26836139596773      8.88516147782237
C   -4.17022667024142     -2.50225678372775      8.76600932802198
H   -3.58054614867538     -6.14024594005536     10.79579370954941
H   -4.52199496335889     -4.71633915706467     11.14672497890913
C   -4.25976241396994     -1.47542982190521      7.83177397199932
C   -3.20994667796996     -1.19197210860194      6.98726279678274
C   -1.94223901352361     -2.94192058221038      8.04789872803288
C   -2.03741055888638     -1.91748507848141      7.12535830430222
H   -5.16739653323921     -0.88635717240415      7.79232762202721
H   -1.01439492654408     -3.49272278632336      8.12898799483349
H   -3.31490279970777     -0.40664703417535      6.25098018245174
N   -5.92728109228875     -8.40416296002327      7.06446493185472
N   -0.89870641559473     -1.57338499131716      6.32831907075992
N    0.11830316452422     -2.41762427996874      6.18180993238791
C   -0.65645814010507     -0.41008550930801      5.68754295510041
N    1.00366211697402     -1.82318306651682      5.46019880435621
C   -6.09359508530687     -9.57514052264082      7.71784123955040
N   -5.88933736647164     -8.57734904981253      5.75216323039208
N   -6.02561743271412     -9.84295831810967      5.55171235072245
C   -6.15745843133978    -10.51141144327861      6.72221272602118
C   -6.35947311381846    -11.95250340044870      6.62553020983342
C    0.57870583903146     -0.58372588932114      5.12259364263913
C    1.45134625318376      0.26804025311070      4.31722952949867
N    2.66879303352971     -0.24223457445008      4.05419611142349
C    1.07104404000856      1.52026238677114      3.86229276484625
C    1.96243075359558      2.26700657380784      3.11300376609503
```

C	3.52020666927194	0.48097175533435	3.32096274690731
C	3.21157800816666	1.73568045632739	2.83463707896008
H	3.94084135991242	2.28253361150559	2.25290017220805
H	1.68587632764794	3.24972806610815	2.75243774842189
H	0.08686483616807	1.90468331410687	4.09466871953448
H	4.48651248015969	0.03133135415296	3.13561749881027
N	-6.35402222926949	-12.39991915073658	5.35401988951836
C	-6.55167943807957	-13.70089043477229	5.13867493749982
C	-6.76550737570442	-14.60378619460770	6.16372116643227
C	-6.77256397249081	-14.14165942867102	7.46876608884553
C	-6.56312145431767	-12.79271183728752	7.70413097876674
H	-6.54070692646325	-14.02531414254614	4.10670097638038
H	-6.92809431863725	-15.64825136353543	5.93547241812210
H	-6.94269071578019	-14.82211852057698	8.29351996283220
H	-6.56706868219763	-12.39688954387750	8.71100007796369
H	-6.17958166873868	-9.62764464298798	8.78964088554236
H	-1.33392409723532	0.42464689010463	5.71187747231465
H	-11.46662836418054	-10.79696644964418	4.62741193359623
C	-10.60746005273972	-10.33963460005611	4.15570867329847
H	-11.68629087673528	-8.68151998820470	3.31831148568983
C	-10.72485022705956	-9.16738521635805	3.42756311737196
C	-9.35770963995650	-10.92499376199232	4.26633918535454
C	-9.59491560847905	-8.62383434942087	2.83793006109440
H	-9.22016885636138	-11.84379286340776	4.82627350090815
N	-8.26913920178695	-10.41543660421408	3.69719728768141
H	-9.65564532409829	-7.70711516500434	2.26591575919581
C	-8.38612723015132	-9.28159028193199	2.99393267827906
C	-7.12916837494316	-8.82064102422732	2.39657502049127
C	-6.80323122644950	-7.86498854664392	1.47265862718482
H	-7.38542516055609	-7.15337449886376	0.91290537718477
N	-5.97621887236804	-9.46432251995094	2.70628776168282
H	-2.83333093725713	-2.97472517714472	0.83244299673635
N	-5.47265400259235	-8.00833719653908	1.29063761490940
N	-4.98179423417847	-8.98036081128098	2.04035054968258
H	-5.22039140102692	-5.38210412785164	1.04352723001890
C	-4.66353296459062	-5.94068956759211	0.29945324989403
C	-2.71496861612086	-3.07829972723326	-0.24826284158732
H	-2.68514960421527	-2.06044960791037	-0.64774045016035
C	-4.65740577367855	-7.32442046457879	0.33259822720567
N	-1.80152281707132	-4.64502734161055	-2.77473376618752
C	-1.00047959320512	-4.47590347097992	-1.59464580502576
C	-3.97048452290520	-5.26639758718138	-0.69771242403568
N	-2.77999676137152	-5.38161316999852	-2.81129546548472
C	0.28456502867448	-4.99366221208990	-1.72340591177943
C	-1.36417697563353	-3.72188343639849	-0.47679904357034
H	0.53303905530447	-5.55430142088743	-2.61577614114892
C	-3.95305791343287	-8.07441913560965	-0.59468778640542
C	1.23650155885934	-4.77779431396744	-0.75265892075510
C	-3.28955952301870	-6.03015591457164	-1.64659191727827
C	-0.37566689177900	-3.47912782498860	0.47653489220859
H	-3.97982304395387	-9.15528875952270	-0.55452618446805
C	-3.27734331362250	-7.41689495670569	-1.60329706496789
C	0.90216698064916	-3.98225184566450	0.33179313223460
H	2.22240894424111	-5.20847603770995	-0.86425657520988
C	-3.97014072384446	-3.76739134756255	-0.81369161860695
H	-0.60802015473647	-2.86797621984615	1.33906157623709
N	1.91679938909256	-3.63175735671213	1.27988746474193
N	1.61017306302091	-3.18013359607389	2.48444045965589
H	-2.77417471727740	-7.97058422804413	-2.38568904402303
C	3.25376094692147	-3.63256213257761	1.08231675771415
H	3.70780455287096	-3.92163868449476	0.15162824938369
N	2.72601475713067	-2.89151326038764	3.06664023929840
C	3.77753134967066	-3.15490531907020	2.25123431112572
H	-4.09876329879515	-3.49787820863665	-1.86286091136966
H	-4.83532901114202	-3.35881447071903	-0.29054438982325
C	5.13895778898174	-2.87087345593616	2.71226997024956
C	6.27465810570523	-3.21768021621348	1.99928484857995

H	6.19735277892906	-3.73431104809059	1.05156704898822
N	5.19381735072573	-2.23430617760993	3.88890861982157
C	7.51298667673468	-2.88802718148310	2.52576022240281
C	6.38746905559475	-1.92665097722809	4.38926172823923
C	7.57363431861592	-2.23059177751589	3.74349120057254
H	8.41920769361599	-3.14096614157764	1.98998862299533
H	6.38856631979215	-1.40943403410470	5.34277448460556
H	8.52084989316787	-1.95260564295800	4.18540289744549
H	-7.65061047384904	-13.69976500984964	-0.03806398410805
C	-6.77893341833452	-13.41589553344047	0.53546135624883
H	-5.36077124688516	-14.47415097708956	-0.68541909608459
C	-5.50892404037106	-13.84141375069049	0.18065201025819
C	-6.91863970351620	-12.61171989914629	1.64882664941993
C	-4.42991166313918	-13.44738108629051	0.95190410813587
H	-7.88987325460147	-12.25382763021033	1.96518198540404
N	-5.88249316412717	-12.23122330094835	2.40128872795111
H	-3.42611974462938	-13.76211924320164	0.69940786430555
C	-4.64703854880406	-12.64172272113259	2.05809956396414
C	-3.57443271160836	-12.18817952479719	2.94308528790457
C	-2.23006428157907	-12.44256212463242	3.02769630847452
H	-1.55636516320947	-13.06127820245935	2.45992356885129
N	-3.88953335308825	-11.36520183743019	3.96970373710968
N	-1.82854650523820	-11.74804070103175	4.11248104128020
N	-2.84541634940745	-11.10115717241908	4.67430035851240
H	0.46244323381467	-11.21021596363898	2.88448365166340
C	0.58256012557023	-11.46198671362086	3.93243417033791
C	3.67257651162034	-9.83751467548502	3.84740205054487
H	4.69806792715003	-9.84540687017552	3.46702335082022
C	-0.52924292620220	-11.73382697231003	4.71138699632719
N	3.24965842371021	-12.15066174842018	6.37520087181216
C	3.79065559017709	-9.87464036295320	6.43535752775580
C	1.85407348795859	-11.51969037158722	4.48922845117223
N	4.08057390886715	-11.27407408384696	6.58233601065139
C	3.80479966975952	-9.18168704320064	7.64210147479577
C	3.67308046168310	-9.19798408070191	5.21966193403149
H	3.91687569375862	-9.74160363269504	8.56202994243544
C	-0.41644531819124	-12.05200110354123	6.05483824081967
C	3.70235242622940	-7.80947578068841	7.67392306606370
C	1.95893191256012	-11.86301338439616	5.83738732826602
C	3.61682131693504	-7.80480440024565	5.26824121957642
H	-1.29986202329385	-12.27539222846895	6.63758762269200
C	0.84261289906857	-12.13360706903333	6.61502257082374
C	3.64520332079192	-7.12552502924365	6.46985760215026
H	3.68608446638802	-7.29622923896033	8.62588251536985
C	3.10147417419293	-11.25834116954094	3.69153850944411
H	3.56308048674589	-7.24344860722774	4.34440642568277
N	3.65668800421549	-5.69262422866299	6.48042418844057
N	3.24658517760468	-4.99222714815977	5.43378548762461
H	0.97087108755581	-12.44181703098733	7.64482999334399
C	4.09102763238805	-4.88552993848849	7.47445587819458
H	4.49764270859793	-5.25892310647844	8.39703733533808
N	3.40514612926638	-3.74971811295118	5.73992473202105
C	3.91561405885386	-3.62091507192187	6.98717776948891
C	4.13902514758875	-2.26318771299740	7.46691918027354
C	4.65779893579450	-1.94378337706599	8.70799690967362
H	4.93267330796158	-2.72609594638694	9.40296423436780
N	3.78490866464716	-1.32308718249008	6.56877366883985
C	4.82060805759033	-0.60913016153999	9.04042904455504
C	3.94660898889219	-0.04103760087204	6.89926995736931
C	4.45755113321864	0.35880960778663	8.11976550112981
H	5.22588230278321	-0.32927489298046	10.00458901568713
H	3.65840006460000	0.69023451214176	6.15661030813958
H	4.56974676553866	1.41239362436960	8.33592092670638
Co	-6.04128563021382	-10.97383625546625	3.96132639631349
H	3.85771307545034	-11.99390616756266	3.96854261002367
H	2.89951151543368	-11.41876004252896	2.63193178814072
Co	3.09580417027484	-2.05507490294118	4.81332388894401

H 3.11142486022368 -9.16738378568532 3.19235597813359

7.2.2 ΔΛ-Co_2(2-*E*)$_3$

176
ENERGY = -7644.494445751221 Input = wB97X-D3 def2-TZVP def2/J D3ZERO RIJCOSX OPT
C -7.08871246323417 -7.10877744959611 8.31677118381358
C -6.42988863572757 -7.62576455863882 7.21601076731266
C -5.51701973234161 -6.87027515115024 6.50029552273749
C -5.24979453218249 -5.59398087113169 6.94318969544311
C -5.88876592246154 -5.01822972619021 8.05205187758107
C -6.81310523576031 -5.81056784455261 8.72104215388604
H -7.83502307671496 -7.68978886270527 8.84382336768764
H -7.34244143082088 -5.40464838532514 9.57476588332260
H -5.02059461616250 -7.27355538098928 5.62878334564305
C -5.60427333402669 -3.59459138648730 8.52653906449073
C -5.47065507239041 -2.44864326485617 7.48089636362646
H -4.71899010795014 -3.61078925306492 9.16675190428526
H -6.43146452667241 -3.32228694200580 9.18278409348154
N -4.42901281691143 -4.67235858626856 6.24464774366376
N -3.47364039723295 -4.30544351363486 6.93409146113043
C -3.09627984823471 -2.97149212163873 6.63514857102855
C -4.08586426186364 -2.03983601296066 6.98335808846613
H -6.10257670394820 -2.66713272754974 6.61683711256963
H -5.90401224268451 -1.55883941546182 7.93853901919398
C -3.75090959099889 -0.69807719517802 6.85601669710289
C -2.50312486542256 -0.29888209586097 6.40011777808670
C -1.83954454540751 -2.60941330350756 6.20295144896441
C -1.56423337322715 -1.25935186994797 6.06882617469346
H -4.47509773990380 0.05989464896085 7.12962298796723
H -1.09493381670412 -3.35707942590950 5.96847966186549
H -2.26409914466989 0.75533615706819 6.34030456314774
N -6.68860809308013 -8.96477880309885 6.78577103710656
N -0.27532900880022 -0.86835556608465 5.59423950591396
N 0.79694010876690 -1.59585914029784 5.89278064445671
C 0.05191892592666 0.18771167463886 4.82055572812869
N 1.80299748923167 -1.02785930499011 5.32915296363231
C -6.95206230151492 -10.05449640105991 7.54102499970278
N -6.63726865287614 -9.26611145394238 5.49792692321370
N -6.86124410177343 -10.53058694905198 5.41227057661117
C -7.06802829491909 -11.07362971987675 6.63440550712178
C -7.32177338951076 -12.50967528823318 6.66074923225024
C 1.40811673140749 0.07756697165991 4.65669212313934
C 2.41893769972769 0.85100793509707 3.93805255949424
N 3.66852966279196 0.35296863995855 3.99783559713765
C 2.13027523472282 2.01419657696965 3.24287272721237
C 3.15042279638391 2.68154357283654 2.58960205125977
C 4.64780648632889 1.00354323803292 3.36309600579926
C 4.43507681285784 2.16674552594438 2.65110595608417
H 5.26372924752991 2.65529553330031 2.15721954413764
H 2.94601800935802 3.59234694848880 2.04108998656576
H 1.11730480950754 2.39305042266086 3.21720927843735
H 5.63401215936898 0.56504292297247 3.43743760202750
N -7.25398553478016 -13.07654748232351 5.43867832777211
C -7.47235364173808 -14.38783402801260 5.33751883145053
C -7.76874334301316 -15.18335023407530 6.42908911785406
C -7.84222195316698 -14.59813782523322 7.68128461545495
C -7.61305795880964 -13.23728068009615 7.79919840324769
H -7.41015715995659 -14.81304176074539 4.34455141680880
H -7.94043508413491 -16.24206218008709 6.29106275492419
H -8.07673963647265 -15.19198012115728 8.55564540011646
H -7.66426436857905 -12.74620052188130 8.76190286139031
H -7.00269707649894 -10.01731085745173 8.61533980814115
H -0.68127654343840 0.88194669571464 4.44830425054185
H -12.29949716211000 -11.64838103003697 4.26347637038529
C -11.41884856121155 -11.20396350489386 3.81997838082299

H	-12.46581619264142	-9.61755987741044	2.81925853011611
C	-11.50638697175721	-10.07847452962885	3.01741601153411
C	-10.17018038510996	-11.75713245010611	4.04466514037241
C	-10.34926147245316	-9.54826578482627	2.47135445273894
H	-10.05490156969094	-12.63883737176931	4.66609172010340
N	-9.05399876750429	-11.25739368552757	3.52160608123162
H	-10.38777125557372	-8.66745207330404	1.84390650221496
C	-9.14170296848274	-10.16788676192010	2.74841774219963
C	-7.85396591944750	-9.69322125240961	2.23401651482349
C	-7.47642652378850	-8.66656259369522	1.41277788720054
H	-8.02884025180369	-7.89029115926917	0.91345972926325
N	-6.71477114976158	-10.32801468253134	2.60601899429277
H	-0.84968233387133	-5.32193679489482	-2.77551927954777
N	-6.13067466104174	-8.76301177036075	1.34284043873884
N	-5.68093576277641	-9.77201265975891	2.06971964029300
H	-3.61967860840876	-8.16632841019675	2.02247395325564
C	-3.95989408249214	-7.69103112033220	1.11280556051604
C	-1.12536615438999	-5.66107227913527	-1.77666891094822
H	-1.03430019445042	-6.74940376166213	-1.80445297383989
C	-5.23396524515859	-7.91680291551330	0.61824033702350
N	-1.77600641485659	-6.59713737232873	0.72556516773755
C	-0.06678644180826	-5.10158581096463	-0.83010884785527
C	-3.14273951394430	-6.82556418969894	0.41503660197280
N	-1.55106235122554	-5.40767328348665	0.96662953467591
C	1.13397234284641	-4.61997549119954	-1.33482182581701
C	-0.24435911797156	-5.02802613866943	0.56062611486720
H	1.30974495007341	-4.64292629636675	-2.40360707123659
C	-5.65969415362159	-7.33651332901048	-0.56331557944084
C	2.11035437361591	-4.08953933787968	-0.50664808615361
C	-3.52978912975039	-6.20526794946396	-0.78372468250153
C	0.69608217945257	-4.48168684547077	1.40948692173446
H	-6.63733091244385	-7.55904707899244	-0.97107192864258
C	-4.80548451667387	-6.48965564208546	-1.25174371637738
C	1.88308909692896	-4.03112714795160	0.85652573911400
H	3.02238304828552	-3.69262241034935	-0.93413155505354
C	-2.61560137448868	-5.26692011588579	-1.56667714847829
H	0.51472647090210	-4.41558517817811	2.47355472863376
N	2.88603579416970	-3.47745311805444	1.71198937697198
N	2.54487558190004	-2.76459855657430	2.77181469241773
H	-5.14308489485578	-6.05148211576344	-2.18324717196759
C	4.22844671906943	-3.57165311829481	1.59164017889567
H	4.70203679681577	-4.13091398450181	0.80378818859568
N	3.64496674415193	-2.39866163268599	3.33647003725216
C	4.72020229597871	-2.86123926529053	2.65368083679006
H	-3.05702849042689	-5.16848053158975	-2.55875675879491
H	-2.67235825891184	-4.27018222949692	-1.12305819972335
C	6.06376654192015	-2.54784285343727	3.14927867106823
C	7.22669755958794	-3.00500696183572	2.55198541598715
H	7.18690143344242	-3.62756702724565	1.66764917910585
N	6.07352216527203	-1.77446704689490	4.24303686904438
C	8.44317018459538	-2.64739912246714	3.11004594528236
C	7.24608216464274	-1.44072249064117	4.77451900571840
C	8.45656771120439	-1.85143597265465	4.24342841016082
H	9.36958482820475	-2.98853203901813	2.66560831049047
H	7.20945621234533	-0.81532461947983	5.66021634659378
H	9.38556663237517	-1.54899463433639	4.70731655248623
H	-8.37097072913543	-14.93873308468737	0.22745082108792
C	-7.51361833772652	-14.60334456423555	0.79455017499641
H	-6.08914141729415	-15.88230241510748	-0.18390743234208
C	-6.24854201450886	-15.12095840806072	0.56919656303700
C	-7.66854416330623	-13.63901176115239	1.77025453115703
C	-5.18775203850715	-14.64893751256365	1.32100481911210
H	-8.63760700337530	-13.20731050619969	1.98306545213844
N	-6.65126625366195	-13.18468134004358	2.50676147235676
H	-4.18796667147870	-15.03230001159060	1.16678641114609
C	-5.41806940229627	-13.67630449412166	2.28045844338007
C	-4.36198740960830	-13.10759734536869	3.11650389290306

C -3.00443000923225 -13.27673999809825 3.19462236430558
H -2.30677554813301 -13.87957322425785 2.63998532319782
N -4.70182495482117 -12.17780692558599 4.03832110144717
H 2.19371852955385 -10.71078161274058 7.97136918554714
N -2.61950126662567 -12.43157163973826 4.17362901714054
N -3.65992795261130 -11.77265129011871 4.67454293032464
H -1.74958481739941 -10.13458927181563 5.17604173070334
C -0.99696053209686 -10.90912892930958 5.13063920486164
C 3.00234970637805 -10.53707181945070 7.25730648747403
H 3.89303755909762 -10.96348715665067 7.71977162134946
C -1.29826233191259 -12.17353028086053 4.65263145078011
N 0.71869424176968 -9.45489725275497 6.14457119678945
C 3.25234117365054 -9.03316887385088 7.16089198007544
C 0.29807571680363 -10.66297749685200 5.53147231541334
N 1.60586922311955 -8.89946927510101 5.49138107459602
C 4.25475437521050 -8.43746592192900 7.91575595538115
C 2.50345138115388 -8.18768480635080 6.32753934800716
H 4.87305754368082 -9.05200036202918 8.55902103516188
C -0.34600286011070 -13.17693474130477 4.63287393916188
C 4.49736971516923 -7.07276292126770 7.85892979763619
C 1.30573952059231 -11.63906434170093 5.51775703415441
C 2.73254270082448 -6.83282297968366 6.23035123473787
H -0.60006345275354 -14.17880704409909 4.31108169412052
C 0.94218230542600 -12.90111727709001 5.06676281924375
C 3.72500771437048 -6.28538463139636 7.02520241870079
H 5.30425926850955 -6.64255551698170 8.43868521410566
C 2.74022272750132 -11.35949974251278 5.96163287239020
H 2.14340784188094 -6.21508117639957 5.56702443962668
N 3.94985808539528 -4.87443379879439 6.96348480187722
N 3.79978731125301 -4.23001128427202 5.81726666813262
H 1.67789513227518 -13.69636500942481 5.05755267661192
C 4.27865280842046 -4.03197317087864 7.96823840406917
H 4.41429519041354 -4.36467798422226 8.98264116560405
N 4.02491594333601 -2.98809076221118 6.06845164024051
C 4.32831271574600 -2.79984711023274 7.37346905670221
C 4.58927359223083 -1.42140973810861 7.77342306499262
C 4.93727562031944 -1.02264273688812 9.04983263906424
H 5.03863688680589 -1.75371808779216 9.84087399187220
N 4.45754968531945 -0.54640541855721 6.75536611764149
C 5.15664708899103 0.32347836482122 9.29224848424884
C 4.66809611189835 0.74693277247735 7.00042311279382
C 5.01868073003017 1.22376433943646 8.25000247952820
H 5.43317380325340 0.66289008153935 10.28236583927071
H 4.54982265690241 1.42142427624551 6.16278406090102
H 5.17970693000462 2.28309110206194 8.39561263660023
Co -6.84724795726011 -11.78392282453246 3.93090342579761
H 3.20707068232982 -12.33338791347109 6.11200507079826
H 3.27917404621008 -10.90534694432658 5.12671460815017
Co 3.93291899795067 -1.37838659435623 4.98994367500863

7.2.3 ΛΛ-Co_2(1-*Z*)$_3$

176
ENERGY = -7644.495581080593 Input = wB97X-D3 def2-TZVP def2/J D3ZERO RIJCOSX OPT
C -6.81946285991986 -6.15740833713036 7.24405793113718
C -5.87817871127667 -7.09243062891592 7.64004460320574
C -4.94232268179767 -6.81647273912391 8.62215720346070
C -4.94300818336395 -5.57929277828130 9.25390198282338
C -5.91276617265649 -4.65255600024141 8.86926594603188
C -6.84371299879283 -4.93144988737957 7.87886464493453
H -7.54984396034000 -6.40604121385531 6.48569216107717
H -7.60696871774961 -4.20104039433107 7.64237329514789
H -4.21434195300632 -7.56850534938519 8.90558486477509
C -2.78073469569736 -4.36726677862270 9.90188351100364
C -3.97792655239167 -5.22736119033184 10.35114313133475
H -2.33628737952297 -3.92229522893741 10.79676261732024
H -2.01859411669339 -5.02812476016684 9.48382149483303

N	-6.09420657945119	-3.47965041268558	9.66296491153444
N	-5.29096511321874	-2.55417621479157	9.66223590645042
C	-3.00900364578262	-3.26836139596773	8.88516147782237
C	-4.17022667024142	-2.50225678372775	8.76600932802198
H	-3.58054614867538	-6.14024594005536	10.79579370954941
H	-4.52199496335889	-4.71633915706467	11.14672497890913
C	-4.25976241396994	-1.47542982190521	7.83177397199932
C	-3.20994667796996	-1.19197210860194	6.98726279678274
C	-1.94223901352361	-2.94192058221038	8.04789872803288
C	-2.03741055888638	-1.91748507848141	7.12535830430222
H	-5.16739653323921	-0.88635717240415	7.79232762202721
H	-1.01439492654408	-3.49272278632336	8.12898799483349
H	-3.31490279970777	-0.40664703417535	6.25098018245174
N	-5.92728109228875	-8.40416296002327	7.06446493185472
N	-0.89870641559473	-1.57338499131716	6.32831907075992
N	0.11830316452422	-2.41762427996874	6.18180993238791
C	-0.65645814010507	-0.41008550930801	5.68754295510041
N	1.00366211697402	-1.82318306651682	5.46019880435621
C	-6.09359508530687	-9.57514052264082	7.71784123955040
N	-5.88933736647164	-8.57734904981253	5.75216323039208
N	-6.02561743271412	-9.84295831810967	5.55171235072245
C	-6.15745843133978	-10.51141144327861	6.72221272602118
C	-6.35947311381846	-11.95250340044870	6.62553020983342
C	0.57870583903146	-0.58372588932114	5.12259364263913
C	1.45134625318376	0.26804025311070	4.31722952949867
N	2.66879303352971	-0.24223457445008	4.05419611142349
C	1.07104404000856	1.52026238677114	3.86229276484625
C	1.96243075359558	2.26700657380784	3.11300376609503
C	3.52020666927194	0.48097175533435	3.32096274690731
C	3.21157800816666	1.73568045632739	2.83463707896008
H	3.94084135991242	2.28253361150559	2.25290017220805
H	1.68587632764794	3.24972806610815	2.75243774842189
H	0.08686483616807	1.90468331410687	4.09466871953448
H	4.48651248015969	0.03133135415296	3.13561749881027
N	-6.35402222926949	-12.39991915073658	5.35401988951836
C	-6.55167943807957	-13.70089043477229	5.13867493749982
C	-6.76550737570442	-14.60378619460770	6.16372116643227
C	-6.77256397249081	-14.14165942867102	7.46876608884553
C	-6.56312145431767	-12.79271183728752	7.70413097876674
H	-6.54070692646325	-14.02531414254614	4.10670097638038
H	-6.92809431863725	-15.64825136353543	5.93547241812210
H	-6.94269071578019	-14.82211852057698	8.29351996283220
H	-6.56706868219763	-12.39688954387750	8.71100007796369
H	-6.17958166873868	-9.62764464298798	8.78964088554236
H	-1.33392409723532	0.42464689010463	5.71187747231465
H	-11.46662836418054	-10.79696644964418	4.62741193359623
C	-10.60746005273972	-10.33963460005611	4.15570867329847
H	-11.68629087673528	-8.68151998820470	3.31831148568983
C	-10.72485022705956	-9.16738521635805	3.42756311737196
C	-9.35770963995650	-10.92499376199232	4.26633918535454
C	-9.59491560847905	-8.62383434942087	2.83793006109440
H	-9.22016885636138	-11.84379286340776	4.82627350090815
N	-8.26913920178695	-10.41543660421408	3.69719728768141
H	-9.65564532409829	-7.70711516500434	2.26591575919581
C	-8.38612723015132	-9.28159028193199	2.99393267827906
C	-7.12916837494316	-8.82064102422732	2.39657502049127
C	-6.80323122644950	-7.86498854664392	1.47265862718482
H	-7.38542516055609	-7.15337449886376	0.91290537718477
N	-5.97621887236804	-9.46432251995094	2.70628776168282
H	-2.83333093725713	-2.97472517714472	0.83244299673635
N	-5.47265400259235	-8.00833719653908	1.29063761490940
N	-4.98179423417847	-8.98036081128098	2.04035054968258
H	-5.22039140102692	-5.38210412785164	1.04352723001890
C	-4.66353296459062	-5.94068956759211	0.29945324989403
C	-2.71496861612086	-3.07829972723326	-0.24826284158732
H	-2.68514960421527	-2.06044960791037	-0.64774045016035
C	-4.65740577367855	-7.32442046457879	0.33259822720567

N	-1.80152281707132	-4.64502734161055	-2.77473376618752
C	-1.00047959320512	-4.47590347097992	-1.59464580502576
C	-3.97048452290520	-5.26639758718138	-0.69771242403568
N	-2.77999676137152	-5.38161316999852	-2.81129546548472
C	0.28456502867448	-4.99366221208990	-1.72340591177943
C	-1.36417697563353	-3.72188343639849	-0.47679904357034
H	0.53303905530447	-5.55430142088743	-2.61577614114892
C	-3.95305791343287	-8.07441913560965	-0.59468778640542
C	1.23650155885934	-4.77779431396744	-0.75265892075510
C	-3.28955952301870	-6.03015591457164	-1.64659191727827
C	-0.37566689177900	-3.47912782498860	0.47653489220859
H	-3.97982304395387	-9.15528875952270	-0.55452618446805
C	-3.27734331362250	-7.41689495670569	-1.60329706496789
C	0.90216698064916	-3.98225184566450	0.33179313223460
H	2.22240894424111	-5.20847603770995	-0.86425657520988
C	-3.97014072384446	-3.76739134756255	-0.81369161860695
H	-0.60802015473647	-2.86797621984615	1.33906157623709
N	1.91679938909256	-3.63175735671213	1.27988746474193
N	1.61017306302091	-3.18013359607389	2.48444045965589
H	-2.77417471727740	-7.97058422804413	-2.38568904402303
C	3.25376094692147	-3.63256213257761	1.08231675771415
H	3.70780455287096	-3.92163868449476	0.15162824938369
N	2.72601475713067	-2.89151326038764	3.06664023929840
C	3.77753134967066	-3.15490531907020	2.25123431112572
H	-4.09876329879515	-3.49787820863665	-1.86286091136966
H	-4.83532901114202	-3.35881447071903	-0.29054438982325
C	5.13895778898174	-2.87087345593616	2.71226997024956
C	6.27465810570523	-3.21768021621348	1.99928484857995
H	6.19735277892906	-3.73431104809059	1.05156704898822
N	5.19381735072573	-2.23430617760993	3.88890861982157
C	7.51298667673468	-2.88802718148310	2.52576022240281
C	6.38746905559475	-1.92665097722809	4.38926172823923
C	7.57363431861592	-2.23059177751589	3.74349120057254
H	8.41920769361599	-3.14096614157764	1.98998862299533
H	6.38856631979215	-1.40943403410470	5.34277448460556
H	8.52084989316787	-1.95260564295800	4.18540289744549
H	-7.65061047384904	-13.69976500984964	-0.03806398410805
C	-6.77893341833452	-13.41589553344047	0.53546135624883
H	-5.36077124688516	-14.47415097708956	-0.68541909608459
C	-5.50892404037106	-13.84141375069049	0.18065201025819
C	-6.91863970351620	-12.61171989914629	1.64882664941993
C	-4.42991166313918	-13.44738108629051	0.95190410813587
H	-7.88987325460147	-12.25382763021033	1.96518198540404
N	-5.88249316412717	-12.23122330094835	2.40128872795111
H	-3.42611974462938	-13.76211924320164	0.69940786430555
C	-4.64703854880406	-12.64172272113259	2.05809956396414
C	-3.57443271160836	-12.18817952479719	2.94308528790457
C	-2.23006428157907	-12.44256212463242	3.02769630847452
H	-1.55636516320947	-13.06127820245935	2.45992356885129
N	-3.88953335308825	-11.36520183743019	3.96970373710968
N	-1.82854650523820	-11.74804070103175	4.11248104128020
N	-2.84541634940745	-11.10115717241908	4.67430035851240
H	0.46244323381467	-11.21021596363898	2.88448365166340
C	0.58256012557023	-11.46198671362086	3.93243417033791
C	3.67257651162034	-9.83751467548502	3.84740205054487
H	4.69806792715003	-9.84540687017552	3.46702335082022
C	-0.52924292620220	-11.73382697231003	4.71138699632719
N	3.24965842371021	-12.15066174842018	6.37520087181216
C	3.79065559017709	-9.87464036295320	6.43535752775580
C	1.85407348795859	-11.51969037158722	4.48922845117223
N	4.08057390886715	-11.27407408384696	6.58233601065139
C	3.80479966975952	-9.18168704320064	7.64210147479577
C	3.67308046168310	-9.19798408070191	5.21966193403149
H	3.91687569375862	-9.74160363269504	8.56202994243544
C	-0.41644531819124	-12.05200110354123	6.05483824081967
C	3.70235242622940	-7.80947578068841	7.67392306606370
C	1.95893191256012	-11.86301338439616	5.83738732826602

C 3.61682131693504 -7.80480440024565 5.26824121957642
H -1.29986202329385 -12.27539222846895 6.63758762269200
C 0.84261289906857 -12.13360706903333 6.61502257082374
C 3.64520332079192 -7.12552502924365 6.46985760215026
H 3.68608446638802 -7.29622923896033 8.62588251536985
C 3.10147417419293 -11.25834116954094 3.69153850944411
H 3.56308048674589 -7.24344860722774 4.34440642568277
N 3.65668800421549 -5.69262422866299 6.48042418844057
N 3.24658517760468 -4.99222714815977 5.43378548762461
H 0.97087108755581 -12.44181703098733 7.64482999334399
C 4.09102763238805 -4.88552993848849 7.47445587819458
H 4.49764270859793 -5.25892310647844 8.39703733533808
N 3.40514612926638 -3.74971811295118 5.73992473202105
C 3.91561405885386 -3.62091507192187 6.98717776948891
C 4.13902514758875 -2.26318771299740 7.46691918027354
C 4.65779893579450 -1.94378337706599 8.70799690967362
H 4.93267330796158 -2.72609594638694 9.40296423436780
N 3.78490866464716 -1.32308718249008 6.56877366883985
C 4.82060805759033 -0.60913016153999 9.04042904455504
C 3.94660898889219 -0.04103760087204 6.89926995736931
C 4.45755113321864 0.35880960778663 8.11976550112981
H 5.22588230278321 -0.32927489298046 10.00458901568713
H 3.65840006460000 0.69023451214176 6.15661030813958
H 4.56974676553866 1.41239362436960 8.33592092670638
Co -6.04128563021382 -10.97383625546625 3.96132639631349
H 3.85771307545034 -11.99390616756266 3.96854261002367
H 2.89951151543368 -11.41876004252896 2.63193178814072
Co 3.09580417027484 -2.05507490294118 4.81332388894401
H 3.11142486022368 -9.16738378568532 3.19235597813359

7.2.4 ΛΛ-Co_2(2-*E*)$_3$

176
ENERGY = -7644.494445751221 Input = wB97X-D3 def2-TZVP def2/J D3ZERO RIJCOSX OPT
C -7.08871246323417 -7.10877744959611 8.31677118381358
C -6.42988863572757 -7.62576455863882 7.21601076731266
C -5.51701973234161 -6.87027515115024 6.50029552273749
C -5.24979453218249 -5.59398087113169 6.94318969544311
C -5.88876592246154 -5.01822972619021 8.05205187758107
C -6.81310523576031 -5.81056784455261 8.72104215388604
H -7.83502307671496 -7.68978886270527 8.84382336768764
H -7.34244143082088 -5.40464838532514 9.57476588332260
H -5.02059461616250 -7.27355538098928 5.62878334564305
C -5.60427333402669 -3.59459138648730 8.52653906449073
C -5.47065507239041 -2.44864326485617 7.48089636362646
H -4.71899010795014 -3.61078925306492 9.16675190428526
H -6.43146452667241 -3.32228694200580 9.18278409348154
N -4.42901281691143 -4.67235858626856 6.24464774366376
N -3.47364039723295 -4.30544351363486 6.93409146113043
C -3.09627984823471 -2.97149212163873 6.63514857102855
C -4.08586426186364 -2.03983601296066 6.98335808846613
H -6.10257670394820 -2.66713272754974 6.61683711256963
H -5.90401224268451 -1.55883941546182 7.93853901919398
C -3.75090959099889 -0.69807719517802 6.85601669710289
C -2.50312486542256 -0.29888209586097 6.40011777808670
C -1.83954454540751 -2.60941330350756 6.20295144896441
C -1.56423337322715 -1.25935186994797 6.06882617469346
H -4.47509773990380 0.05989464896085 7.12962298796723
H -1.09493381670412 -3.35707942590950 5.96847966186549
H -2.26409914466989 0.75533615706819 6.34030456314774
N -6.68860809308013 -8.96477880309885 6.78577103710656
N -0.27532900880022 -0.86835556608465 5.59423950591396
N 0.79694010876690 -1.59585914029784 5.89278064445671
C 0.05191892592666 0.18771167463886 4.82055572812869
N 1.80299748923167 -1.02785930499011 5.32915296363231
C -6.95206230151492 -10.05449640105991 7.54102499970278
N -6.63726865287614 -9.26611145394238 5.49792692321370

N	-6.86124410177343	-10.53058694905198	5.41227057661117
C	-7.06802829491909	-11.07362971987675	6.63440550712178
C	-7.32177338951076	-12.50967528823318	6.66074923225024
C	1.40811673140749	0.07756697165991	4.65669212313934
C	2.41893769972769	0.85100793509707	3.93805255949424
N	3.66852966279196	0.35296863995855	3.99783559713765
C	2.13027523472282	2.01419657696965	3.24287272721237
C	3.15042279638391	2.68154357283654	2.58960205125977
C	4.64780648632889	1.00354323803292	3.36309600579926
C	4.43507681285784	2.16674552594438	2.65110595608417
H	5.26372924752991	2.65529553330031	2.15721954413764
H	2.94601800935802	3.59234694848880	2.04108998656576
H	1.11730480950754	2.39305042266086	3.21720927843735
H	5.63401215936898	0.56504292297247	3.43743760202750
N	-7.25398553478016	-13.07654748232351	5.43867832777211
C	-7.47235364173808	-14.38783402801260	5.33751883145053
C	-7.76874334301316	-15.18335023407530	6.42908911785406
C	-7.84222195316698	-14.59813782523322	7.68128461545495
C	-7.61305795880964	-13.23728068009615	7.79919840324769
H	-7.41015715995659	-14.81304176074539	4.34455141680880
H	-7.94043508413491	-16.24206218008709	6.29106275492419
H	-8.07673963647265	-15.19198012115728	8.55564540011646
H	-7.66426436857905	-12.74620052188130	8.76190286139031
H	-7.00269707649894	-10.01731085745173	8.61533980814115
H	-0.68127654343840	0.88194669571464	4.44830425054185
H	-12.29949716211000	-11.64838103003697	4.26347637038529
C	-11.41884856121155	-11.20396350489386	3.81997838082299
H	-12.46581619264142	-9.61755987741044	2.81925853011611
C	-11.50638697175721	-10.07847452962885	3.01741601153411
C	-10.17018038510996	-11.75713245010611	4.04466514037241
C	-10.34926147245316	-9.54826578482627	2.47135445273894
H	-10.05490156969094	-12.63883737176931	4.66609172010340
N	-9.05399876750429	-11.25739368552757	3.52160608123162
H	-10.38777125557372	-8.66745207330404	1.84390650221496
C	-9.14170296848274	-10.16788676192010	2.74841774219963
C	-7.85396591944750	-9.69322125240961	2.23401651482349
C	-7.47642652378850	-8.66656259369522	1.41277788720054
H	-8.02884025180369	-7.89029115926917	0.91345972926325
N	-6.71477114976158	-10.32801468253134	2.60601899429277
H	-0.84968233387133	-5.32193679489482	-2.77551927954777
N	-6.13067466104174	-8.76301177036075	1.34284043873884
N	-5.68093576277641	-9.77201265975891	2.06971964029300
H	-3.61967860840876	-8.16632841019675	2.02247395325564
C	-3.95989408249214	-7.69103112033220	1.11280556051604
C	-1.12536615438999	-5.66107227913527	-1.77666891094822
H	-1.03430019445042	-6.74940376166213	-1.80445297383989
C	-5.23396524515859	-7.91680291551330	0.61824033702350
N	-1.77600641485659	-6.59713737232873	0.72556516773755
C	-0.06678644180826	-5.10158581096463	-0.83010884785527
C	-3.14273951394430	-6.82556418969894	0.41503660197280
N	-1.55106235122554	-5.40767328348665	0.96662953467591
C	1.13397234284641	-4.61997549119954	-1.33482182581701
C	-0.24435911797156	-5.02802613866943	0.56062611486720
H	1.30974495007341	-4.64292629636675	-2.40360707123659
C	-5.65969415362159	-7.33651332901048	-0.56331557944084
C	2.11035437361591	-4.08953933787968	-0.50664808615361
C	-3.52978912975039	-6.20526794946396	-0.78372468250153
C	0.69608217945257	-4.48168684547077	1.40948692173446
H	-6.63733091244385	-7.55904707899244	-0.97107192864258
C	-4.80548451667387	-6.48965564208546	-1.25174371637738
C	1.88308909692896	-4.03112714795160	0.85652573911400
H	3.02238304828552	-3.69262241034935	-0.93413155505354
C	-2.61560137448868	-5.26692011588579	-1.56667714847829
H	0.51472647090210	-4.41558517817811	2.47355472863376
N	2.88603579416970	-3.47745311805444	1.71198937697198
N	2.54487558190004	-2.76459855657430	2.77181469241773
H	-5.14308489485578	-6.05148211576344	-2.18324717196759

C	4.22844671906943	-3.57165311829481	1.59164017889567
H	4.70203679681577	-4.13091398450181	0.80378818859568
N	3.64496674415193	-2.39866163268599	3.33647003725216
C	4.72020229597871	-2.86123926529053	2.65368083679006
H	-3.05702849042689	-5.16848053158975	-2.55875675879491
H	-2.67235825891184	-4.27018222949692	-1.12305819972335
C	6.06376654192015	-2.54784285343727	3.14927867106823
C	7.22669755958794	-3.00500696183572	2.55198541598715
H	7.18690143344242	-3.62756702724565	1.66764917910585
N	6.07352216527203	-1.77446704689490	4.24303686904438
C	8.44317018459538	-2.64739912246714	3.11004594528236
C	7.24608216464274	-1.44072249064117	4.77451900571840
C	8.45656771120439	-1.85143597265465	4.24342841016082
H	9.36958482820475	-2.98853203901813	2.66560831049047
H	7.20945621234533	-0.81532461947983	5.66021634659378
H	9.38556663237517	-1.54899463433639	4.70731655248623
H	-8.37097072913543	-14.93873308468737	0.22745082108792
C	-7.51361833772652	-14.60334456423555	0.79455017499641
H	-6.08914141729415	-15.88230241510748	-0.18390743234208
C	-6.24854201450886	-15.12095840806072	0.56919656303700
C	-7.66854416330623	-13.63901176115239	1.77025453115703
C	-5.18775203850715	-14.64893751256365	1.32100481911210
H	-8.63760700337530	-13.20731050619969	1.98306545213844
N	-6.65126625366195	-13.18468134004358	2.50676147235676
H	-4.18796667147870	-15.03230001159060	1.16678641114609
C	-5.41806940229627	-13.67630449412166	2.28045844338007
C	-4.36198740960830	-13.10759734536869	3.11650389290306
C	-3.00443000923225	-13.27673999809825	3.19462236430558
H	-2.30677554813301	-13.87957322425785	2.63998532319782
N	-4.70182495482117	-12.17780692558599	4.03832110144717
H	2.19371852955385	-10.71078161274058	7.97136918554714
N	-2.61950126662567	-12.43157163973826	4.17362901714054
N	-3.65992795261130	-11.77265129011871	4.67454293032464
H	-1.74958481739941	-10.13458927181563	5.17604173070334
C	-0.99696053209686	-10.90912892930958	5.13063920486164
C	3.00234970637805	-10.53707181945070	7.25730648747403
H	3.89303755909762	-10.96348715665067	7.71977162134946
C	-1.29826233191259	-12.17353028086053	4.65263145078011
N	0.71869424176968	-9.45489725275497	6.14457119678945
C	3.25234117365054	-9.03316887385088	7.16089198007544
C	0.29807571680363	-10.66297749685200	5.53147231541334
N	1.60586922311955	-8.89946927510101	5.49138107459602
C	4.25475437521050	-8.43746592192900	7.91575595538115
C	2.50345138115388	-8.18768480635080	6.32753934800716
H	4.87305754368082	-9.05200036202918	8.55902103516188
C	-0.34600286011070	-13.17693474130477	4.63287393916188
C	4.49736971516923	-7.07276292126770	7.85892979763619
C	1.30573952059231	-11.63906434170093	5.51775703415441
C	2.73254270082448	-6.83282297968366	6.23035123473787
H	-0.60006345275354	-14.17880704409909	4.31108169412052
C	0.94218230542600	-12.90111727709001	5.06676281924375
C	3.72500771437048	-6.28538463139636	7.02520241870079
H	5.30425926850955	-6.64255551698170	8.43868521410566
C	2.74022272750132	-11.35949974251278	5.96163287239020
H	2.14340784188094	-6.21508117639957	5.56702443962668
N	3.94985808539528	-4.87443379879439	6.96348480187722
N	3.79978731125301	-4.23001128427202	5.81726666813262
H	1.67789513227518	-13.69636500942481	5.05755267661192
C	4.27865280842046	-4.03197317087864	7.96823840406917
H	4.41429519041354	-4.36467798422226	8.98264116560405
N	4.02491594333601	-2.98809076221118	6.06845164024051
C	4.32831271574600	-2.79984711023274	7.37346905670221
C	4.58927359223083	-1.42140973810861	7.77342306499262
C	4.93727562031944	-1.02264273688812	9.04983263906424
H	5.03863688680589	-1.75371808779216	9.84087399187220
N	4.45754968531945	-0.54640541855721	6.75536611764149
C	5.15664708899103	0.32347836482122	9.29224848424884

C	4.66809611189835	0.74693277247735	7.00042311279382
C	5.01868073003017	1.22376433943646	8.25000247952820
H	5.43317380325340	0.66289008153935	10.28236583927071
H	4.54982265690241	1.42142427624551	6.16278406090102
H	5.17970693000462	2.28309110206194	8.39561263660023
Co	-6.84724795726011	-11.78392282453246	3.93090342579761
H	3.20707068232982	-12.33338791347109	6.11200507079826
H	3.27917404621008	-10.90534694432658	5.12671460815017
Co	3.93291899795067	-1.37838659435623	4.98994367500863

8. References

[1] M. Lehr, T. Paschelke, E. Trumpf, A.-M. Vogt, C. Näther, F. D. Sönnichsen, A. J. McConnell, *Angew. Chem. Int. Ed.* **2020**, *59*, 19344-19351.

[2] a) W. Moormann, D. Langbehn, R. Herges, *Synth* **2017**, *49*, 3471-3475; b) W. Moormann, D. Langbehn, R. Herges, *Beilstein J. Org. Chem.* **2019**, *15*, 727-732.

[3] M. S. Maier, K. Hüll, M. Reynders, B. S. Matsuura, P. Leippe, T. Ko, L. Schäffer, D. Trauner, *J. Am. Chem. Soc.* **2019**, *141*, 17295-17304.

[4] M. Lehr, T. Paschelke, V. Bendt, A. Petersen, L. Pietsch, P. Harders, A. J. McConnell, *Eur. J. Org. Chem.* **2021**, *2021*, 2728-2735.

[5] R. Siewertsen, H. Neumann, B. Buchheim-Stehn, R. Herges, C. Näther, F. Renth, F. Temps, *J. Am. Chem. Soc.* **2009**, *131*, 15594-15595.

[6] C. Bannwarth, E. Caldeweyher, S. Ehlert, A. Hansen, P. Pracht, J. Seibert, S. Spicher, S. Grimme, *WIREs Comput Mol Sci.* **2021**, *11*, e1493.

[7] C. Bannwarth, S. Ehlert, S. Grimme, *J. Chem. Theory Comput.* **2019**, *15*, 1652-1671.

[8] Y.-S. Lin, G.-D. Li, S.-P. Mao, J.-D. Chai, *J. Chem. Theory Comput.* **2013**, *9*, 263-272.

[9] F. Weigend, R. Ahlrichs, *Phys. Chem. Chem. Phys.* **2005**, *7*, 3297-3305.

[10] S. Grimme, J. Antony, S. Ehrlich, H. Krieg, *J. Chem. Phys.* **2010**, *132*, 154104.

[11] F. Weigend, *Phys. Chem. Chem. Phys.* **2006**, *8*, 1057-1065.

[12] F. Neese, F. Wennmohs, A. Hansen, U. Becker, *Chem. Phys.* **2009**, *356*, 98-109.

[13] M. Miklitz, K. E. Jelfs, *J. Chem. Inf. Model.* **2018**, *58*, 2387-2391.
